# LATS1 but not LATS2 represses autophagy by a kinase-independent scaffold function

**DOI:** 10.1038/s41467-019-13591-7

**Published:** 2019-12-17

**Authors:** Fengyuan Tang, Ruize Gao, Beena Jeevan-Raj, Christof B. Wyss, Ravi K. R. Kalathur, Salvatore Piscuoglio, Charlotte K. Y. Ng, Sravanth K. Hindupur, Sandro Nuciforo, Eva Dazert, Thomas Bock, Shuang Song, David Buechel, Marco F. Morini, Alexander Hergovich, Patrick Matthias, Dae-Sik Lim, Luigi M. Terracciano, Markus H. Heim, Michael N. Hall, Gerhard Christofori

**Affiliations:** 1 Department of Biomedicine, University of Basel, Basel, Switzerland; 2 Institute of Pathology, University Hospital Basel, Basel, Switzerland; 3 Biozentrum, University of Basel, Basel, Switzerland; 4 Friedrich Miescher Institute for Biomedical Research, Basel, Switzerland; 5 UCL Cancer Institute, University College London, London, WC1E 6BT UK; 6 Korea Advanced Institute of Science and Technology, Daejeon, 34141 Republic of Korea

**Keywords:** Targeted therapies, Hepatocellular carcinoma, Macroautophagy, Stress signalling

## Abstract

Autophagy perturbation represents an emerging therapeutic strategy in cancer. Although LATS1 and LATS2 kinases, core components of the mammalian Hippo pathway, have been shown to exert tumor suppressive activities, here we report a pro-survival role of LATS1 but not LATS2 in hepatocellular carcinoma (HCC) cells. Specifically, LATS1 restricts lethal autophagy in HCC cells induced by sorafenib, the standard of care for advanced HCC patients. Notably, autophagy regulation by LATS1 is independent of its kinase activity. Instead, LATS1 stabilizes the autophagy core-machinery component Beclin-1 by promoting K27-linked ubiquitination at lysine residues K32 and K263 on Beclin-1. Consequently, ubiquitination of Beclin-1 negatively regulates autophagy by promoting inactive dimer formation of Beclin-1. Our study highlights a functional diversity between LATS1 and LATS2, and uncovers a scaffolding role of LATS1 in mediating a cross-talk between the Hippo signaling pathway and autophagy.

## Abstract (web summary)

The autophagic and Hippo pathways are both well characterized contributors to cancer. Here, Tang et al show that LATS1, but not LATS2, negatively regulates autophagy by promoting Beclin1 ubiquitination, which restricts lethal autophagy induced by sorafenib treatment in cancer cells.

## Introduction

Liver cancer is the second leading cause of cancer-related mortality with hepatocellular carcinoma (HCC), representing about 90% of all cases of primary liver cancer^1^. Viral infections (hepatitis B or C), alcohol abuse, and metabolism-induced non-alcoholic fatty liver disease are major risk factors for HCC incidence^1^. Unlike other malignancies, HCC is often diagnosed only at advanced stages, where liver transplantation, surgical therapies, and resection are not available. Sorafenib (Srf), a multi-kinase inhibitor, is the standard-of-care treatment and the only effective systemic option for late-stage HCC, however, only with an average increased overall patient survival of ~3 months^2^. Apparently, HCC cells acquire resistance to Srf therapy. Therefore, understanding how HCC cells respond to Srf is important to improve the efficacy of Srf-based therapy in HCC patients; notably, to overcome the development of evasive resistance to Srf therapy.

Macroautophagy (hereafter referred to as autophagy) is a self-digestion process that targets the cytoplasmic components to the lysosomes for nutrient recycling in response to cellular stress and starvation^3^. Autophagy has been shown to play dual roles in tumor initiation and tumor progression^4,5^. Autophagy deficiency promotes tumor initiation but represses malignant tumor progression and, as a consequence, autophagy plays a conflicting role in cancer therapy, including the treatment of HCC with Srf^6^. Although acute treatment of HCC cells with Srf induces a lethal version of autophagy^7–9^, a survival version of autophagy is likely to mediate the adaptive response to Srf and promotes cell viability in Srf-resistant cells^10,11^. Thus, the biological consequence of autophagy activation in response to therapy is context-dependent and the mechanistic understanding of how autophagy is regulated in cancer appears of great importance to optimize therapeutic interventions.

The Hippo signaling pathway has emerged as a major growth control network regulating cell death, proliferation, and differentiation^12,13^. Activated Hippo or MAP4Ks phosphorylate and activate the downstream kinases Large Tumor Suppressor 1 and 2 (LATS 1 and 2), which in turn phosphorylate and inactivate the Hippo signaling transducers YAP and TAZ^14^. In short, Hippo signaling critically relies on a variety of protein kinase activities and protein phosphorylation events.

Here we report a kinase activity-independent role of LATS1, but not LATS2, in regulating therapy-induced autophagy in HCC cells. The results indicate that this scaffolding function of LATS1 plays a critical role in the regulation of autophagy and in therapy response of HCC cells and potentially other cancer type cells.

## Results

### Repression of Srf-induced cell death by LATS1

**Fig. 1:**
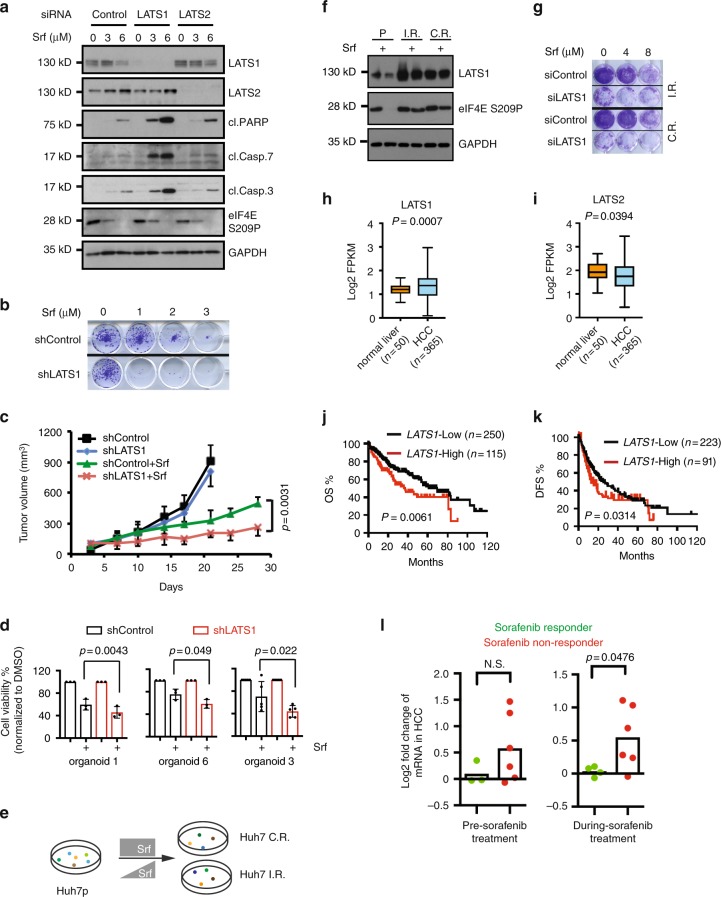
A pro-survival role of LATS1 in HCC cells in response to sorafenib treatment. **a** Huh7 cells were transfected with indicated siControl and treated with DMSO vehicle or sorafenib (Srf) for 72 h. Cell death was analyzed by immunoblotting with indicated antibodies. Results represent three independent experiments. **b** Colony formation was determined in Huh7 cells expressing either shControl (shRNA targeting LacZ) or a shRNA against LATS1 and exposed to sorafenib. Results represent three independent experiments. **c** Huh7 cells either expressing shControlor sh LATS1 were implanted into the flank of NSG mice. The mice were treated with placebo or sorafenib and tumor volumes were measured. *n* = 7–9 for each treatment cohort. Statistical significance was calculated using the R package compareGrowthCurves. **d** Loss of LATS1 expression results in impaired viability of HCC organoid lines in response to sorafenib (Srf). Results were pooled from three to five independent experiments. Statistical significance was calculated using two-tailed, paired *t*-test. **e** Schematic illustration of establishment of sorafenib-resistant cells by (1) step-wise increasing sorafenib concentration (Huh7-IR cells) and (2) consistent high concentration (Huh7-CR cells). **f** Analysis of LATS1 protein expression in sorafenib-resistant cells. Parental (Huh7p) and IR/CR cells were treated with Sorafenib (5 μM) for24 h. Cells were analyzed by immunoblotting with indicated antibodies. Results represent three independent experiments. **g** siRNA-mediated loss of LATS1 expression results in impaired colony formation of sorafenib-resistant Huh7 cells in response to sorafenib (Srf). Results represent three independent experiments. **h**, **i** The expression of LATS1 (**h**) and LATS2 (**i**) was determined in the TCGA database. Expression values were log2-transformed. Statistical analysis was calculated using two-tailed, unpaired Welch’s *t*-test. **j**, **k** Kaplan–Meier analysis of the TCGA database for the expression of LATS1. Overall survival (OS) (**j**) and disease-free survival (DFS) (**k**) of high and low patients are shown. Statistical significance was calculated using log-rank (Mantel–Cox) test. **l** LATS1 expression was determined by RNA sequencing of needle biopsies from patients before sorafenib treatment and during sorafenib treatment. mRNA levels of tumor biopsies were normalized to corresponding adjacent non-neoplastic tissue for each individual patient. Each dot represents one patient (Pre-sorafenib: *n* = 3 for responders and *n* = 6 for non-responders; On-sorafenib: *n* = 4 for responders and *n* = 6 for non-responders). Data represents log2 fold changes. Statistical significance was determined by Komogorov–Smirnov *t*-test.

The mammalian Hippo pathway has been linked to tumorigenesis and therapy resistance^15–17^. While examining the role of the Hippo signaling pathway in the response of HCC cells to Srf treatment, we discovered an unexpected cell survival role of LATS1. Small interfering RNA (siRNA)-mediated ablation of LATS1, but not of LATS2, resulted in an increase of Srf-induced apoptotic cell death and a statistically significant reduction of viability in different HCC cell lines (Fig. 1a and Supplementary Fig. 1a, b), indicating a potential functional diversity between LATS1 and LATS2 in HCC. The pro-survival function of LATS1 in Srf-treated HCC cells was further validated by stable short hairpin RNA (shRNA)-mediated depletion of LATS1 expression (Fig. 1b and Supplementary Fig. 1c–e). Moreover, although shRNA-mediated loss of LATS1 did not affect primary tumor growth of HCC cells in xenotransplanted immunodeficient mice, it significantly reduced tumor growth under Srf treatment (Fig. 1c and Supplementary Fig. 1f). Furthermore, we confirmed a pro-survival role of LATS1 in HCC patient-derived ex vivo organoid lines^18^ (Fig. 1d).

To further examine the role of LATS1 in Srf resistance in HCC, we established Srf-resistant Huh7 cells by (1) step-wise increasing the concentration of Srf to induce resistance (Huh7-IR cells) or (2) by adding a consistent high concentration of Srf to induce resistance (Huh7-CR cells) (Fig. 1e and Supplementary Fig. 1g, h). Analysis of LATS1 protein expression revealed an upregulation in these resistant cells in comparation to the parental line (Fig. 1f). More importantly, knockdown of the upregulated LATS1 in resistant cells led to a reduction of cell viability with decreased number of cell colonies (Fig. 1g).

We next investigated the clinical significance of these observations. Expression of the LATS1 and LATS2 tumor suppressors has been reported to be deregulated in several types of cancers^19^. We first analyzed the expression of LATS1/2 in HCC patient samples in comparison with healthy liver tissues in The Cancer Genome Atlas (TCGA) database^20^. Surprisingly, LATS1 messenger RNA levels were found to be significantly higher, whereas LATS2 mRNA levels were rather lower in HCC patient samples as compared with normal liver controls (Fig. 1h, i). Prognostic analysis of the HCC patient data in the TCGA liver HCC database further supported the notion of a pro-tumorigenic role of LATS1; high levels of LATS1 mRNA correlated with poor overall and disease-free HCC patient survival (Fig. 1j, k). Strikingly, the opposite trend was observed with LATS2, where high LATS2 mRNA levels correlated with better survival in HCC patients, although with marginal significance (Supplementary Fig. 1i, j). Finally, the correlation between LATS1 mRNA expression and Srf response was explored in a local patient cohort where serial and paired tumor and non-tumor biopsies were taken and analyzed before and during Srf therapy. Although not with significance, a trend of higher LATS1 mRNA expression was found in pre-treatment tumor tissues in patients not responding to Srf therapy as compared with Srf therapy responders (Fig. 1l, left panel). Most important, significantly higher LATS1 mRNA expression was found in Srf non-responders as compared with Srf responders after Srf treatment (Fig. 1l, right panel), suggesting that LATS1 mRNA levels may represent a new biomarker to predict Srf response in HCC patients.

Together, these findings demonstrate a role of LATS1, but not LATS2, in therapy response of HCC. Notably, they identify an unexpected HCC cell survival function of the tumor suppressor kinase LATS1 in response to Srf treatment.

### Kinase activity-independent regulation of autophagy by LATS1

LATS1 and LATS2 kinases are known to redundantly mediate the phosphorylation-induced inactivation of the transcription factors YAP/TAZ^21^. Our results indicate a functional diversity between LATS1 and LATS2 in regulating Srf resistance. We thus assessed whether LATS1’s canonical kinase activity was required for its role in cell viability. Analysis of partial resistant HCC cells^22^ forced to express either a wild-type (WT) or a kinase-dead (KD) version of LATS1 revealed a reduction of cell death in cells overexpressing both forms of LATS1 (Supplementary Fig. 1k). Moreover, a similar effect was observed with siRNA-resistant cDNAs mediating the re-expression of a WT or a KD version of LATS1 in LATS1-knockdown cells (Supplementary Fig. 1l, m). Analysis of the activity status of the Hippo transducer YAP confirmed RNA interference (RNAi)-mediated ablation of LATS1 and/or LATS2 resulted into an increase of total YAP protein levels and potentially YAP activity, as monitored by immunoblotting and phos-tag gel electrophoresis (Supplementary Fig. 1n). Together, these results indicate that the pro-survival role of LATS1 is independent of its kinase activity.

To uncover the molecular mechanism underlying the pro-survival function of LATS1 in HCC, we performed transcriptomic analysis by RNA sequencing (RNA-seq) of the HCC cell line Huh7 in response to Srf and siRNA-mediated ablation of LATS1 or LATS2 expression. In line with a specific role of LATS1 in regulating Srf-induced cell death, LATS1 deficiency led to a dramatic change in global transcription as compared to the loss of LATS2 (Supplementary Fig. 2a). Pathways analysis identified phagosomal pathways highly expressed in LATS1-deficient cells (Supplementary Fig. 2b). Gene set enrichment analysis (GSEA) revealed that the changes in gene expression caused by the loss of LATS1 correlated positively with the regulation of autophagy in the Gene Ontology (GO) and Kyoto Encyclopedia of Genes and Genomes (KEGG) databases (Supplementary Fig. 2c), and also in a dataset derived from *Lats1/2*-deficient murine hepatoblasts (Supplementary Fig. 2d).

**Fig. 2:**
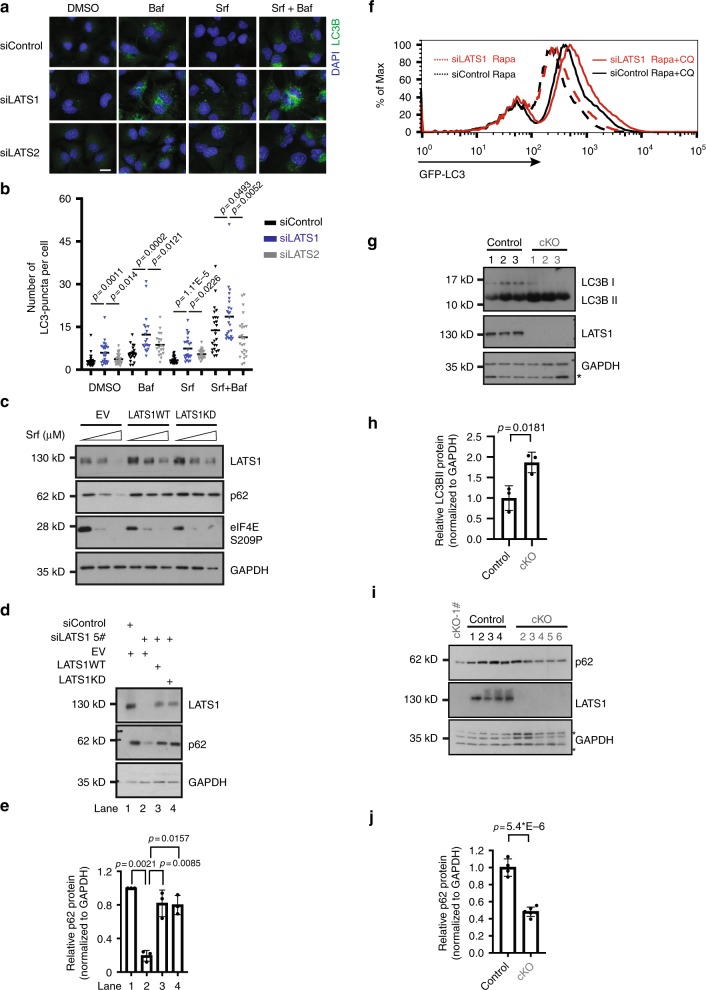
LATS1 restricts autophagy in a kinase activity-independent manner. **a**, **b** Immunofluorescence analysis of LC3B puncta in Huh7 cells in response to sorafenib (Srf) treatment. Cells transfected with indicated siRNAs were treated with DMSO or sorafenib (6 μM for Huh7) for 40 h and bafilomycin A1 (Baf; 0.1 μM) for 2 h. Representative images (**a**) and quantification of LC3B puncta numbers (**b**) from three independent experiments are shown. Statistical analysis was calculated by one-way ANOVA. Scale bars, 25 μm. **c** The forced expression of both wild-type (WT) LATS1 and a kinase-dead (KD D846A) version of LATS1 blocks sorafenib-induced autophagic flux in Hep3B cells. Results represent three independent experiments. **d**, **e** Huh7 cells transfected with either siControl or siRNA against LATS1 were in addition transfected with empty vector (EV) or a vector encoding siRNA-refractory wild-type (WT) or a kinase-dead (KD D846A) version of LATS1, as indicated. Representative immunoblots (**d**) and quantification of the relative p62 levels (**e**) (normalized to GAPDH, fold change) from three independent experiments are shown. Statistical significance was calculated using one-way ANOVA. **f** Flow cytometry analysis of autophagy induction in U2OS-GFP-LC3 cells. Cells were transfected with indicated siRNAs and then treated with rapamycin (100 nM) for 16 h and additional chloroquine (10 μM) for 2 h. Results represent for three independent experiments. **g**, **h** Control mice (*Lats1*^*fl/fl*^; Control) or mice with a hepatocyte-specific depletion of Lats1 (*Alb-Cre;Lats1*^*fl/fl*^; cKO) were treated with chloroquine (30 mg/kg) for 4 h. Liver samples were collected and lysed for immunoblotting analysis. An immunoblot (**g**) and quantification of the relative LC3BII levels (**h**) (normalized to GAPDH, fold change) from *n* = 3 mice per cohort are shown. Statistical significance was calculated using two tailed, unpaired *t*-test. **i**, **j** Control mice (*Lats1*^*fl/fl*^; Control) or mice with a hepatocyte-specific depletion of Lats1 (*Alb-Cre;Lats1*^*fl/fl*^; cKO) were treated with rapamycin (2 mg/kg, twice within 24 h) followed with being fasted for 12 h. Liver samples were collected and lysed for immunoblotting analysis. An immunoblot (**i**) and quantification of the relative p62 levels (**j**) (normalized to GAPDH, fold change) in livers of *n* = 4 for control and *n* = 6 for cKO mice. Statistical significance was calculated using two-tailed unpaired *t*-test.

Srf is well-known to promote autophagy induction and autophagic flux^9^. Thus, we investigated the effect of LATS1 on autophagy induction in HCC cells upon Srf treatment. Indeed, siRNA-mediated knockdown of LATS1, but not LATS2, resulted in a significant increase of LC3B puncta at both basal level and upon Srf stimulation in HCC cells (Fig. 2a, b and Supplementary Fig. 3a, b). Increased LC3B puncta could result from either enhanced autophagy induction or impaired autophagic flux. To discriminate between these possibilities, we first quantified the LC3B puncta number upon treatment with the lysosomal inhibitor bafilomycin A1 (Baf) and with Srf. LC3B puncta numbers were found higher in LATS1-deficient, but not in LATS2-deficient cells, as compared with siControl-transfected cells (Fig. 2a, b and Supplementary Fig. 3a, b), indicating that the loss of LATS1 efficiently promoted autophagy induction.

Furthermore, we analyzed the effect of LATS1 in autophagic flux. Immunoblotting for p62, also known as SQSTM1 and a marker for autophagic flux, revealed a significant reduction of its levels upon loss of LATS1 but not LATS2, indicating that the specific loss of LATS1 also induced a higher autophagic flux activity (Supplementary Fig. 3c). Blocking autophagic flux by chloroquine (CQ) prevented the reduction of p62 upon LATS1 deficiency, indicating that LATS1-mediated p62 downregulation occurred via the activation of autophagic flux (Supplementary Fig. 3d). Conversely, the forced expression of LATS1 inhibited Srf-induced degradation of p62, suggesting a restrictive role of LATS1 in autophagic flux (Fig. 2c and Supplementary Fig. 3e). These results suggest that LATS1, but not LATS2, represses autophagy induction and autophagic flux in HCC cells at baseline and in response to Srf treatment.

Given the notable difference between the functional contribution of LATS1 and LATS2 to autophagy regulation described above, we addressed whether the functional contribution of LATS1 to autophagy regulation depended on its kinase activity. Strikingly, overexpression of both WT and a KD version of LATS1 inhibited Srf-induced p62 degradation, indicating a kinase activity-independent role of LATS1 in autophagy regulation (Fig. 2c). Likewise, exogenously expressed siRNA-refractory WT and KD LATS1 prevented p62 degradation induced by the loss of endogenous LATS1 expression (Fig. 2d, e). Together, the data indicate that LATS1, but not LATS2, represses autophagy in HCC cells by a kinase-independent mechanism.

We next assessed whether the LATS1-mediated prevention of Srf-induced autophagy in HCC cells was specific for Srf treatment or represents a generic effect in response to other molecularly targeted therapies. Indeed, we observed a similar effect of LATS1 on regulation of autophagy and cell death in response to cabozantinib and sunitinib, where knockdown of LATS1 resulted in higher autophagic flux as monitored by p62 degradation and increased cell death (Supplementary Fig. 3f). As we observed a general trend of downregulation of endogenous LATS1 in response to targeted therapies in HCC (Figs. 1a and 2c, and Supplementary Fig. 3e, f), we further investigated potential degradation pathways of LATS1 under these stresses. Interestingly, blockade of autophagy as well as the proteasomal degradation pathway stabilized LATS1 protein, suggesting that Srf treatment can induce autophagic and proteasomal degradation of LATS1 (Supplementary Fig. 3g), and highlighting a potential feedback loop between LATS1 and autophagy regulation.

Next, we determined the direct impact of enhanced autophagy on reduced cell viability in the context of loss of LATS1 expression. We first tested whether the genetic inhibition of autophagy could overcome the enhanced cell death promoted by the loss of LATS1 expression. Repression of autophagy by siRNA-mediated ablation of ATG5 expression, a factor essential for autophagy initiation^23^ (Supplementary Fig. 4a), efficiently prevented cell death and decreased cell viability induced by siRNA or shRNA-induced ablation of LATS1 expression and Srf exposure (Supplementary Fig. 4b–d). Next, we pharmacologically blocked autophagy by CQ and found that CQ could partially revert the reduced colony formation upon loss of LATS1, supporting a death-promoting role of LATS1 deficiency-induced autophagy in response to Srf (Supplementary Fig. 4e, f).

In addition to many tyrosine kinase receptors, Srf also targets RAF kinases. Given a direct LATS1-RAF cross-talk^24^ and a potential crucial role of the mitogen-activated protein kinase (MAPK) signaling pathway in autophagy^25^, we further examined the role of RAF-MAPK signaling in LATS1-mediated Srf response using the MEK inhibitor PD0325901. Although MEK inhibition alone did not have a significant impact on autophagy and cell death, its combination with Srf resulted in increased autophagy and cell death upon RNAi-mediated ablation of LATS1 expression (Supplementary Fig. 4g).

Together, the results demonstrate a kinase activity-independent role of LATS1, but not LATS2, in restricting autophagy and consequently preventing Srf-induced cell death in HCC cells.

### A general role of LATS1-mediated inhibition of autophagy

Increased autophagy has been broadly implicated in cancer therapy, especially in targeted therapy^6^. To assess whether LATS1-mediated autophagy repression exerts a general function in various types of cancer therapy, we analyzed vemurafenib-mediated BRAF inhibition (BRAFi) in BRAFV600E-mutant A2058 melanoma cells. Interestingly, similar patterns of increased LC3B puncta were observed in response to BRAFi in LATS1, but not LATS2, knockdown cells, which were further enhanced upon CQ treatment (Supplementary Fig. 5a, b), suggesting that BRAFi-induced autophagy induction was increased upon loss of LATS1. Analysis of autophagic flux indicated that the loss of LATS1 enhanced vemurafenib-induced p62 degradation (Supplementary Fig. 5c). Increased p62 degradation upon loss of LATS1 could be prevented by the autophagic flux inhibitor CQ, confirming an enhanced autophagic flux (Supplementary Fig. 5d). These results show that LATS1 can also restrict BRAF inhibitor-induced autophagy in melanoma cells.

Autophagy is a cellular response to many stresses and is best characterized under conditions of mammalian target of rapamycin inhibition^26^. We thus assessed the functional role of LATS1 in rapamycin-induced autophagy in standard cellular models, such as U2OS sarcoma cells. Indeed, immunofluorescence microscopy analysis revealed an increased number of LC3B puncta upon LATS1 knockdown in U2OS cells, which was further increased upon CQ treatment (Supplementary Fig. 5e, f), indicating rapamycin-induced autophagy induction was attenuated by LATS1. The analysis of global autophagy induction by flow cytometry analysis of LC3 signal intensity^27^ revealed an increase of LC3 upon LATS1 knockdown, confirming a specific restrictive role of LATS1, but not LATS2, in rapamycin-induced autophagy (Fig. 2f and Supplementary Fig. 5g). In line with decreased p62 protein levels upon rapamycin treatment in LATS1-knockdown cells, cleavage of a green fluorescent protein (GFP)-fused form of LC3, a frequently used alternative assay for autophagic flux activity^28,29^, was also increased upon loss of LATS1, which further supported the notion that LATS1 restricted autophagic flux (Supplementary Fig. 5h). To further validate LATS1-mediated autophagy regulation, we assessed the autophagosome and autolysosome dynamics in U2OS cells stably expressing a fusion RPF-GFP-LC3 construct. Analysis of the numbers of autophagosome (yellow) and of autolysosome (red) in response to rapamycin or CQ or a combination of both confirmed that LATS1 restricted rapamycin-induced autophagy induction as well as autophagic flux activity (Supplementary Fig. 6a–c).

We next investigated the autophagy regulatory role of LATS1 in vivo by comparing liver-specific *Lats1* conditional knockout mice (*Lats*^*fl/fl*^; *Alb-cre*; cKO) to control mice (*Lats*^*fl/fl*^; Control). To investigate the role of Lats1 in autophagy induction of hepatocytes, we treated the mice with CQ and found that loss of Lats1 resulted in higher lipidated LC3BII (Fig. 2g, h), indicating that Lats1 efficiently inhibited autophagy induction in vivo. To study the role of Lats1 in autophagic flux, we treated the mice with rapamycin followed by overnight starvation. Immunoblotting for p62 in the livers of the different genotype mice revealed lower amounts of p62 in cKO mice compared to Control mice (Fig. 2i, j), suggesting that Lats1 also inhibited autophagic flux in vivo.

Together, the observations demonstrate a general inhibitory role of LATS1 in autophagy regulation, either under physiological conditions or in response to targeted therapy or rapamycin-induced cell stress.

### LATS1 interacts with and stabilizes Beclin-1

**Fig. 3:**
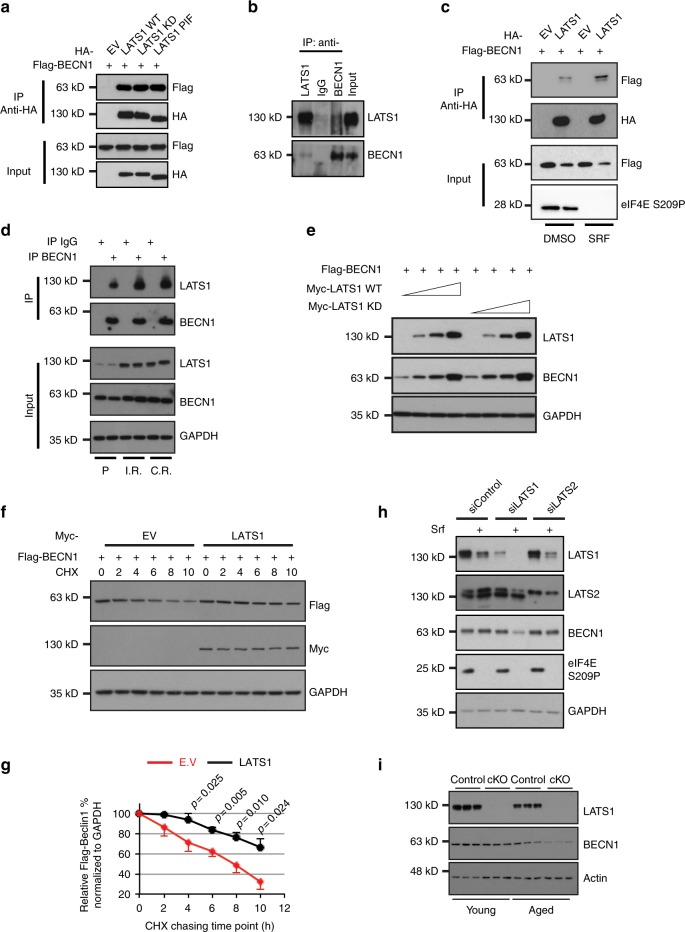
LATS1 interacts with and stabilizes Beclin-1 in a kinase activity-independent manner. **a** HEK293T/17 cells were transfected with indicated plasmids. Seventy-two hours later, cell lysates were immunoprecipitated with anti-HA antibody followed by immunoblotting with indicated antibodies. Input represents lysates directly immunoblotted without immunoprecipitation. Results represent three independent experiments. **b** Huh7 cells were lysed for immunoprecipitation with indicated antibodies followed by immunoblotting analysis for Beclin-1 and LATS1. Input represents lysates before immunoprecipitation. Results represent three independent experiments. **c** HEK293T/17 cells transfected with indicated plasmids were treated with sorafenib (SRF; 10 μM, 2 h) followed by immunoprecipitation with indicated antibody. Results represent three independent experiments. **d** Huh7p or IR or CR cells were lysed for immunoprecipitation with indicated antibodies followed by immunoblotting analysis for Beclin-1 and LATS1. Input represents lysates before immunoprecipitation. Results represent three independent experiments. **e** LATS1 promotes Beclin-1 stability in a dosage-dependent manner. HEK293T/17 cells were transfected with same amount of vector encoding for Flag-tagged Beclin-1 together with increased amounts of vectors encoding for Myc-tagged wild-type LATS1 or kinase-dead mutant LATS1 (KD D846A) as indicated. Seventy-two hours later, cells were lysed and analyzed by immunoblotting for the expression of Beclin-1 (Flag) and LATS1 (Myc). Results represent three independent experiments. **f**, **g** HEK293T/17 cells transfected with indicated plasmids were treated with cycloheximide (CHX; 40 μg/mL) for the indicated times. Representative Immunoblots was shown in **f** and quantification **g** of band intensity was pooled from three independent experiments. Statistical significance was done using one-way ANOVA. **P < 0.01. Note: to achieve equal amount of Beclin-1 protein level at time point 0, amounts of Flag-Beclin-1 plasmid transfected between empty vector and LATS1 were 3:1. **h** Huh7 cells transfected with indicated siRNAs were treated with DMSO or sorafenib (Srf; 10 μM) for 48 h. Cell lysates were analyzed by immunoblotting with indicated antibodies. A representative immunoblot from three independent experiments is shown. **i** Liver tissues from Control mice (*Lats1*^*fl/fl*^; Control; *n* = 3) or mice with a hepatocyte-specific depletion of Lats1 (*Alb-Cre;Lats1*^*fl/fl*^; cKO; *n* = 3) were collected at young adult age (12–16 weeks) or at old age (55–65 weeks) and lysed for immunoblotting analysis.

To delineate the molecular mechanisms underlying autophagy regulation by LATS1, we assessed whether LATS1 directly or indirectly affected any autophagy core-machinery proteins. Beclin-1 exerts a central role in autophagy induction and execution by orchestrating different autophagy complexes via direct binding to a variety of co-factors, but also by forming inactive homo-dimers^30^. Given that the LATS family kinase NDR can interact with Beclin-1^31^, we first tested a potential physical interaction between LATS1 and Beclin-1. Strikingly, LATS1 formed a complex with Beclin-1 in a kinase activity-independent manner, as both KD and constitutively active (PIF) versions of LATS1 interacted with Beclin-1 to a comparable extent as WT LATS1 (Fig. 3a). By comparing LATS1 and NDR1 on Beclin-1 interaction and Srf-induced autophagy, we found that LAST1 interacted with Beclin-1 with much higher affinity (Supplementary Fig. 7a). Although RNAi-mediated ablation of LATS1 increased Srf-induced autophagic cell death, loss of NDR1 barely had any effect Srf-induced autophagy (Supplementary Fig. 7b). The LATS1-Beclin-1 protein interaction was also observed with endogenous proteins (Fig. 3b). Importantly, an increased interaction between LATS1 and Beclin-1 was observed in response to Srf treatment, as well as in Srf-resistant cells, indicating a potential Srf stress-induced specific LATS1-Beclin-1 interaction (Fig. 3c, d). Notably, the LATS1-Beclin-1 interaction substantially promoted an upregulation of Beclin-1 protein in a LATS1 dosage-dependent manner (Fig. 3e). In striking contrast, LATS2 barely had any effect on Beclin-1 protein stability (Supplementary Fig. 7c). Various versions of kinase-inactive LATS1 as well as the constitutively active LATS1 PIF-mutant stabilized Beclin-1 comparable to WT LATS1 (Supplementary Fig. 7d). The use of different protein tags, either at the C- or N-terminal end of LATS1, confirmed the impact of LATS1 on Beclin-1 stabilization (Supplementary Fig. 7e).

We next determined whether LATS1 promoted Beclin-1 upregulation at the mRNA or protein level. Analysis of Beclin-1 mRNA revealed no difference between control and LATS1-knockdown cells (Supplementary Fig. 7f), yet a cycloheximide pulse-chase experiment revealed that overexpressed LATS1 stabilized Beclin-1 at the protein level (Fig. 3f, g). This stabilizing effect was further confirmed at the endogenous level, where ablation of LATS1, but not LATS2, resulted in decreased Beclin-1 protein levels in response to the cap-dependent protein synthesis blockade induced by Srf (Fig. 3h). Furthermore, aging-induced downregulation of Beclin-1 was further enhanced upon Lats1 deficiency in mouse livers in vivo, indicating a specific Lats1-mediated Beclin-1 stabilization during aging (Fig. 3i). These findings suggested that LATS1 stabilized Beclin-1 at the protein level.

Taken together, these results indicate that LATS1 interacts with and stabilizes Beclin-1 protein.

### Identification of the functional diversity between LATS1 and LATS2

**Fig. 4:**
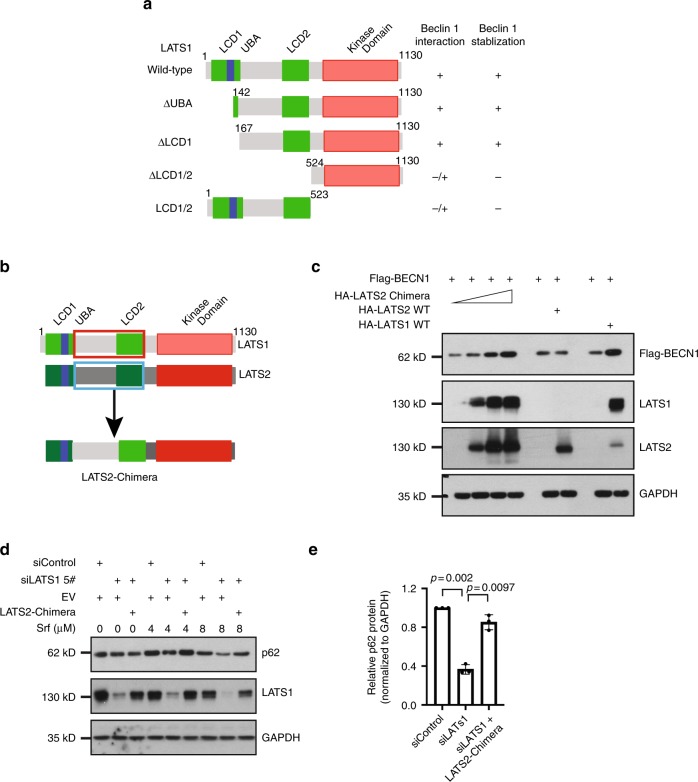
Functional characterization of the divergence between LATS1 and LATS2. **a** Schematic representation of the protein domain structure and truncated mutants of LATS1 used for mapping the LATS1 protein domain required for Beclin-1 interaction and stabilization. **b** Schematic illustration of the generation of a LATS2-LATS1 chimeric protein by replacing the center domain of LATS2 (blue bracket) with the analogous domain of LATS1 (red bracket; amino acids 151–554) inserted between the wild-type LATS2 N-terminal (amino acids 1–147) and C-terminal (amino acids 514–1088) domains. **c** Functional validation of the LATS2 chimera protein in Beclin-1 stabilization. HEK293T/17 cells were transfected with the same amount of vectors encoding for Flag-tagged Beclin-1 (Flag-BECN1) together with HA-tagged wild-type LATS1 or LATS2, or increasing amounts of vector encoding the HA-tagged LATS2-chimeric protein as indicated. Cells were collected 72 h after transfection and analyzed by immunoblotting for Flag-BECN1, LATS1, and LATS2. Immunoblotting for GAPDH was used as loading control. Results represent three independent experiments. **d**, **e** LATS2 chimera protein can fully rescue autophagic flux (p62) in endogenous LATS1-knockdown Huh7 cells in response to sorafenib. Huh7 cells transfected with siControl or siLATS1 were in addition transfected with empty vector (EV) or a vector encoding for LATS2 chimera. Cells were lysed 72 h after transfection and p62 and LATS1 protein levels were analyzed by immunoblotting. GAPDH was used as loading control. A representative immunoblot (**d**) and quantification of the relative p62 levels (**e**) (sorafenib 8 μM condition, normalized to GAPDH) from three independent experiments are shown. Statistical significance was calculated using one-way ANOVA.

Our findings revealed a surprising functional diversity between LATS1 and LATS2 in regulating autophagy and Beclin-1 protein stability. We thus sought to identify the molecular difference underlying these divergent functions of LATS1 and LATS2. Using the stabilization of Beclin-1 by interacting with LATS1 but not LATS2, we mapped the essential domain of LATS1 required for interacting with and stabilizing Beclin-1 between amino acids 167 and 523 (Fig. 4a and Supplementary Fig. 7g, h). Of note, sequence alignment between LATS1 and LATS2 showed that a major part of this domain represented the most diverse amino acid sequences between LATS1 and LATS2 (amino acids 151–554 in LATS1 and amino acids 149–513 in LATS2, respectively). In contrast, the LATS1 kinase domain did not seem essential. This notion was functionally confirmed by using a construct containing only the LATS1 kinase domain but not the essential domain; the kinase domain alone failed to repress Srf-induced autophagy (Supplementary Fig. 7i).

We examined the importance of the essential domain by replacing this variable area of LATS2 with the comparable area from LATS1, generating a LATS2 chimera protein, which contained unchanged domains of LATS2 at the N and C termini but with a middle domain derived from LATS1 (Fig. 4b). Interestingly, the LATS2 chimera stabilized Beclin-1 to a comparable extent as WT LATS1, whereas WT LATS2 had no effect (Fig. 4c). The LATS2-chimeric protein could also restore the p62 levels that were reduced by the siRNA-mediated ablation of LATS1 expression (Fig. 4d, e).

These data show that a small protein domain present in LATS1 but not in LATS2 is sufficient to mediate the stabilization of Beclin-1 and, thus, to repress autophagy. Since current knowledge suggests a redundant function of LATS1 and LATS2, these results are unexpected and identify a diverse function of the two closely related kinases at the molecular level.

### LATS1 promotes K27-ubiquitination of Beclin-1 on lysines K32 and K263

Stabilization of proteins is frequently triggered by their post-translational modification, for example by ubiquitination. Beclin-1 is tightly regulated at the post-translational level by phosphorylation, acetylation and ubiquitination^30,32–34^. Ubiquitination of Beclin-1 generally leads either to its proteasomal degradation or stabilization or to its activation, depending on the specific ubiquitin linkage^35^.

**Fig. 5:**
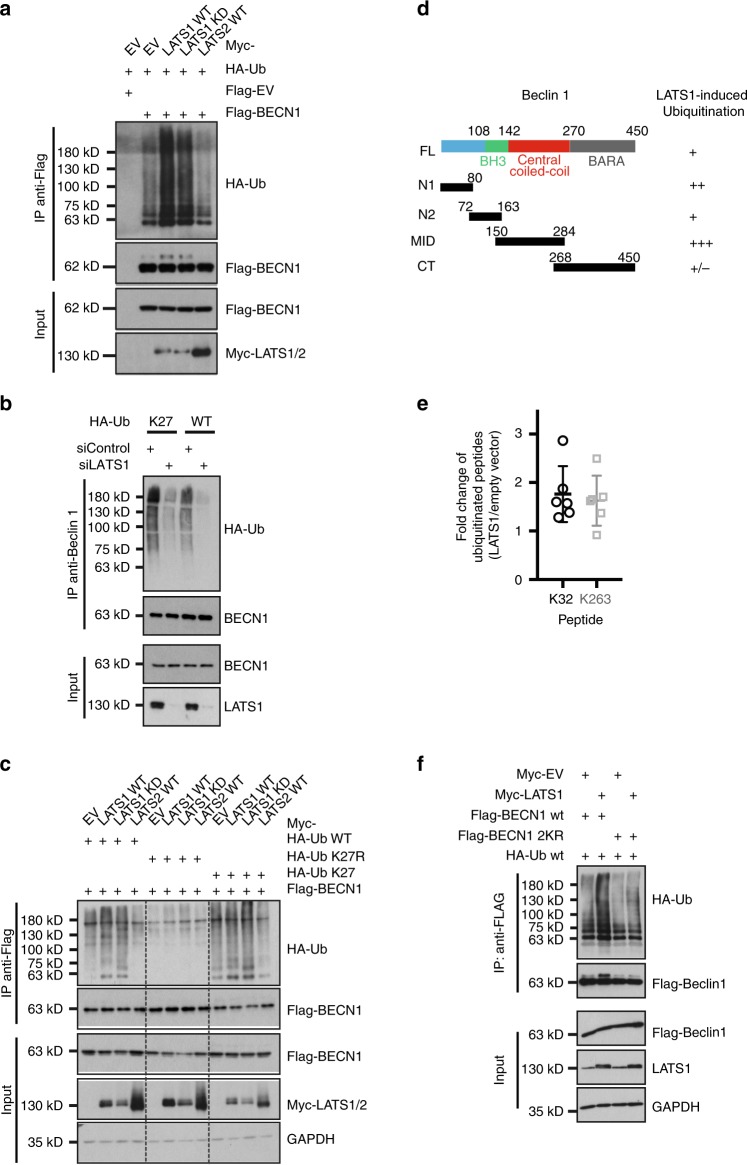
LATS1 promotes Beclin-1 ubiquitination at lysine residues K32 and K263. **a** HEK293T/17 cells transfected with indicated plasmids were collected 72 h later for immunoprecipitation with anti-Flag antibody and then immunoblotted for HA and Flag-tagged proteins and for LATS1/2. Input represents analysis of cell lysates before immunoprecipitation. Immunoblots represents three independent experiments. **b** HEK293T/17 cells were transfected with indicated siRNAs followed by transfection with HA-tagged wild-type or K27 (all lysins were mutated to arginine except K27) ubiquitin constructs. Seventy-two hours later, cell lysates were prepared and immunoprecipitated with Beclin-1 antibody and immunoblotted with antibodies against Beclin-1 and HA (HA-Ub). Input represents immunoblotting of cell lysates for Beclin-1 and LATS1 before immunoprecipitation. Immunoblots represent three independent experiments. **c** HEK293T/17 cells transfected with indicated plasmids. Seventy-two hours later, cell lysates were prepared and immunoprecipitated with anti-Flag antibody and immunoblotted with antibodies against HA and Flag-tagged proteins and LATS1/2. Input represents analysis of cell lysates before immunoprecipitation. GAPDH was used as loading control. Immunoblots represent three independent experiments. **d** Schematic representation of LATS1-mediated ubiquitination levels in the different domains of Beclin-1. Note that the MID construct containing the central coiled-coil domain is the minimal recipient domain for ubiquitination. **e** Mass spectrometric identification of ubiquitination sites in Beclin-1 induced by LATS1. HEK293T/17 cells were transfected with vectors encoding for HA-tagged wild-type ubiquitin, Flag-tagged-Beclin-1 with vectors encoding for empty vector and Myc-tagged LATS1. After immunoprecipitation with antibodies against Flag, samples were eluted for ubiquitination site identification by mass spectrometry. Related fold change of ubiquitinated peptide (ratio of LATS1/empty vector) is presented. Results were pooled from five independent experiments. **f** Mutation of lysine residues K32/263 blocks LATS1-induced ubiquitination of Beclin-1. Beclin-1 lysine K32 and K263 were mutated to arginine via site-directed mutagenesis. HEK293T/17 cells were transfected with indicated plasmids. Cells were collected 72 h later for immunoprecipitation with anti-Flag antibody and then immunoblotted for HA, Flag tagged proteins and LATS1/2. Input represents analysis of cell lysates before immunoprecipitation. Immunoblots represents three independent experiments. Note that the different proteins have been analyzed on different membranes.

Thus, we assessed whether LATS1 had any effect on Beclin-1 ubiquitination. Surprisingly, although LATS1 stabilized Beclin-1 protein, we found that LATS1, but not LATS2, substantially promoted Beclin-1 ubiquitination, notably without requiring its kinase activity (Fig. 5a). Furthermore, endogenous LATS1 also promoted Beclin-1 ubiquitination as knockdown of LATS1 resulted in decreased ubiquitination of endogenous Beclin-1 (Fig. 5b). Consistent with its ability to stabilize Beclin-1, the hybrid LATS2 chimera potently increased ubiquitination of Beclin-1 (Supplementary Fig. 8a). The use of a large number of lysine mutant versions of ubiquitin revealed that both exogenous and endogenous LATS1-induced ubiquitination of Beclin-1 involved a lysine 27-mediated linkage of ubiquitin molecules (Fig. 5b, c and Supplementary Fig. 8b, c, d).

To further delineate the functional role of LATS1-induced Beclin-1 ubiquitination, we first analyzed which protein domain of Beclin-1 was ubiquitinated in a LATS1-dependent manner. Co-expression of various GFP-fused domains of Beclin-1 with LATS1 and ubiquitin identified the N terminus and central coiled-coil domain of Beclin-1 as most susceptible to LATS1-induced ubiquitination (Fig. 5d and Supplementary Fig. 8e). Next, we sought to map the LATS1-induced ubiquitination sites via mass spectrometry (MS) analysis. Indeed, lysine residues 32 and 263, which locate inside the N-terminal intrinsic disordered domain and central coiled-coil domain, respectively, were predominantly detected with increased levels of ubiquitination by LATS1 (Fig. 5e and Supplementary Fig. 8f). Sequence alignment of lysine residues K32 and K263 across different species revealed that K263 was more conserved (Supplementary Fig. 8g), indicating that K263 ubiquitination induced by LATS1 or homolog genes might be a more common event.

To validate whether these two lysine residues were bona fide sites on Beclin-1, we mutated these two lysine (K32/263) residues to arginine (R). Strikingly, while WT Beclin-1 was efficiently ubiquitinated in dependence on LATS1, this ubiquitination was greatly diminished with the K32/263R mutant version of Beclin-1 (Fig. 5f).

K27 linkage represents a largely unexplored, non-canonical form of ubiquitination. The HECT family E3 ligase NEDD4 has been previously reported to mediate K6, K11, and K27-linked polyubiquitination and consequently influence the stability of Beclin-1^36,37^. We thus assessed whether NEDD4 play a role in LATS1-mediated K27-linked ubiquitination on Beclin-1. First, we confirmed that the forced expression of NEDD4 stabilized Beclin-1 protein in a dose-dependent manner (Supplementary Fig. 9a). This NEDD4-mediated stabilization of Beclin-1 was accompanied by its increased K27-linked ubiquitination (Supplementary Fig. 9b). Although LATS2 interacts with Beclin-1 at a visible lower affinity in comparison with LATS1 (Supplementary Fig. 9c), LATS2 did not have any effect on Beclin-1 ubiquitination (Fig. 5a, c) and stability (Supplementary Fig. 7a, b). We next assessed whether NEDD4 interaction can distinguish LATS1 and LATS2. In line with a previous report^36,38^, LATS1 was also found to physically interact with NEDD4 (Supplementary Fig. 9d). Importantly, the interaction between LATS1 and NEDD4 was independent of its kinase activity, but required its unique domain not present in LATS2 (Supplementary Fig. 9d, e). Consequently, LATS1, but not LATS2, promoted NEDD4 stability (Supplementary Fig. 9f, g). Conversely, NEDD4-LATS1 interaction led to the degradation of LATS1 but not LATS2 (Supplementary Fig. 9g, h). To assess whether NEDD4 directly affected LATS1-induced Beclin-1 ubiquitination on K32/263, we analyzed the potential NEDD4-mediated K27-ubiquitination of Beclin-1. Strikingly, while NEDD4 potently induced K27-linked polyubiquitination of WT Beclin-1, it failed to do so in the K32/263R mutant (Supplementary Fig. 9i), identifying NEDD4 as a potential E3 ligase for K27-linked polyubiquitination at K32/263 of Beclin-1.

Together, the results suggest that LATS1 specifically promotes a K27-linked ubiquitination of Beclin-1 at lysine residues 32 and 263, most likely via the E3 ligase NEDD4.

### Beclin-1 K32/263 ubiquitination is required for LATS1 effect

**Fig. 6:**
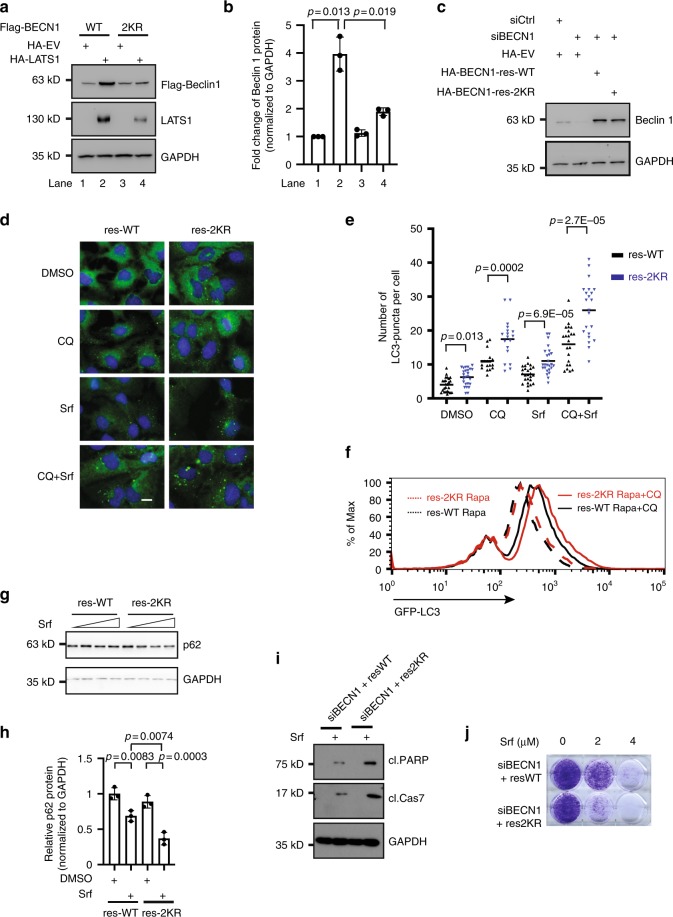
LATS1-induced Beclin-1 ubiquitination is required for Beclin-1 stabilization and autophagy regulation. **a**, **b** Mutation of lysine residues K32/263 in Beclin-1 blocks LATS1-induced Beclin-1 stabilization. HEK293T/17 cells were transfected with the same amount of vector encoding for Flag-tagged wild-type or K32/263R lysine mutant Beclin-1 together with a vector encoding for HA-tagged LATS1 or empty vector as indicated. Seventy-two hours later cells were analyzed by immunoblotting with indicated antibodies. Representative immunoblots (**a**) and quantification of the relative Flag-tagged Beclin-1 (**b**) (normalized to GAPDH) from three independent experiments are shown. Statistical significance was calculated using one-way ANOVA. **c** Huh7 cells were transfected with indicated siRNAs followed by transfection with HA-tagged siRNA-refractory wild-type or K32/263R lysine mutant Beclin-1 cDNAs. Cells were analyzed by immunoblotting with indicated antibodies. Results represent three independent experiments. **d**, **e** Huh7 cells were transfected with indicated siRNAs followed with siRNA-refractory wild-type or K32/263R mutant Beclin-1 cDNAs. Cells were then treated with DMSO or sorafenib (Srf; 6 μM for Huh7) for 24 h and/or in combination with chloroquine (CQ; 20 μM) for 2 h before fixation. Representative images (**d**) and quantification of LC3B-puncta numbers (**e**) pooled from three independent experiments are shown. Statistical analysis was calculated by one-way ANOVA. Scale bars, 25 μm. **f** U2OS-GFP-LC3 Cells were transfected with indicated siRNAs followed with siRNA-refractory wild-type or K32/263R lysine mutant Beclin-1 cDNAs. Cells were then treated with rapamycin (Rapa; 100 nM) for 16 h and/or in combination with chloroquine (CQ; 10 μM) for 2 h before analysis. Results represent three independent experiments. **g**, **h** Huh7 cells were transfected with indicated siRNAs followed with siRNA-refractory wild-type or K32/263R lysine mutant Beclin-1 cDNAs. Cells were then treated with sorafenib for 24 h. Cells were analyzed by immunoblotting. Representative images (**g**) and quantification of relative p62 levels (**h**, treated with DMSO or 6 μM sorafenib) pooled from three independent experiments are shown. Statistical analysis was calculated by one-way ANOVA. Scale bars, 25 μm. **i, j** Cells were transfected with indicated siRNAs followed with siRNA-refractory wild-type or K32/263R mutant Beclin-1 cDNAs. **i** Cells then treated with sorafenib (Srf; 6 μM) for 48 h for immunoblotting (**i**) or for 7–10 days for colony formation assays (**j**). Results represent three independent experiments.

Our observations suggest that LATS1 induces Beclin-1 stabilization and K27-linked ubiquitination at K32/263 of Beclin-1. To determine whether this Beclin-1 ubiquitination affects its protein stability, we directly tested the LATS1-mediated stabilization of WT and mutant Beclin-1. Interestingly, while WT Beclin-1 was potently stabilized, LATS1-mediated stabilization was significantly impaired with the K32/263R mutant (Fig. 6a, b). These results indicate that K27-linked ubiquitination of Beclin-1 on K32/263 is relevant for Beclin-1 stability.

To assess whether LATS1-induced ubiquitination of Beclin-1 had a causal role in autophagy regulation, we re-introduced siRNA-refractory versions of either WT Beclin-1 or its K32/263R mutant into Beclin-1 knockdown cells (Fig. 6c) and assessed autophagy induction by Srf in HCC cells and by rapamycin in U2OS cells. Interestingly, we observed an enhanced autophagy induction, as reflected by an increased number of LC3B puncta upon immunofluorescence staining (Fig. 6d, e) or a higher LC3 signal by flow cytometry (Fig. 6f) in the Beclin-1 K32/263R mutant as compared with WT Beclin-1. Next, we determined autophagic flux in cells expressing either WT or K32/263R mutant Beclin-1 by analyzing p62 protein degradation in response to Srf treatment. In line with an increased autophagy induction, we observed higher autophagic flux (Fig. 6g, h).

Although Beclin-1 generally represented an essential gene for cell viability (Supplementary Fig. 10a), we further analyzed the biological effects of LATS1-medated ubiquitination of Beclin-1. In agreement with a restrictive role in autophagy, we found that ubiquitination deficiency in Beclin-1 resulted in increased cell death and decreased long-term cell viability (Fig. 6i, j).

The results indicate that LATS1-induced ubiquitination of Beclin-1 functionally results in Beclin-1 stabilization and, with it, into a repression of autophagic cell death.

### LATS1 induces inactive Beclin-1 dimerization

**Fig. 7:**
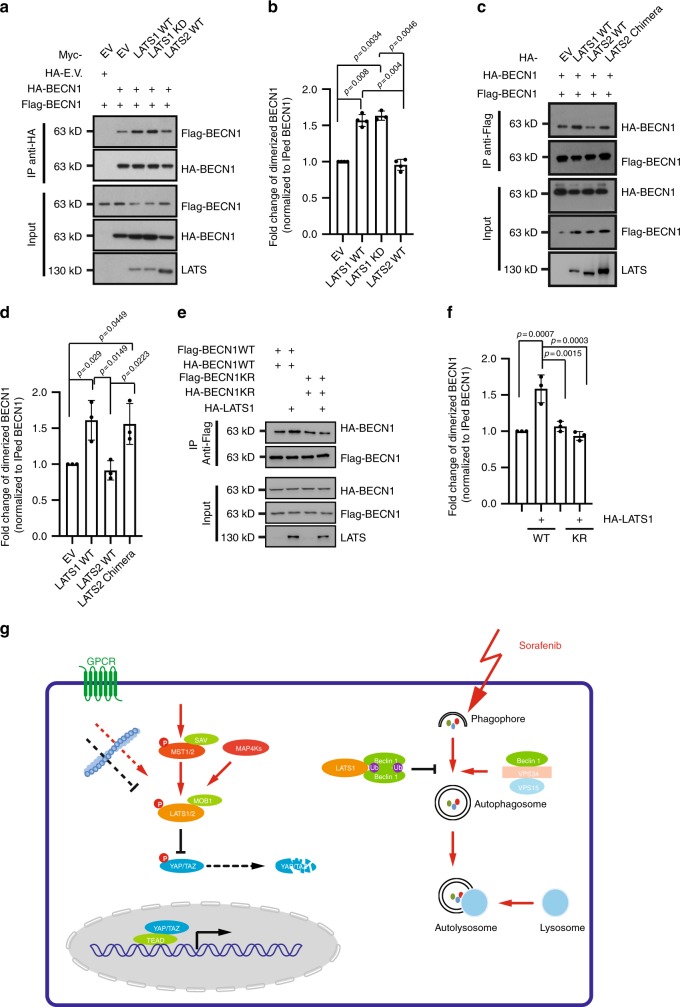
LATS1 induces inactive Beclin-1 self-dimerization. **a** LATS1, but not LATS2, promotes Beclin-1 self-dimerization in a kinase activity-independent manner. HEK293T/17 cells were transfected with indicated plasmids. Cells were collected 72 h later for immunoprecipitation with anti-HA antibody and then immunoblotted for HA, Flag-tagged Beclin-1, and LATS1/2. Input represents analysis of cell lysates before immunoprecipitation. Immunoblots represent three independent experiments. **b** Quantification of dimerized Beclin-1 (BECN1) protein abundancy pooled from four independent experiments described in (**a**). Statistical significance was calculated using one-way ANOVA. ***P < 0.001. **c** LATS2 chimera maintains the ability to promote Beclin-1 self-dimerization. HEK293T/17 cells were transfected with Flag-tagged (Flag-BECN1) and with HA-tagged (HA-BECN1) Beclin-1 and with empty vector (EV) or with vectors encoding for wild-type (WT) LATS1 or LATS2, or LATS2 chimera, as indicated. Cells were collected 72 h later for immunoprecipitation with anti-Flag antibody and then immunoblotted for HA, Flag-tagged Beclin-1, and LATS1/2. Input represents analysis of cell lysates before immunoprecipitation. Immunoblots represent three independent experiments. **d** Quantification of dimerized Beclin-1 (BECN1) protein abundancy pooled from four independent experiments described in **c**. Statistical significance was calculated using one-way ANOVA. *P < 0.05. **e** LATS1-induced ubiquitination at lysine 263 is essential for LATS1-mediated promotion of Beclin-1 self-dimerization. HEK293T/17 cells were transfected with indicated plasmids. Cells were collected 72 h later for immunoprecipitation with anti-Flag antibody and then immunoblotted for HA, Flag-tagged Beclin-1, and LATS1. Input represents analysis of cell lysates before immunoprecipitation. Immunoblots represent three independent experiments. **f** Quantification of dimerized Beclin-1 (BECN1) protein abundancy pooled from three independent experiments described in **e**. Statistical significance was calculated using one-way ANOVA. ***P < 0.001, *P < 0.05. **g** Schematic representation of the role of LATS1 in canonical Hippo signaling (left side) and of its non-canonical role in repressing sorafenib-induced autophagy (right side). LATS1-induced K27-linked ubiquitination of Beclin-1 on K32 and K263 results into its inactivation by self-dimerization and thus to an inhibition of autophagy.

Our results suggest a LATS1-specific role in restricting autophagy via modulating Beclin-1 protein through K27-linkage ubiquitination. K27-linked ubiquitin bestows some unique features, which lead to stabilization and/or dimerization/aggregation of targeted proteins^39–42^. We thus addressed how LATS1-induced Beclin-1 ubiquitination may affect autophagy. Notably, K32 and K263 on Beclin-1, which are ubiquitylated by NEDD4/LATS1, locate within the N-terminal intrinsic disordered domain and coiled-coil domain, respectively (Fig. 4d). Although the intrinsic disordered domain is structurally unexplored, the coiled-coil domain has been reported essential for Beclin-1 to form activating autophagy regulatory complexes as well as inactive Beclin-1 self-dimers^43^. Remarkably, an anti-parallel coiled-coil structure within a Beclin-1 homo-dimer involves most of their coiled-coil domains (aa 175–263), where the C termini of the coiled-coil domains (aa 248–265) are critical for stabilization of the homo-dimer^44^. Crystal structural studies suggest that lysine 263 is the last amino acid of alpha helix^45^. Based on the stabilizing effect of LATS1 on Beclin-1, the potential effect of K27-linked ubiquitination on protein aggregation, and the unique position of K263 within the C terminus of the coiled-coil domain, we assessed whether LATS1 promoted Beclin-1 self-dimerization. Indeed, LATS1 significantly promoted Beclin-1 self-dimer formation in a kinase-independent manner (Fig. 7a, b). Consistent with Beclin-1 stabilization reported above, LATS2 failed, whereas the hybrid LATS2 chimera efficiently induced Beclin-1 self-dimerization (Fig. 7c, d). Finally, LATS1-induced Beclin-1 homo-dimer formation was found reduced with the Beclin-1 K263R mutant (Fig. 7e, f), indicating that ubiquitylation on lysine 263 is functionally essential to mediate the role of LATS1 in promoting Beclin-1 homo-dimer formation and thus in preventing autophagy. Consistent with this notion, LATS1 also repressed the formation of an active autophagy initiation complex (Beclin-1/ATG14L/VPS34) (Supplementary Fig. 10b).

Together, the data demonstrate that LATS1 promotes the formation of inactivated Beclin-1 homo-dimers to prevent it from forming pro-autophagic (Beclin-1/ATG14L/VPS34) complexes. Thus, we report a kinase-independent function for LATS1, but not LATS2, in preventing autophagy under physiological conditions and in response to therapy stress and thus regulating therapy response in HCC cells (Fig. 7g). Such pro-tumorigenic activity is certainly an unexpected function of the well-studied tumor suppressor LATS1, an insight that may open new avenues to overcome therapy resistance.

## Discussion

LATS1 and LATS2 kinases are evolutionarily conserved tumor suppressors, which share ~50% and 85% amino acid sequence identity between the full proteins and the kinase domains, respectively. In most cellular contexts, LATS1 and LATS2 act in a redundant manner as effector kinases of the Hippo signaling pathway by phosphorylating and inactivating the transcription factors YAP and TAZ^21^. We here report a functional divergence between LATS1 and LATS2 by the identification of a kinase activity-independent scaffolding function of LATS1, but not LATS2, in the regulation of autophagy. Although LATS1 promotes cell viability in response to Srf treatment of HCC cells, LATS2 has no effect. Analysis of autophagy modulation by LATS1 and LATS2 further confirms our observation of a specific role of LATS1 in restricting Srf-induced lethal autophagy in HCC cells. Subsequent mechanistic studies reveals a unique role of LATS1, but not LATS2, in stabilizing the autophagy core-machinery protein Beclin-1 via K27-linked ubiquitination and self-dimerization. The distinct role between LATS1 and LATS2 in autophagy regulation is exerted by a protein domain of LATS1 located between amino acids 167 and 524, a domain that also represents the most divergent part between LATS1 and LATS2.

The major function of LATS kinases in Hippo signaling is to phosphorylate the transcription factors YAP and TAZ, which earmarks them for nuclear export, deactivation, and degradation. On the other hand, the kinase activity-independent roles of LATS kinases have just started to be addressed. For instance, LATS2 restricts WNT signaling activity by disrupting the BCL9/β-catenin complex, which is dependent on its N-terminal domain but not on its kinase domain^46^. Likewise, the N-terminal domains of LATS1 and LATS2 are required to target ERα for DCAF-1-mediated degradation during mammary gland lineage commitment^47^. Interestingly, LATS1 is reported to interact with NF2^48^, CRL4^DCAF1^ ^49^, and CDK2^50^. Although the kinase domain is required to interact with CRL4^DCAF1^ (aa 598–1130)^49^, the N-terminal domain of LATS1 has been identified to mediate its interaction with NF2 (AIxEIxxSL, aa 72–89 of LATS1)^48^, CDK20 (aa 1–200 of LATS1)^50^, and Beclin-1 (aa 154–554 of LATS1) (this report). These results indicate different but unique interaction interfaces among these LATS1 complexes. Whether and how conformation changes of LATS1 induced by its interaction with specific binding partner affects the binding to other partners certainly merits further biochemical investigation.

On the other hand, an interplay between the Hippo signaling pathway and autophagy has been identified by mainly focusing on the activity of MST kinases, the upstream regulators of LATS activity. For instance, MST kinases regulate autophagy by directly phosphorylating BCL2, BCL-X_L_ and LC3B proteins^51–53^. LATS1 also interacts with BAG3^54^, a BCL family member implicated in tension-induced autophagy^55^. However, a direct functional contribution of LATS1 to BAG3-mediated autophagy remains open. A role of the LATS1 homolog warts (wts) in autophagy has been described in *Drosophila* and *Caenorhabditis elegans*. Although *wts* mutation leads to attenuated autophagy in the salivary glands of the fly, RNAi-mediated depletion of wts leads to degradation of p62 in the worm^56,57^. These results suggest a a potential cellular context-dependent role of wts in autophagy regulation.

Our study has used an unbiased bioinformatics analysis to identify a restrictive role of LATS1, but not LATS2, in Srf-induced autophagy in HCC and other cancer types and normal liver in vivo. Importantly, this distinct function of LATS1, but not LATS2, in autophagy appears independent of its kinase activity. Our data rather indicate that LATS1 acts as a scaffold to bind Beclin-1 and to promote K27-linked ubiquitination of Beclin-1 at lysine residues K32 and K263 in its N-terminal intrinsic disordered domain and coiled-coil domain, respectively. Consequently, K27-linked ubiquitination of Beclin-1 on K32 and K263 promotes Beclin-1 stabilization, its self-dimerization, and autophagy inhibition. Although increased at the protein level, in its self-dimerized form Beclin-1 is inactivated and can no more contribute to the execution of autophagy^43^, as for instance induced by Srf treatment of HCC cells (Fig. 7g). It is noted that NEDD4 functions as a potential ubiquitin E3 ligase in regulating LATS1-induced ubiquitination of Beclin-1. Sequence alignment across different species of Beclin-1 lysine residue K32 and K263 suggests that K263 might represent evolutionary conserved regulatory mechanism of LATS1/wts towards Beclin-1. Interestingly, invertebrate genomes encode one wts kinase, whereas vertebrate genomes encode two wts homolog kinases (LATS1 and LATS2). Beclin-1, on the other hand, functions as a platform to orchestrate diverse autophagy regulatory complexes^29,30^. Notably, the central coiled-coil domain plays a key role in shifting Beclin-1 to different sub-complexes, such as Beclin-1/UVRAG, Beclin-1/ATG14, or its inactive homo-dimer. In line with previous findings of an important input of the C terminus of the coiled-coil domain to homo-dimer formation^44^, our results demonstrate that lysine residue K263 is critical for Beclin-1 homo-dimer formation, which functionally mediates the regulatory role of LATS1 in autophagy.

In contrast to its tumor suppressive role in the Hippo signaling pathway, we report that LATS1 exerts a pro-survival function in HCC cells in response to Srf treatment, i.e., an oncogenic activity. Indeed, RNAi-mediated ablation of LATS1 expression results in an increase of Srf-induced apoptosis and a reduction of cell viability in vitro and a decrease of tumor growth in vivo. Moreover, gene expression analysis of HCC patient samples indicates a poor survival of patients with high expression of LATS1 in their tumors, further supporting the notion of a pro-oncogenic role of LATS1 in HCC cells. Most importantly, a significant higher expression of LATS1 is observed in patients not responding to Srf as compared with patients responding to Srf therapy, suggesting LATS1 as a clinically relevant biomarker for Srf sensitivity.

The therapeutic targeting of the Hippo signaling pathway is currently under intense investigation in basic and pharmaceutical research laboratories. Here we have identified a non-redundant function of LATS in HCC. We have delineated the mechanistic details of a kinase activity-independent function of LATS1 in autophagy regulation and in tumorigenesis, thus raising a note of caution on the therapeutic targeting of LATS kinases. Their pro-tumorigenic and tumor suppressive roles, their non-redundant distinct activities and their kinase activity-dependent and independent functions need to be considered to efficiently interfere with tumor progression and therapy resistance and to avoid undesired consequences.

## Methods

### DNA plasmids, siRNA, and shRNA sequences and antibodies

DNA plasmids and sequences of siRNAs used in this study are listed in Supplementary Dataset 1. Additional information on antibodies is provided in Supplementary Dataset 2.

### Cell culture, transfection, and reagents

HEK293T/17, Huh7, Hep3B, SNU423, and SNU398 were obtained from American Type Culture Collection. Hep40 cell line was a kind gift from Brian Carr. U2OS cell line stably expressing GFP-LC3 was a kind gift from Carine Joffre. All the cell lines in this study were regularly confirmed for the absence of *Mycoplasma* contamination. Transfection of plasmids into HEK293T/17 and HCC cells were carried out using jetPEI (polyPlus Transfections) and Lipofectamine 3000 (Invitrogen) according to the manufacturers’ instructions, respectively. Lipofectamine RNAiMAX (Invitrogen) was used to transfect siRNAs. To establish stable knockdown or overexpression cell lines, retrovirus were produced by transfection of Platinium-A cells (Cell Biolabs) with pBabe-retro-puro or pSuper-retro-puro constructs, respectively. Control or gene-specific siRNAs were from Dharmacon as ON-TARGET plus SMARTpools or Set of 4 upgrade products.

Srf was from Selleckchem (in vitro study) and LC Laboratories (in vivo study). Baf and CQ were from Sigma.

### HCC patient-derived ex vivo organoid lines

The stable HCC organoid lines used in this study have been reported previously^18^. Briefly, ultrasound-guided tumor biopsies were obtained from patients undergoing diagnostic liver biopsy at the University Hospital Basel. Written informed consent was obtained from all patients and the study was approved by the ethics committee of the northwestern part of Switzerland (Protocol Number EKNZ 2014-099). Tumor needle biopsy tissue was rapidly transferred to the lab, minced, and digested with 2.5 mg/mL collagenase IV (Sigma), 0.1 mg/mL DNase (Sigma) at 37 °C for 5 min. Digested biopsy/cell clusters were then seeded into reduced growth factor BME2 (Basement Membrane Extract, Type 2; Amsbio). After polymerization of BME2, expansion medium was added to the BME2 droplets. The composition of the medium is: advanced Dulbecco’s modified Eagle’s medium (DMEM)/F-12 (GIBCO) supplemented with 1:50 B-27 (GIBCO), 1:100 N-2 (GIBCO), 10 mM nicotinamide (Sigma), 1.25 mM *N*-acetyl-l-cysteine (Sigma), 10 nM [Leu15]-gastrin (Sigma), 10 mM forskolin (Tocris), 5 mM A83-01 (Tocris), 50 ng/mL EGF (PeproTech), 100 ng/mL FGF10 (PeproTech), 25 ng/mL HGF (PeproTech), and 10% RSpo1-conditioned medium (homemade). Tumor organoids were passaged by dissociation with 0.25% Trypsin-EDTA (GIBCO).

### Srf treatment of tumor organoids

One thousand cells per well were seeded in a 384-well plate and Srf added 24 h later. After 6 days of drug treatment, cell viability was assessed with CellTiterGlo 3D according to the manufacturer’s instructions.

### Autophagy study in vivo

Mice carrying floxed alleles of Lats1 (*Lats1*^fl/fl^) were kindly provided by Randy L. Johnson (M.D. Anderson Cancer Center) via Georg Halder (VIB-KU Leuven Center for Cancer Biology). Mice expressing liver-specific-cre recombinase (Alb-cre) driven by the albumin promoter (JAX stock #003574) was kindly provided by Markus H. Heim. *Lats1* conditional knock mice (*Lats1*^fl/fl^, Alb-cre) were generated by crossing *Lats1*^fl/fl^ mice with Alb-cre mice in a C57BL/6J background. Mice were bred and maintained under specific and opportunistic pathogen-free (SOPF) facility with food and water ad libitum. All in vivo experiments were performed under approval number 2839 within the Swiss Federal Animal Welfare Law.

To block autophagic flux, mice were intraperitoneally (i.p.) injected with CQ (30 mg/kg in phosphate-buffered saline, PBS) and liver tissues were collected for analysis 4 h after injection. To induce autophagic flux, mice were i.p. injected with rapamycin (2 mg/kg in PBS containing 5% PEG400/5% Tween-80/4% ethanol) twice a day (0600 and 1800 h) followed by fasting for 12 h and extraction of liver tissue for analysis.

### Cloning

To establish the hybrid LATS2 chimera construct, the LATS2 N terminus (amino acids 1–148), LATS1 domain (amino acids 151–554), and LATS2 C terminus (amino acids 514–1088) were PCR-amplified and assembled into pcDNA3-derived vectors using NEB Builder HiFi kit (NEB).

### Cell viability and colony formation assays

Short-term cell viability was measured using CellTiter-Fluor cell viability assay (Promega) with a SpectraMAX plate reader according to the manufacturer’s instructions. Viability of ex vivo organoid lines in response to Srf was determined according to the protocol described in ref. ^18^. Long-term cell viability and proliferation was performed using colony formation assays. Briefly, 200–1000 cells seeded in triplicate on 12-well plates were cultured for 2–3 weeks. Completed culture medium supplemented with Srf or DMSO (dimethyl sulfoxide) was refreshed every 3 days for the first week and every 2 days from the second week onwards. To visualize differences in cell growth, crystal violet staining was performed. Briefly, cells were fixed with 4% paraformaldehyde for 20 min at room temperature, washed twice with H_2_O, and stained with crystal violet (1 mg/mL dissolved in 10% ethanol) for 20 min. After washing with H_2_O, plates were left to dry at room temperature. The cell stained crystal violet was retrieved with 10% acetic acid and absorbance at 595 nm was measured using a SpectraMAX plate reader.

### Tumor transplantation and Srf therapy

NSG mice were maintained in SOPF facility with food and water ad libitum. Huh7 cells (5 × 10^5^ in 200 μL DMEM:Matrigel (1:1 ratio)) were implanted into the left flanks. When tumors were palpable, mice were randomly grouped into vehicle or Srf treatment cohorts. When tumor volumes reached 100 mm^3^, Srf (30 mg/kg) or vehicle control was administrated daily via oral gavage for 4 weeks. Tumor diameters were measured twice a week using a caliper, and tumor volumes were calculated as follows: volume = *d*^2^*D/2, where *d* and *D* is the shorter and longer tumor diameter, respectively. All in vivo experiments were performed under approval number 2839 within the Swiss Federal Animal Welfare Law.

### Analysis of autophagy

For LC3B staining, cells were transfected and seeded on glass coverslips. After treatment, cells were washed in PBS and fixed with ice-cold methanol for 20 min. After PBS washes, fixed cells were incubated in 0.1% Triton X-100 in PBS for 5 min, followed by blocking with 0.1% Trition X-100 in 3% bovine serum albumin (BSA) for 1 h. Endogenous LC3B were detected by incubation of fixed cells with anti-LC3B antibody (CST3868, 1:200) overnight, followed with anti-rabbit Alexa 488 secondary antibody (Invitrogen 1:300). After 4′,6-diamidino-2-phenylindole (DAPI) staining, cells were washed and mounted in fluorescence mounting medium (Dako S3023). Images were acquired on a Leica DMI 4000 microscope. For quantifications, more than 5 fields (at least 100 cells in total) were randomly selected based on DAPI staining and the number of LC3B dots per cell was quantified using ImageJ software.

Autophagy detection using flow cytometry was performed according to the protocol described in ref. ^27^. Briefly, U2OS-GFP-LC3 cells were trypsinized and incubated with 0.5% saporin in PBS for 5 min. Cells were washed and suspended for analysis using a BD FACS Canto II flow cytometer. Data were analyzed with Flowjo software.

### Protein lysis, immunoprecipitation, ubiquitination, and immunoblot analysis

Cells were lysed with RIPA buffer (Sigma R0278) supplemented with additional 2% NP-40. Cell lysates were sonicated before protein concentration determination (BCA protein assay kit, Pierce 23225). Equal amount of protein was fractionated by SDS-polyacrylamide gel eletrophoresis and transferred onto a polyvinylidene difluoride membrane, which was blocked with 5% milk in Tris-buffered saline (TBS) followed with desired antibodies in 5% BSA-TBS. The protocol for co-immunoprecipitation has been described previously^58^. Briefly, cells were lysed with Cell Signaling Technology (CST) lysis buffer (CST9803) supplemented with 1 mM phenylmethylsulfonyl fluoride and incubated with specific antibodies with protein A/G-Sepharose overnight. After three times washing with CST lysis, the precipitated proteins were eluted with SDS-loading buffer and analyzed by immunoblotting. For the ubiquitination assay, cells transfected with plasmids were lysed with RIPA buffer supplemented with additional 0.1% SDS to a final concentration of 0.2% SDS, followed by standard immunoprecipitation protocols. Chemiluminescence was detected with X-Ray films or Fusion FX (Analis). Image J software was used to quantify the immunoblots by densitometry. Information on the antibodies used is presented in Supplementary Dataset 2.

### Identification of ubiquitination sites by mass spectrometry

MS analysis was performed in the proteomics core facility of the Biozentrum, University of Basel. Briefly, HEK cells were transfected with Flag-tagged Beclin-1 and HA-tagged ubiquitin together with either empty vector or vector encoding LATS1. After lysis and immunoprecipitation, proteins were eluted with 600 µl 100 mM trimethylamine, pH 11. The eluate was dried in a SpeedVac and fully resuspended in 100 μL of 50 mM Tris-HCl (pH 7.5) with sonication. Samples were heated up for 10 min and cooled down followed by incubated with chloroacetamide (1 μL per samples). Proteins were subjected to endoproteinase LysC (1:100 (w/w), Wako) digestion at 37 °C for 4 h, then subjected to trypsin digestion (0.5 μg/μL; 1:50; w/w) at 37 °C overnight. The samples were then subjected to liquid chromatography–tandem MS (LC-MS/MS).

The acquired raw-files were imported into the Progenesis QI software (v2.0, Nonlinear Dynamics Limited), which was used to extract peptide precursor ion intensities across all samples applying the default parameters. The generated mgf-files were searched using MASCOT (Version: 2.4.1) against a decoy database containing normal and reverse sequences of the predicted SwissProt entries of Homo sapiens (www.ebi.ac.uk, release date 2016/25/05) and commonly observed contaminants (in total 41,170 sequences for Homo sapiens) generated using the SequenceReverser tool from the MaxQuant software (Version 1.0.13.13). The search criteria were set as follows: semi tryptic specificity was required (cleavage after lysine or arginine residues, unless followed by proline); four missed cleavages were allowed; carbamidomethylation (C) was set as fixed modification; oxidation (M) and GlyGly (K) were applied as variable modifications; mass tolerance of 10 p.p.m. (precursor) and 0.02 Da (fragments). The database search results were filtered using the ion score to set the false discovery rate (FDR) to 1% on the peptide and protein level, respectively, based on the number of reverse protein sequence hits in the datasets. The relative quantitative data obtained were normalized and statistically analyzed using our in-house script as above^59^. In addition, Scaffold (version Scaffold_4.8.7, Proteome Software Inc., Portland, OR) was used to validate MS/MS based peptide and protein identifications. Peptide identifications were accepted if they could be established at greater than 93.0% probability by the Scaffold Local FDR algorithm. Protein identifications were accepted if they could be established at greater than 98.0% probability to achieve an FDR < 1.0% and contained at least two identified peptides. Protein probabilities were assigned by the Protein Prophet algorithm^60^. Proteins that contained similar peptides and could not be differentiated based on MS/MS analysis alone were grouped to satisfy the principles of parsimony.

### RNA extraction and sequencing analysis

RNA from siRNA-transfected and Srf-treated cells was extracted in biological triplicates using miRNeasy Mini kit (Qiagen) according to the manufacturer’s instructions. RNA quality control was performed using a fragment analyser and the standard or high-sensitivity RNA analysis kits (Labgene; DNF-471-0500 or DNF-472-0500). RNA concentrations were measured using the Quanti-iTTM RiboGreen RNA assay Kit (Life Technologies/Thermo Fisher Scientific). A total of 200 ng of RNA was utilized for library preparation with the TruSeq stranded total RNA LT sample prep Kit (Illumina). Poly-A+ RNA was sequenced with HiSeq SBS Kit v4 (Illumina) on an Illumina HiSeq 2500 using protocols defined by the manufacturer.

Single-end RNA-seq reads (81-mers) were mapped to the human genome assembly, version hg19 (GRCh37.75), with RNA-STAR^61^, with default parameters except for allowing only unique hits to genome (outFilterMultimapNmax = 1) and filtering reads without evidence in spliced junction table (outFilterType = “BySJout”). Expression levels per gene (counts over exons) for the RefSeq mRNA coordinates from UCSC (genome.ucsc.edu, downloaded in December 2015) were quantified using qCount function from QuasR package (version 1.12.0). The differentially expressed genes were identified using the edgeR package (version 3.14.0). Genes with p-values smaller than 0.05 and minimum log2 fold changes of ±0.58 were considered as differentially regulated and were used for downstream functional and pathway enrichment analysis.

### Functional enrichment analysis

We performed functional enrichment analysis of differentially expressed genes for biological processes or pathways in R using several publicly available Bioconductor resources including org.Hs.eg.db (version 3.3.0), GO.db (version 3.4.1), GOstats (version 2.42.0)^62^, KEGG.db (version 3.2.3), and ReactomePA (version 1.16.2)^63^. The significance of each biological processes or pathways identified was calculated using the hypergeometric test (equivalent to Fisher’s exact test) and those with *p*-values ≤ 0.05 were considered significant.

### Gene set enrichment analysis

The GSE A analysis was performed using the JAVA application of the Broad Institute version 3.0 (http://www.broadinstitute.org/gsea). The gene sets used for the analysis were derived from GO annotations, and pathways were obtained from the GO (http://www.geneontology.org/) and KEGG (http://www.genome.jp/kegg/) databases.

### Patient material and ethics

All relevant ethical regulations were strictly followed in this study. All the analysis using human tissue samples reported in this study were approved by the ethics commission of Northwestern Switzerland (EKNZ, approval number 361/12).

### Re-analysis of transcriptomic profiling data

RNA-seq gene expression values were retrieved from om TCGA Liver dataset^64^ using the cbioportal (http://www.cbioportal.org, accessed 12/05/2017) website^65^. The dataset included 364 and 314 HCCs with overall survival and disease-free survival information, respectively. Survival analyses were performed using the Kaplan–Meier method and the log-rank test. The cut-off was defined as previously described^66^.

### Statistical analysis

All statistical tests were two-sided. Specific details on the statistical method are provided in each figure legend. Data are presented as mean. Bar plots with error bars represent mean ± SD. Statistical significance was defined as *P < 0.05, **P < 0.01, ***P < 0.001. All analyses were performed using Excel or Graphpad Prism 6.0 (Graphpad Software, Inc., La Jolla, CA) or SPSS v.20 (Endicott, New York, NY).

### Reporting summary

Further information on research design is available in the Nature Research Reporting Summary linked to this article.

## Supplementary information

Supplementary Information

Description of Additonal Supplementary Files

Supplementary Data 1

Supplementary Data 2

Reporting Summary

## Data Availability

The mass spectrometry proteomics data have been deposited to the ProteomeXchange Consortium via the PRIDE partner repository with the dataset identifier PXD013159 and 10.6019/PXD013159. The RNA-seq files are deposited on GEO database with an accession number GSE117116. Data sets with accession numbers GSE71873 and Uniprot access numbers Q14457 for human, O8859 for Mouse, F1RCP1 for Fish, Q4A1L3 for Frog, Q22592 for Worm, Q9VCE1 for fly, Q02948 for yeast, and TCGA Liver datasets have been used in the study. The source data underlying Figs. 1a, c-d, f, i, 2b-e and g-h, 3a-i, 4c-e, 5a-c and e-f, 6a-c, e and g-i, 7a-f, and Supplementary Figs. 1a-c, e-g, k and l-n, 3b-g, 4a-e and g, 5b-d, f, h, 6c, 7a-i, 8a-d and f, 9a-i, and 10c are provided in Source Data file.

## Source Data

Source Data
