# Supplementary material for: LATS1 but not LATS2 represses autophagy by a kinase-independent scaffold function
Source: Nat Commun. 2019 Dec 17;10:5755. doi: 10.1038/s41467-019-13591-7 (PMC6917744; doi:10.1038/s41467-019-13591-7)
Supplement: Supplementary file 1 — Supplementary Information [file 41467_2019_13591_MOESM1_ESM.pdf]

Supplementary Information

LATS1 but not LATS2 represses autophagy by a kinase-independent scaffold function

Fengyuan Tang, Ruize Gao, Beena Jeevan-Raj, Christof B. Wyss, Ravi K. R. Kalathur, Salvatore Piscuoglio, Charlotte K.Y. Ng, Sravanth K. Hindupur, Sandro Nuciforo, Eva Dazert, Thomas Brock, Shuang Song, David Buechel, Marco F. Morini, Alexander Hergovich, Patrick Matthias, Dae-Sik Lim, Luigi M. Terracciano, Markus H. Heim, Michael N. Hall, and Gerhard Christofori

Supplementary Figures

Supplementary Figure 1, Tang et al.

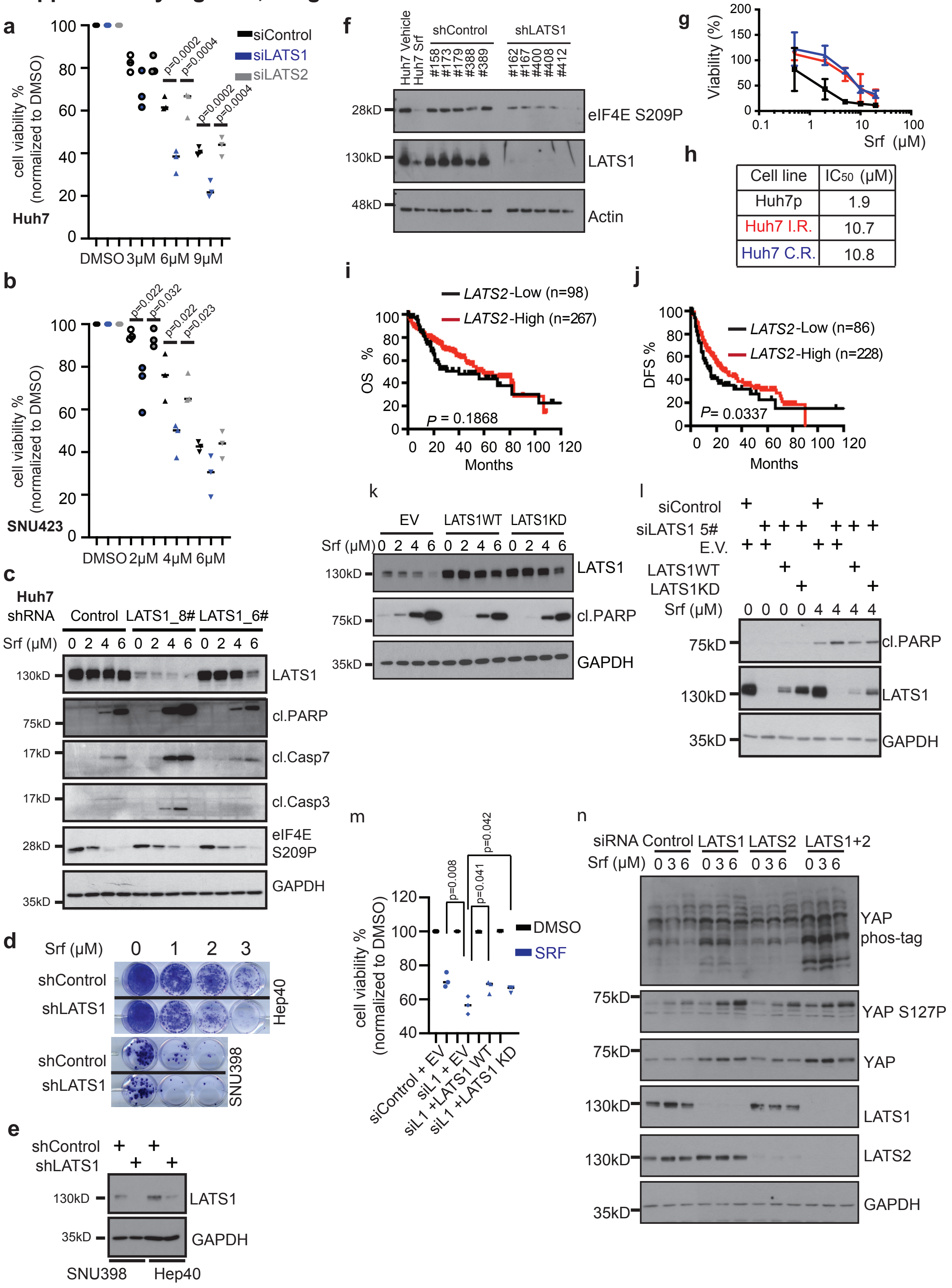

Suppl. Figure 1. A pro-survival role of LATS1 in HCC cells in response to sorafenib treatment.

(a,b) siRNA-mediated ablation of LATS1 but not LATS2 expression leads to reduced cell viability in response to sorafenib in Huh7 cells (a) and SNU423 cells (b). Huh7 and SNU-423 cells were transfected with siControl or with siRNAs against LATS1 or LATS2 and exposed to DMSO vehicle control or increasing concentrations of sorafenib and cell viability was determined using CellTiter-Fluor cell viability assay (Promega). Bar graphs represent pooled results from three independent experiments. Statistical significance was calculated by two-tailed, paired t-test.

(c) shRNA-mediated depletion of LATS1 promotes apoptosis in response to sorafenib treatment. Huh7 cells stably expressing either shControl (shRNA targeting LacZ) or shRNAs against LATS1 were treated with DMSO or increasing concentrations of sorafenib, and cell lysates were then immunoblotted for LATS1, cleaved PARP, cleaved caspase 3 and 7, and for phosphorylation of eIF4E (S209P). Immunoblotting for GAPDH was uses as loading control. Note: while shLATS1_8# resulted in efficient knock-down, shLATS1_6# hardly achieved any knock-down. Thus, shLATS1_6# served as another internal control. Results represent three independent experiments.

(d,e) shRNA-mediated loss of LATS1 expression results in impaired colony formation of Hep40 and SNU398 cells in response to increasing doses of sorafenib (Srf). Colony formation was determined in Huh7 cells expressing either shControl (shRNA targeting LacZ) or a shRNA against LATS1(8#) and exposed to increasing concentrations of sorafenib (d). shRNA-mediated depletion of LATS1 (8#) was confirmed by immunoblotting (e). GAPDH was used as loading control. The results represent three independent experiments.

(f) Protein analysis of tumor samples derived from Figure 1c. Huh7 parental cells derived tumors were treated with vehicle or sorafenib (30mg/kg, p.o., daily) for 5 days used as controls to demonstrate an acute effect of sorafenib on eIF4E S209 phosphorylation and LATS1 *in vivo*. Note: Sorafenib downregulates eIF4E S209 phosphorylation and LATS1 protein. Tumors derived from Huh7-shControl cells after 4 weeks sorafenib treatment re-gained eIF4E S209 phosphorylation and LATS1 expression, indicating emergence of sorafenib resistance in these tumors. Results represent three independent experiments.

(g,h) Characterization of sorafenib-resistant cells. Huh7p, IR and CR cells were exposed to DMSO vehicle control or increasing concentrations of sorafenib and cell viability was determined using CellTiter-Fluor cell viability assay (Promega) (g). IC50 calculation were achieved by Graphpad(h). Results were pooled from three independent experiments.

(i,j) Correlation between HCC patient survival and LATS2 expression. Shown are Kaplan-Meyer analyses of overall survival (OS) (i) and disease-free survival (DFS) (j) of LATS2-high and LATS2-low-expressing patient samples from the TCGA database. Statistical significance was determined by log-rank (Mantel-Cox) test.

(k) The forced expression of both wild-type (WT) LATS1 and a kinase-dead (KD D846A) version of LATS1 inhibits sorafenib-induced cell death in Hep3B cells. Cell were treated with DMSO or increased concentration of sorafenib for 24 hours and lysates were then immunoblotted for LATS1, cleaved PARP. Immunoblotting for GAPDH was uses as loading control. Results represent three independent experiments.

(l,m) Huh7 Cells were transfected with indicated siRNA and siRNA refractory cDNA as indicated in a step-wise manner. 24 hours later, the cells were trypsinized and seeded in 6cm dish (left panel) or 96 well plate (right panel). 24 hours later, were treated with sorafenib for another 48 hours as indicated. (l) Protein samples were harvested for western blot with indicated antibodies. (m) cell viability was measured by Promega cell titer fluoro viability assay kit. Statistical significance was calculated by two-tailed, paired t-test. Results were pooled from three independent experiments.

(n) siRNA-mediated ablation of LATS1 and LATS2 results in YAP activation. Huh7 cells were transfected with siControl or siRNAs against LATS1 or LATS2 and treated with DMSO vehicle or increasing concentrations of sorafenib (Srf) as indicated. 48 hours later, cells were lysed and analyzed by immunoblotting for YAP. Phosphorylated YAP was further determined by phos-tag gel electrophoresis. Immunoblotting for GAPDH was used as loading control. The results represent three independent experiments.

Supplementary Figure 2, Tang et al.

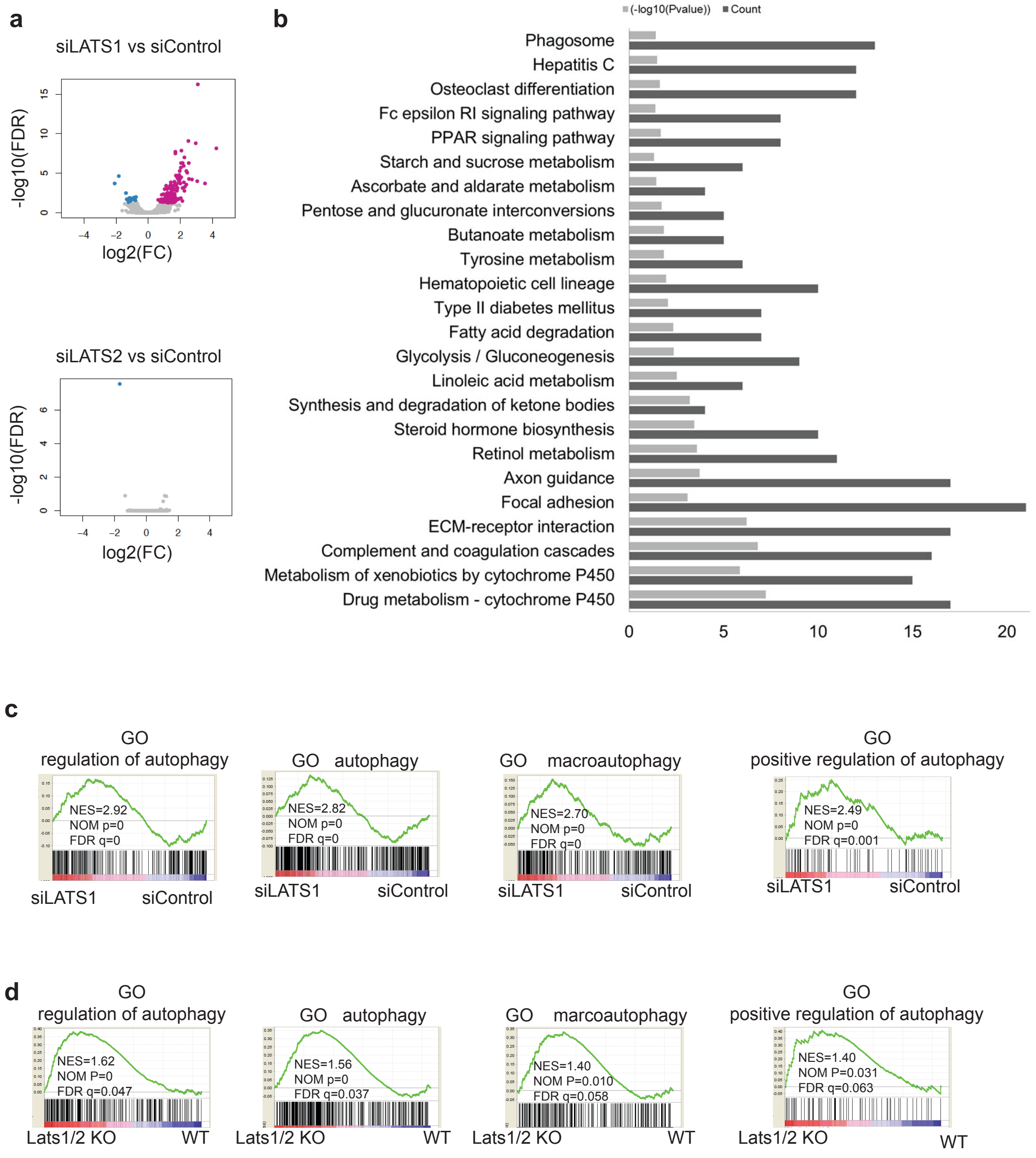

Suppl. Figure 2. LATS1-regulated gene expression and signaling pathways in HCC cells.
RNA was extracted from Huh7 cells expressing either siControl or siRNAs against LATS1 or LATS2 and subjected to next generation RNA sequencing.
(a) Volcano plots demonstrate that LATS1, but not LATS2, massively regulates gene expression in Huh7 cells.
(b) Functional pathway analysis of the genes regulated by LATS1 depletion reveals a number of biological processes, with the phagosome among the top upregulated signalling pathways upon loss of LATS1 expression.
(c) Gene set enrichment analysis (GSEA) reveals that loss of LATS1 expression positively correlates with the general regulation of autophagy and macro-autophagy and the positive regulation of autophagy. Statistical analysis was determined by GSEA algorithm.
(d) Gene set enrichment analysis (GSEA) reveals that the genetic knock-out of Lats1 and Lats2 in mouse hepatoblasts (GSE71873) positively correlates with the general regulation of autophagy and macro-autophagy and the positive regulation of autophagy. Statistical analysis was determined by GSEA algorithm.

Supplementary Figure 3, Tang et al.

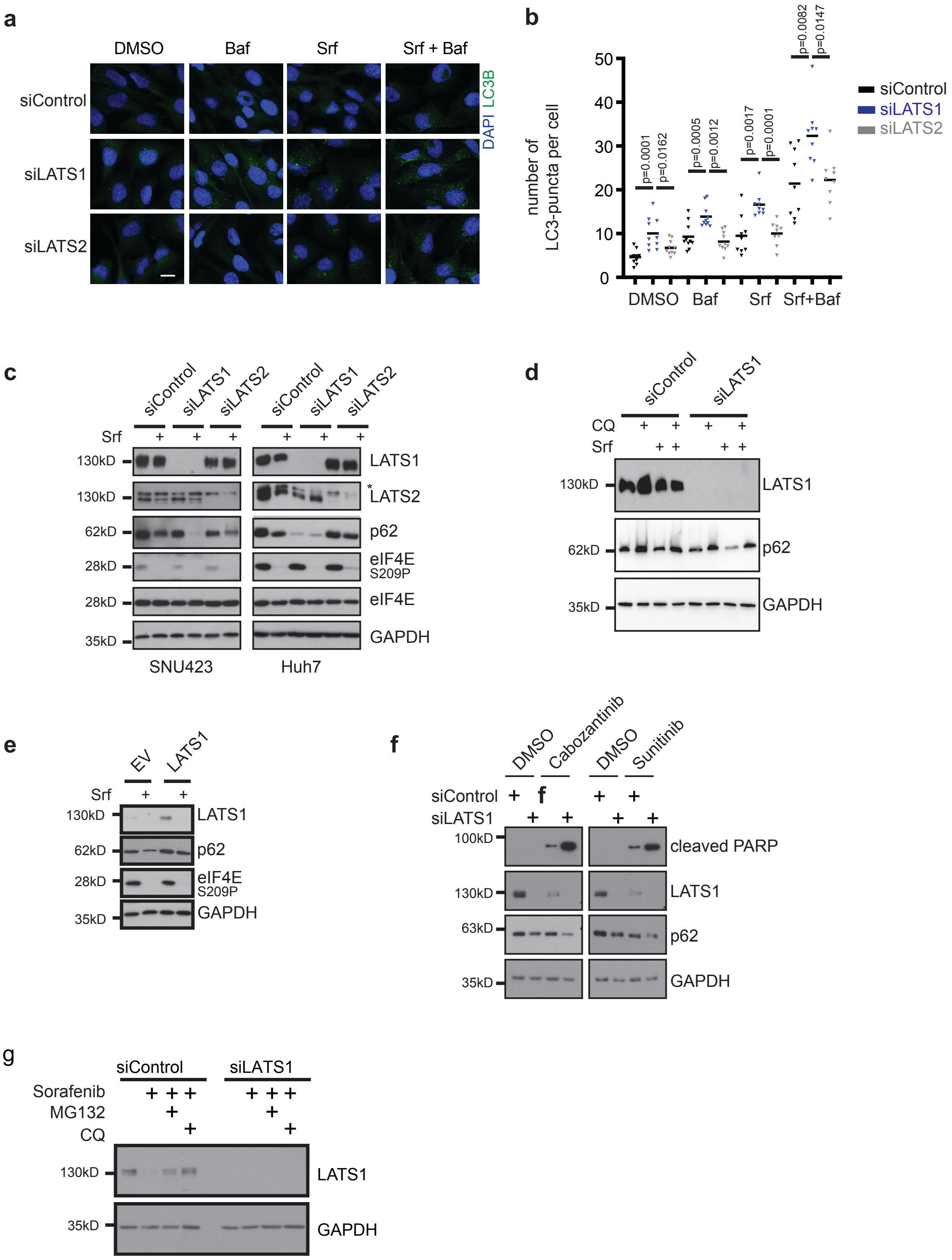

Suppl. Figure 3. LATS1 represses sorafenib-induced autophagy in HCC cells.

(a,b) Immunofluorescence microscopy analysis of LC3B puncta in SNU423 cells transfected either with siControl or with siRNAs against LATS1 or LATS2 and treated with DMSO vehicle control, sorafenib (6µM), bafilomycin A1 (0.1µM), or a combination of both. Sorafenib treatment was for 40 hours, while exposure to bafilomycin A1 was for 2 hours before cell fixation. LC3B puncta were stained and visualized by immunofluorescence microscopy. Shown are representative images (a) and the quantification of LC3B puncta numbers (b) pooled from four independent experiments. Statistical analysis was calculated by one-way ANOVA. *** P<0.001, **P<0.01. Scale bar, 25µm.

(**c**) Immunoblotting analysis of autophagic flux marker p62 in siRNA transfected SNU423 and Huh7 cells in response to sorafenib. SNU423 and Huh7 cells were transfected with either siControl or siRNAs against LATS1 and LATS2 and treated with DMSO vehicle control or sorafenib (6µM) for 40 hours. Cell lysates were then immunoblotted for LATS1, LATS2, p62, phosphorylated eIF4E (S209P) and total eIF4e. GAPDH was used as a loading control. Immunoblots represent three independent experiments.

(d) LATS1 inhibits sorafenib-induced autophagic degradation of p62. Huh7 cells were transfected with indicated siRNAs and treated with DMSO vehicle control or sorafenib (6µM) for 40 hours. Cells were additionally treated with chloroquine (10µM, 3 hours) and cell lysates were then immunoblotted for LATS1 and p62. GAPDH was used as a loading control. Results represent three independent experiments.

(e) The forced expression of wild-type LATS1 inhibits sorafenib-induced autophagic flux (degradation of p62) in Hep3B cells. Hep3B cells were transfected with empty vector (EV) or with a vector encoding for LATS1 and treated with DMSO vehicle control or sorafenib (6µM) for 40 hours. Cell lysates were then immunoblotted for LATS1, p62 and phosphorylated eIF4E (S209P). GAPDH was used as a loading control. Results represent three independent experiments.

(f) LATS1 inhibition of target therapies-induced autophagic cell death in HCC is a generic phenotype. Huh7 cells were transfected with indicated siRNAs and treated with DMSO vehicle control or Cabozantinib (6µM) or Suntinib (6µM) for 40 hours. Cell lysates were then immunoblotted for LATS1, p62 and cleaved PARP (cl. PARP). GAPDH was used as a loading control. Results represent three independent experiments.

(g) Sorafenib induces autophagic degradation of LATS1. Huh7 cells were transfected with indicated siRNAs and treated with DMSO vehicle control or sorafenib (6µM) for 40 hours. Cells were additionally treated with MG132 (proteasomal inhibitor, 5µM for 10 hours) or chloroquine (autophagy inhibitor, 10µM for 10 hours) before harvesting. Cell lysates were then immunoblotted for LATS1. GAPDH was used as a loading control. Results represent three independent experiments.

Supplementary Figure 4, Tang et al.

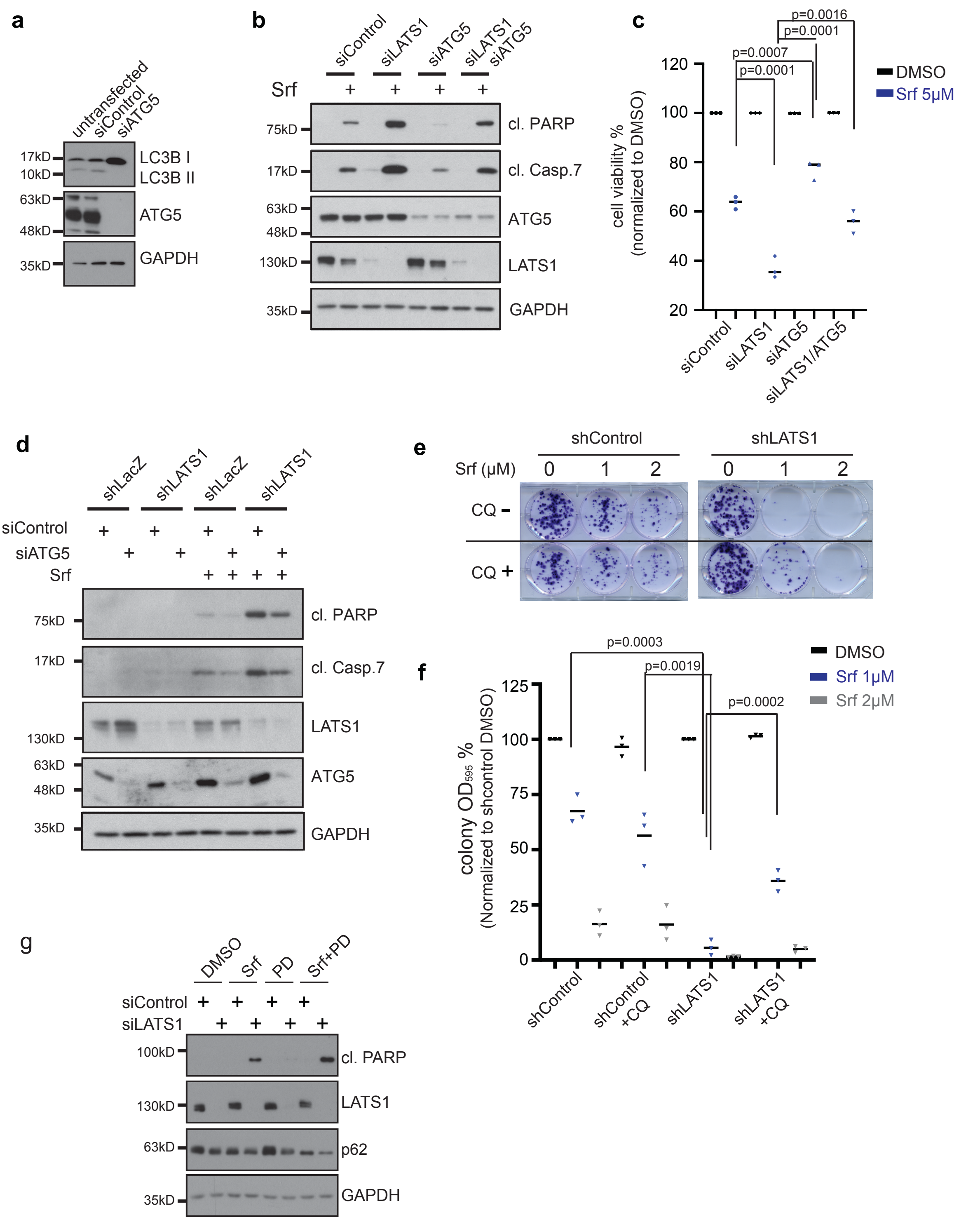

Suppl. Figure 4. LATS1 restricts sorafenib-induced lethal autophagy.

(a) ATG5 is essential for autophagy induction. Huh7 cells were transfected with RNAiMax alone or Control siRNA or siRNAs against ATG5. Cells were lysed 48 hours later and immunoblotted with LC3BI/II and ATG5. GAPDH was used as loading control. Immunoblots represent three independent experiments.

(b) Sorafenib-induced apoptosis is increased by the loss of LATS1 and requires the activity of ATG5. Huh7 cells were transfected with siControl or siRNAs against LATS1, ATG5 or both and treated with DMSO vehicle control or sorafenib (6µM) for 40 hours. Cells were lysed and immunoblotted with antibodies against cleaved PARP, cleaved caspase 7, ATG5 and LATS1. Immunoblotting for GAPDH was used as loading control. Immunoblots represent three independent experiments.

(**c**) Sorafenib-induced cell survival is decreased by the loss of LATS1 yet increased by the loss of ATG5. Cells treated as described in (B) were analysed for cell viability by using the using CellTiter-Fluor cell viability assay (Promega). Bar graphs represent pools from four independent experiments. Statistical significance was calculated using one-way ANOVA..

(**d**) Sorafenib-induced apoptosis is increased by the loss of LATS1 and requires the activity of ATG5. Huh7 cells stably expressing shLacZ as control or shRNA against LATS1 (shLATS1_8#) were transfected with siControl or with siRNA against ATG5 and treated with DMSO vehicle control or sorafenib for 40 hours. Cell lysates were prepared and immunoblotted for cleaved PARP (cl. PARP), cleaved caspase 7 (cl. Casp7), LATS1 and ATG5. Immunoblotting for GAPDH was used as loading control. Immunoblots represent three independent experiments.

(**e**) Inhibition of autophagy partially rescues sorafenib-induced in LATS1-depleted cells. Huh7 cells expressing either shControl or shRNA against LATS1 (shLATS1_8#) were exposed to increasing concentrations of sorafenib and additionally treated with chloroquine (CQ, 1.5µM). Colony formation of cells was determined by crystal violet staining. Pictures represent three independent experiments.

(f) Quantification of the experiments described in (e) by determining the absorbance of crystal violet staining with a SpectraMAX plate reader. The results represent pools from three independent experiments. Statistical significance was calculated by one-way ANOVA..

(g) Huh7 cells were transfected with siControl or siRNAs against LATS1 and treated with DMSO vehicle control or sorafenib (6µM) or the MEK inhibitor PD032901 (10µM) or a combination of Sorafenib and PD032901 for 40 hours. Cells were lysed and immunoblotted with antibodies against cleaved PARP, LATS1, p62. Immunoblotting for GAPDH was used as loading control. Immunoblots represent three independent experiments.

Supplementary Figure 5, Tang et al.

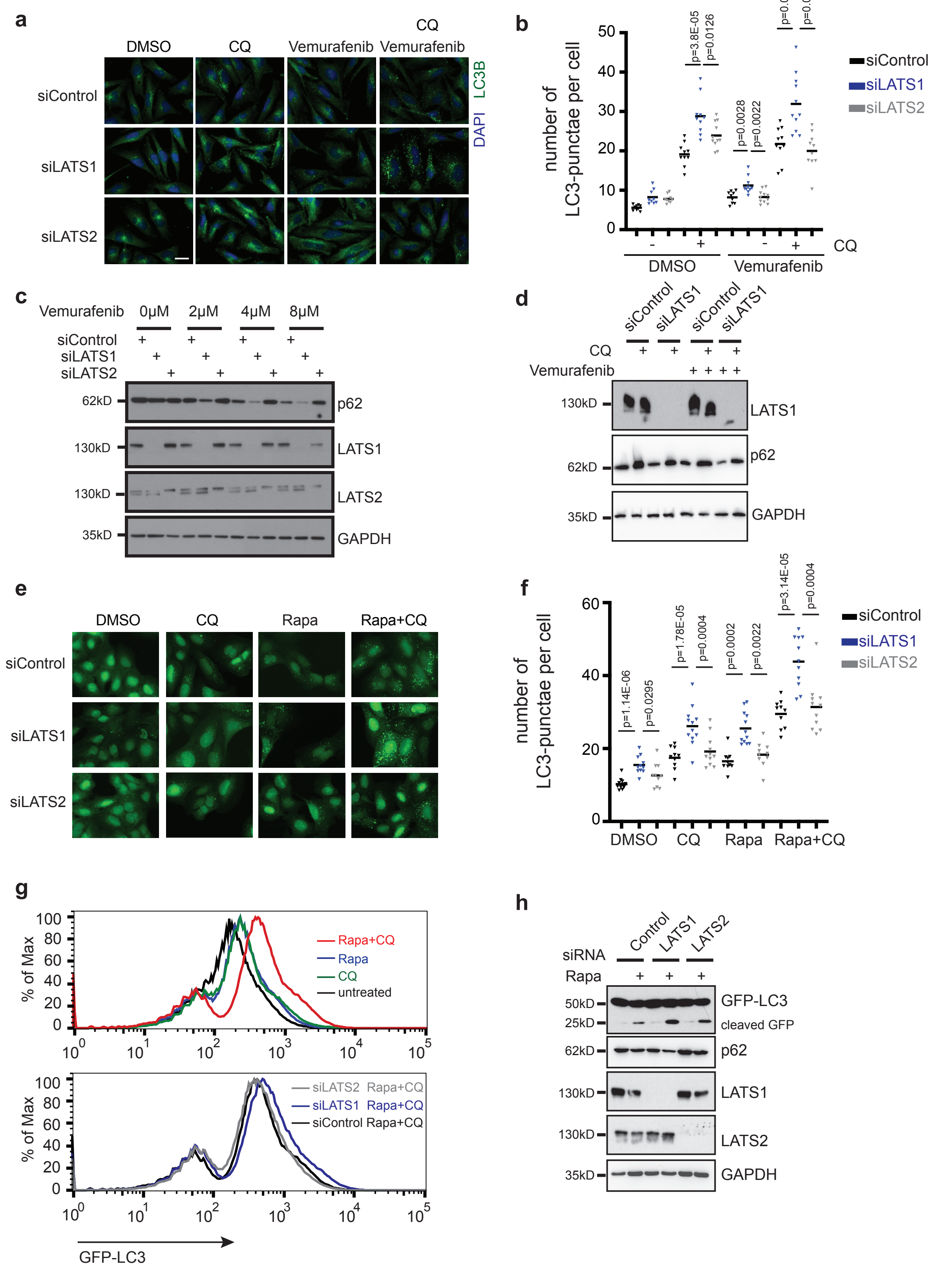

Suppl. Figure 5. A general inhibitory role of LATS1 in autophagy.

(a,b) Vemurafinib treatment-induced autophagy induction is increased by the loss of LATS1 but not LATS2. Immunofluorescence microscopy analysis of LC3B puncta in A2058 melanoma cells in response to vemurafinib and/or chloroquine treatment. A2058 cells were transfected with Control siRNA or siRNAs against LATS1 or LATS2 and then treated with DMSO or Vemurafenib (2μM) for 40 hours. Cells were additionally treated with chloroquine (CQ, 10μM) for 2 hours before being fixed. Shown are representative images of LC3B puncta staining (a) and quantification of LC3B puncta numbers (b) pooled from three independent experiments. Statistical analysis was calculated using one-way ANOVA.. Scale bar, 25μm.

(c) Vemurafinib-induced autophagic flux is increased by the loss of LATS1 but not LATS2. A2058 melanoma cells were transfected with control siRNA or siRNAs against LATS1 or LATS2 and then treated with DMSO or increasing concentrations of Vemurafenib as indicated. Cell lysates were prepared 40 hours later and immunoblotted for cleaved p62, LATS1, and LATS2. Immunoblotting for GAPDH was used as loading control. Immunoblots represent three independent experiments.

(d) LATS1 inhibits Vermurafinib-induced autophagic degradation of p62. A2058 cells were transfected with indicated siRNAs and treated with DMSO vehicle control or Vemurafinib (4μM) for 40 hours. Cells were additionally treated with chloroquine (10μM, 3 hours) and cell lysates were then immunoblotted for LATS1, p62. GAPDH was used as a loading control. Results represent three independent experiments.

(e,f) Rapamycin-induced autophagy induction is increased by the loss of LATS1 but not LATS2. Immunofluorescence microscopy analysis of LC3B puncta in U2OS GFP-LC3 cells in response to Rapamycin and/or chloroquine treatment. U2OS cells were transfected with control siRNA or siRNAs against LATS1 or LATS2 and then treated with DMSO or Rapamycin (Rapa, 100nM) for 16 hours. Cells were additionally treated with chloroquine (CQ, 10μM) for 2 hours before being fixed. Shown are representative images of LC3 puncta staining (d) and quantification of LC3 puncta numbers (e) pooled from three independent experiments. Statistical analysis was calculated using one-way ANOVA.. Scale bar, 25μm.

(g) Flow cytometry analysis of autophagy induction in U2OS-GFP-LC3 cells indicates that LATS1, but not LATS2, inhibits rapamycin-induced autophagy induction. Cells were transfected with Control siRNA or siRNA against LATS1 or LATS2 and then treated with Rapamycin (Rapa, 100nM) for 16 hours. Cells were additionally treated alone or in combination with chloroquine (CQ, 10μM) for 2 hours before analysis. Cells were analyzed with a BD FACSCanto II flow cytometer, and the results were processed with FlowJo software. Results represent three independent experiments.

(h) Analysis of autophagic flux activity in U2OS-GFP-LC3 cells in response to Rapamycin upon loss of LATS1 and LATS2. Degradation of p62 and cleavage of GFP-LC3 serves as markers for autophagic flux activity. Cells were transfected with control siRNA or siRNA against LATS1 or LATS2 and then

treated with rapamycin (Rapa, 100nM) for 16 hours. Cell lysate were prepared and analyzed by immunoblotting using antibodies against LATS1, LATS2, p62, GAPDH and GFP. GAPDH was used as a loading control. Results represent three independent experiments.

Supplementary Figure 6, Tang et al.

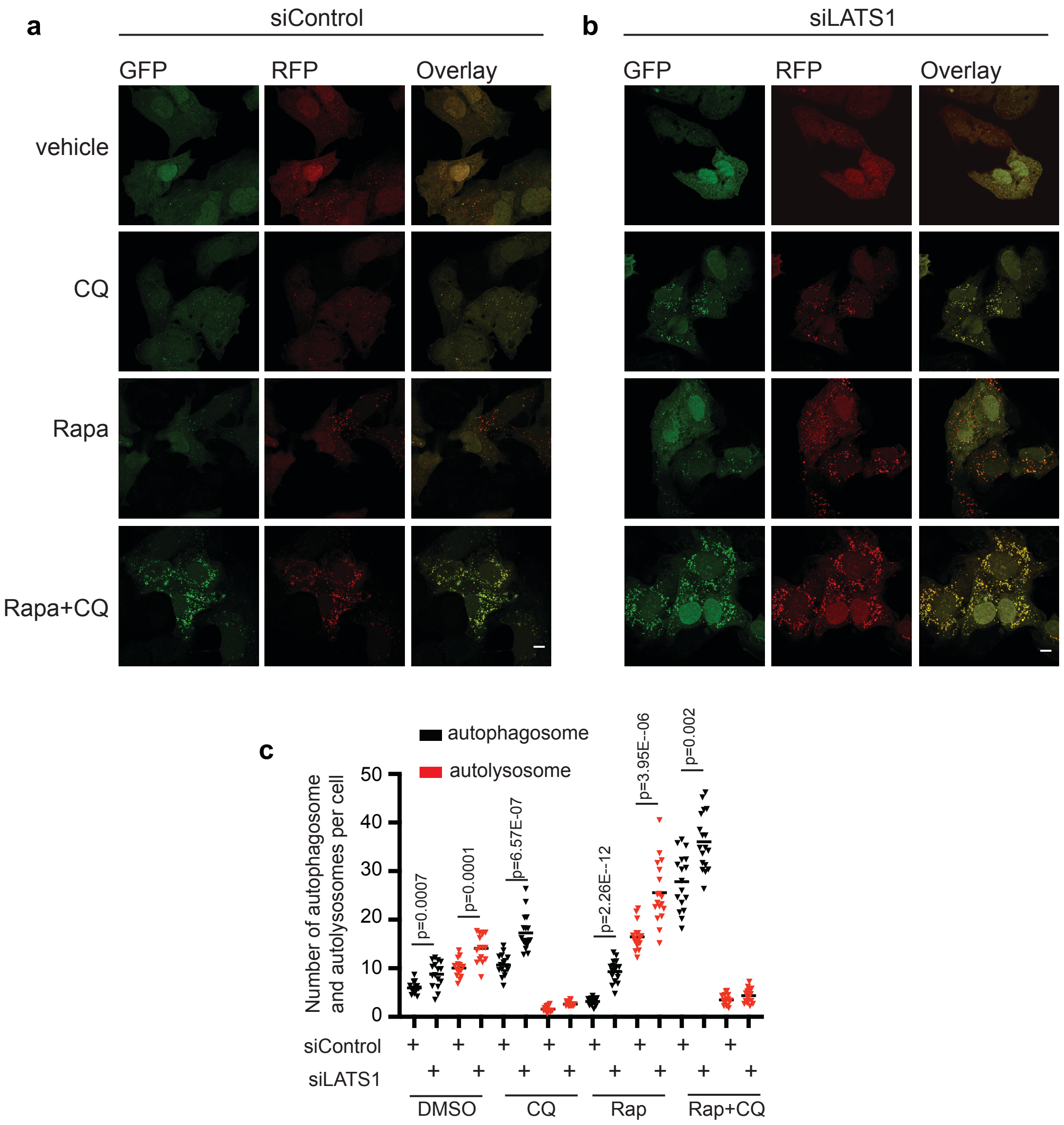

Suppl. Figure 6. LATS1 regulates rapamycin-induced autophagy dynamics in U2OS cells.
A pool of U2OS cells stably expressing a RFP-GFP-LC3 fusion construct was established by transfection of U2OS cells with ptfLC3 (Plasmid #21074) and selected with Neomycin (500µg/ml). U2OS-RFP-GFP-LC3 cells were transfected with control siRNA or siRNAs against LATS1 and then treated with DMSO or Rapamycin (Rapa, 100nM) for 16 hours. Cells were additionally treated with chloroquine (CQ, 10µM) for 2 hours before being fixed. Shown are representative images of yellow LC3 (autophagosomes) and red LC3 (autolysosomes) puncta staining (a, b) and quantification of puncta numbers (c) pooled from three independent experiments. Statistical analysis was calculated using one-way ANOVA. Scale bar, 25µm.

Supplementary Figure 7, Tang et al.

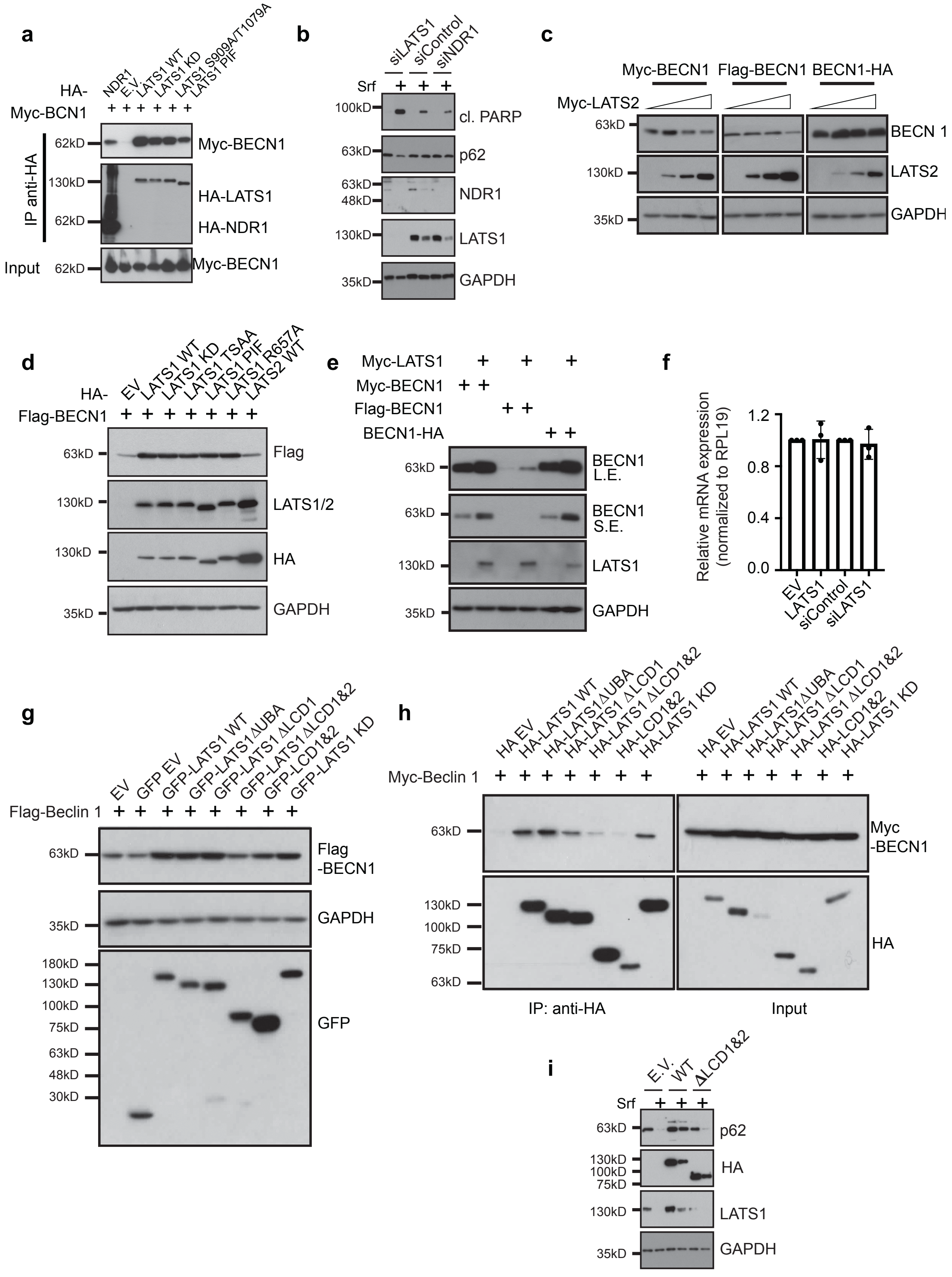

Suppl. Figure 7. LATS1, but not LATS2, stabilizes Beclin-1 in a kinase activity-independent manner.

(a) LATS1 interacts with Beclin-1 with a higher affinity in comparison to NDR1. HEK293T/17 cells were transfected with a vector encoding for Flag-tagged Beclin-1 (Flag-BECN1) together with empty vector (EV) or vectors encoding for HA-tagged wild-type NDR1, wild-type LATS1 or kinase-dead mutants (KD-D846A, STAA-S909A/T1069A, R657A) or a constitutive-active mutant (PIF) of LATS1, as indicated. 72 hours later, cell lysates were immunoprecipitated with antibodies against HA and then immunoblotted against Myc-Beclin-1 and HA-tagged NDR1 or LATS1. Input represents immunoblotting of cell lysates before immunoprecipitation. Results represent three independent experiments.

(b) Huh7 cells were transfected with siControl or siRNAs against LATS1 or NDR1 and treated with DMSO vehicle control or sorafenib (6μM) for 40 hours. Cells were lysed and immunoblotted with antibodies against cleaved PARP, p62, NDR1, LATS1. Immunoblotting for GAPDH was used as loading control. Immunoblots represent three independent experiments.

(c) LATS2 has no effect on the stabilization of Beclin-1. HEK293T/17 cells were transfected with vectors encoding for N-terminally Flag-tagged Beclin-1 (Flag-BECN1) or Myc-tagged Beclin-1 (Myc-BECN1) or C-terminally HA-tagged Beclin-1 (BECN1-HA) and increasing amounts of vector encoding Myc-tagged LATS2 (Myc-LATS2). 72 hours later, cell lysates were immunoblotted for tagged Beclin-1 and LATS2. Immunoblotting for GAPDH was used as loading control. Immunoblots represent three independent experiments.

(d) LATS1 stabilizes Beclin-1 in a kinase activity-independent manner. HEK293T/17 cells were transfected with a vector encoding for Flag-tagged Beclin-1 (Flag-BECN1) together with empty vector (EV) or vectors encoding for HA-tagged wild-type LATS1, wild-type LATS2 or kinase-dead mutants (KD-D846A, STAA-S909A/T1069A, R657A) or a constitutive-active mutant (PIF) of LATS1, as indicated. 72 hours later cells were lysed and analysed by immunoblotting for the expression of Beclin-1 (Flag), LATS1 and 2 (LATS1/2) and HA-tagged LATS mutants. GAPDH was used as loading control. Results represent three independent exepriments.

(e) LATS1 stabilizes Beclin-1, regardless of N-terminal or C-terminal tagging. HEK293T/17 cells were transfected with vectors encoding Myc-tagged LATS1 and/or N-terminally Flag-tagged Beclin-1 (Flag-BECN1) or Myc-tagged Beclin-1 (Myc-BECN1) or C-terminally HA-tagged Beclin-1 (BECN1-HA). Cell lysates were immunoblotted for LATS1 and Beclin-1. Immunoblots represent three independent experiments. L.E. = long exposure; S.E. = short exposure.

(f) LATS1 does not affect Beclin-1 mRNA levels. HEK293T/17 cells were transfected with Flag-tagged Beclin-1 and vectors encoding empty vector (EV) or Myc-tagged LATS1, and RNA was extracted and analysed by quantitative RT-PCR (left side). Huh7 cells were transfected with control siRNA or siRNA against LATS1, and RNA was extracted and analysed by quantitative RT-PCR (right side). RPL19

mRNA expression was used as internal control. Results were pooled from three independent experiments. Statistical analysis was performed by two-tailed, unpaired t-test.

(**g**) Mapping of the LATS1 domain responsible for Beclin-1 stabilization. HEK293T/17 cells were transfected with a vector encoding Flag-tagged Beclin-1 (Flag-BECN1) and empty vector (EV) or vectors encoding various truncation mutants of LATS1 fused to GFP, as indicated and depicted in Figure 4a. Cell lysates were immunoblotted against Flag-Beclin-1 and GFP. Immunoblotting for GAPDH was used as loading control. Immunoblots represent three independent experiments.

(**h**) Determination of the binding capacity of the different truncation mutants of LATS1 to Beclin-1. HEK293T/17 cells were transfected with a vector encoding Myc-tagged Beclin-1 and empty vector (EV) or vectors encoding various HA-tagged truncation mutants of LATS1, as indicated and depicted in Figure 4a. Cell lysates were immunoprecipitated with antibodies against HA and then immunoblotted against Myc-Beclin-1 and HA-tagged LATS1 truncation mutants. Input represents immunoblotting of cell lysates before immunoprecipitation. Results represent three independent experiments.

(**i**) The kinase domain of LATS1 is not sufficient in regulating sorafenib-induced autophagy. Hep3B cells stably over-expressing wild-type full-length LATS1 or a ΔLCD1/2 mutant containing only the kinase domain were treated with DMSO vehicle control or sorafenib for 40 hours. Cell lysates were immunoblotted for p62, HA and LATS1. GAPDH serves as a loading control. Results represent three independent experiments.

Supplementary Figure 8, Tang et al.

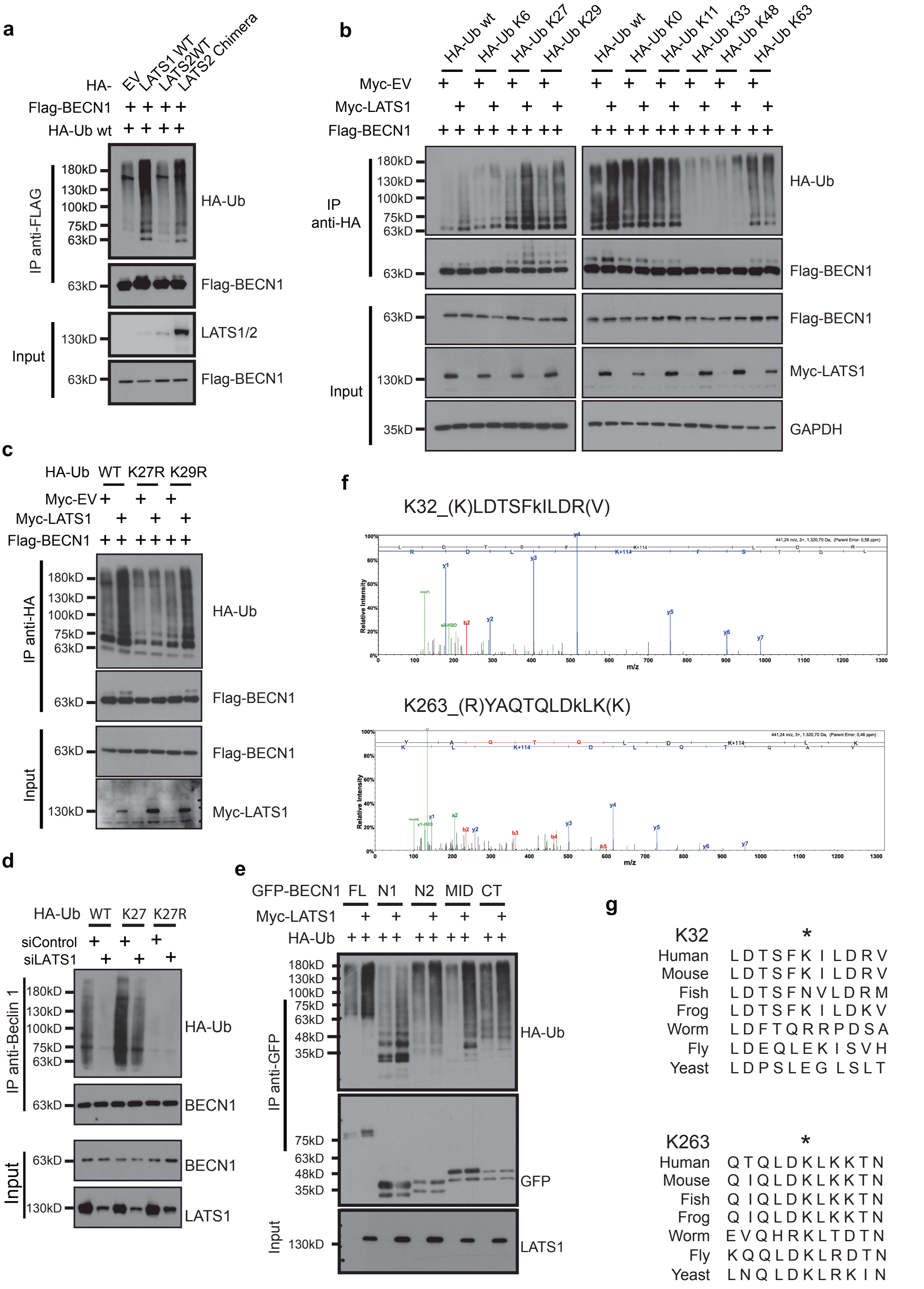

Suppl. Figure 8. LATS1 promotes K27-linked ubiquitylation of Beclin-1.

(a) LATS1, but not LATS2, enhances ubiquitylation of Beclin-1. HEK293T/17 cells were transfected with vectors encoding for Flag-tagged Beclin-1 (Flag-BECN1) and HA-tagged wild-type ubiquitin (HA-Ub wt) in addition to either empty vector (EV) or vectors encoding for HA-tagged LATS1, LATS2 or LATS2 chimera. 72 hours later, cell lysates were immunoprecipitated with anti-Flag antibodies and then immunoblotted for HA-ubiquitin and Flag-Beclin-1. Input represents immunoblotting of cell lysates for LATS1/2 and Flag-Beclin-1 before immunoprecipitation. Immunoblots represent three independent experiments.

(b) Characterization of linkage specificity in LATS1-induced ubiquitylation. HEK293T/17 cells were transfected with vectors encoding for Flag-tagged Beclin-1 (Flag-BECN1) and either empty vector (Myc-EV) or vectors encoding for Myc-tagged LATS1 and in addition vectors encoding for HA-tagged wild-type or various lysine (K) mutants of ubiquitin, as indicated. 72 hours later, cell lysates were immunoprecipitated with anti-HA antibodies and then immunoblotted for HA-ubiquitin and Flag-Beclin-1. Input represents immunoblotting of cell lysates for Flag-Beclin-1 and Myc-LATS1 before immunoprecipitation. Immunoblotting for GAPDH was used as loading control. Immunoblots represent three independent experiments.

(c) Validation of LATS1-induced K27-linked ubiquitylation of Beclin-1. HEK293T/17 cells were transfected with vectors encoding for Flag-tagged Beclin-1 (Flag-BECN1) and either empty vector (Myc-EV) or vectors encoding for Myc-tagged LATS1 and in addition vectors encoding for wild-type or K27R or K29R mutants of ubiquitin, as indicated. 72 hours later, cell lysates were immunoprecipitated with anti-HA antibodies and then immunoblotted for HA-ubiquitin and Flag-Beclin-1. Input represents immunoblotting of cell lysates for Flag-Beclin-1 and Myc-LATS1 before immunoprecipitation. Immunoblots represent three independent experiments.

(d) LATS1 induces Beclin-1 ubiquitylation at the endogenous level. HEK293T/17 cells were transfected with control siRNA or siRNA against LATS1 followed by transfection with HA-tagged wild type, K27 or K27R ubiquitin constructs. 72 hours later, cell lysates were prepared and immunoprecipitated with Beclin-1 antibody and immunoblotted with antibodies against Beclin-1, HA (HA-Ub). Immunoblots represent three independent experiments.

(e) Mapping the site of LATS1-induced Beclin-1 ubiquitylation. HEK293T/17 cells were transfected with vectors encoding for HA-tagged wild-type ubiquitin (HA-Ub) and Myc-tagged LATS1 and in addition vectors encoding various GFP-fused truncated fragments of Beclin-1. 72 hours later, cell lysates were immunoprecipitated with anti-GFP antibodies and then immunoblotted for HA-ubiquitin and GFP. Input represents immunoblotting of cell lysates for LATS1 before immunoprecipitation. Immunoblots represent three independent experiments.

(f) Mass spectrometry analysis of ubiquitylation sites in Beclin-1 induced by LATS1. Shown are representative results of ubiquitinated K32 and K263 peptides as identified by mass spectrometry.

(g) Evolutionary conservation of K32 and K263 across different species. K263, but not K32, is conserved across different species, indicating that LATS or LATS homolog-mediated ubiquitylation of K263 is highly conserved during evolution. Sequence alignment was achieved with uniprot access number Q14457 for Human, O8859 for Mouse, F1RCP1 for Fish, Q4A1L3 for Frog, Q22592 for Worm, Q9VCE1 for fly and A02948 for yeast.

Supplementary Figure 9, Tang et al.

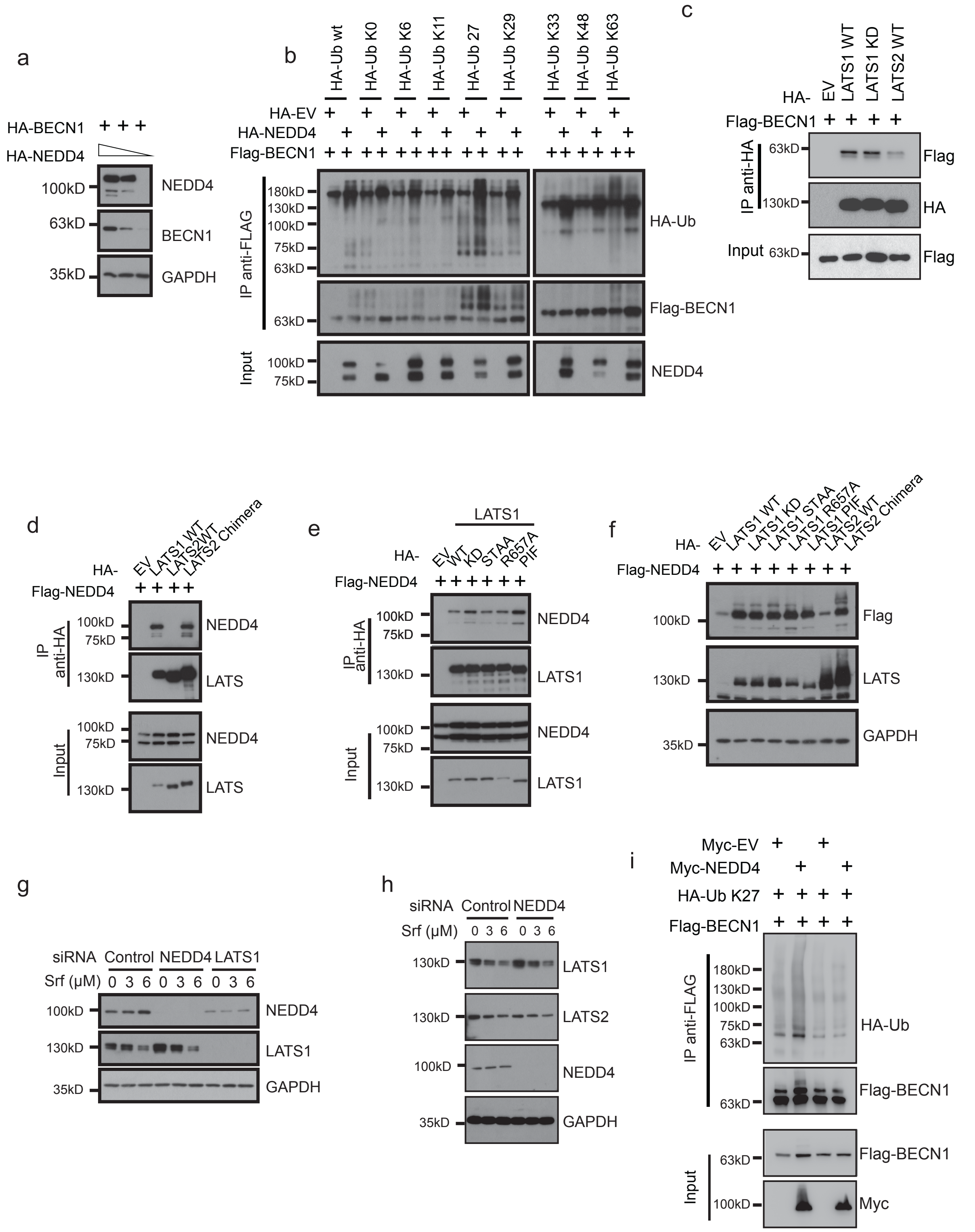

Suppl. Figure 9. NEDD4 as a potential E3 ligase in regulating LATS1-induced Beclin-1 ubiquitylation.

(**a**) NEDD4 stabilizes Beclin-1 in a dosage dependent manner. HEK293T/17 cells were transfected with vectors encoding for Flag-Beclin-1 (Flag-BECN1) and increasing plasmid concentrations of HA-tagged NEDD4 (HA-NEDD4). 72 hours later, cells were harvested and immunoblotted with antibodies against NEDD4 and Beclin-1, and GAPDH as loading control. Immunoblots represent three independent experiments.

(**b**) NEDD4 promotes K27-linked ubiquitylation of Beclin-1. HEK293T/17 cells were transfected with vectors encoding for Flag-tagged Beclin-1 (Flag-BECN1) and either empty vector (HA-EV) or vectors encoding for HA-tagged NEDD4 and in addition vectors encoding for HA-tagged wild-type or various lysine mutants of ubiquitin, as indicated. 72 hours later, cell lysates were immunoprecipitated with anti-HA antibodies and then immunoblotted for HA-ubiquitin and Flag-Beclin-1. Input represents immunoblotting of cell lysates for NEDD4 before immunoprecipitation. Immunoblots represent three independent experiments.

(**c**) LATS1 interacts with Beclin-1. HEK293T/17 cells were transfected with HA-tagged empty vectors (EV) or wild-type (WT) LATS1 or LATS2 or a kinase-dead (KD, D846A) mutant of LATS1with Flag-tagged Beclin-1 (Flag-BECN1), as indicated. Cells were harvested 72 hours later for immunoprecipitation with anti-Flag antibody and then immunoblotted for HA, Flag-tagged proteins. Input represents analysis of cell lysates before immunoprecipitation. Immunoblots represents three independent experiments.

(**d**) LATS1, but not LATS2 interacts with NEDD4. HEK293T/17 cells were transfected with vectors encoding Flag-tagged NEDD4 and with empty vector (EV) or vectors encoding for HA-tagged wild-type (WT) LATS1 or LATS2 or the LATS2 chimera as indicated. 72 hours later, cell lysates were prepared and immunoprecipitated with anti-HA antibody and immunoblotted with antibodies against NEDD4 and LATS1/2. Input represents analysis of cell lysates before immunoprecipitation. Immunoblots represent three independent experiments.

(e) LATS1 interacts with NEDD4 in a kinase activity-independent manner. HEK293T/17 cells were transfected with vectors encoding Flag-tagged NEDD4 and empty vector (EV) or HA-tagged wild-type or kinase-dead (KD-D846A, STAA, R657A) and a constitutive-active (PIF) mutant versions of LATS1 as indicated. 72 hours later, cells were harvested for immunoprecipitation with anti-HA antibodies followed by immunoblotting for LATS1 and NEDD4. Input represents analysis of cell lysates before immunoprecipitation. Immunoblots represent three independent experiments.

(**f**) LATS1, but not LATS2, stabilizes NEDD4 in a kinase activity-independent manner. HEK293T/17 cells were transfected with empty vector (EV) or vectors encoding for Flag-tagged NEDD4 and HA-tagged wild-type or kinase-dead (KD, STAA, R657A) and a constitutive-active (PIF) mutant versions of LATS1, LATS2 or LATS2 chimera, as indicated. Cells were harvested 72 hours after transfection

for immunoblotting analysis for Flag-NEDD4 and LATS1/2. GAPDH was used as loading control. Results represent three independent experiments.

(**g**) NEDD4 destabilizes LATS1, while LATS1 stabilizes NEDD4. Huh7 cells were transfected with siControl or siRNAs against NEDD4 or LATS1 and treated with sorafenib for 40 hours. Cell lysates were analyzed by immunoblotting for NEDD4 and LATS1. GAPDH was used as loading control. Results represents three independent experiments.

(**h**) NEDD4 destabilizes LATS1, but not LATS2. Huh7 cells were transfected with siControl or siRNAs against NEDD4 and treated with sorafenib for 40 hours. Cell lysates were analyzed by immunoblotting for NEDD4 and LATS1. GAPDH was used as loading control. Results represents three independent experiments.

(**i**) NEDD4 promotes Beclin-1 ubiquitination at lysine residues K32 and K263. Mutation of lysine residues K32/263 blocks NEDD4-induced K27-linked ubiquitination of Beclin-1. Beclin-1 lysine K32 and K263 were mutated to arginine via point mutagenesis. HEK293T/17 cells were transfected with HA-tagged ubiquitin, Flag-tagged wild-type or lysine-mutated K32/263R Beclin-1 (Flag-BECN1 WT or Flag-BECN1 2KR) and with vectors encoding for empty vector or Myc-tagged NEDD4 (Myc-NEDD4) as indicated. Cells were harvested 72 hours later for immunoprecipitation with anti-Flag antibody and then immunoblotted for HA, Flag and Myc tagged proteins. Input represents analysis of cell lysates before immunoprecipitation. Immunoblots represents three independent experiments.

Supplementary Figure 10, Tang et al.

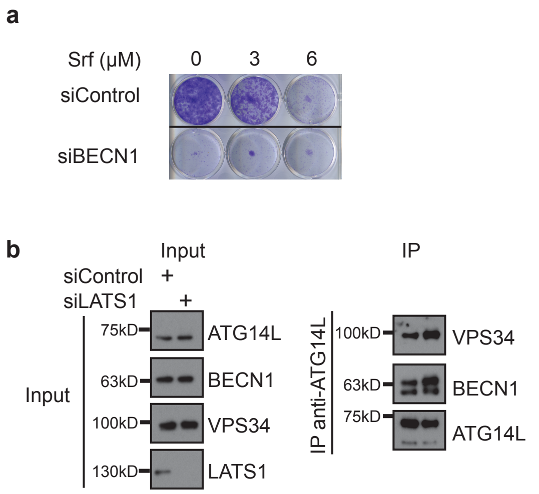

Suppl. Figure 10. A general role of Beclin-1 in cell viability and LATS1-mediated effects on Beclin-1 complex formation.

(**a**) siRNA-mediated loss of BECN1 expression results in impaired colony formation of HLE cells in response to increasing concentrations of sorafenib (Srf). HLE cells were seeded and transfected with either non-targeting control siRNA or siRNA targeting Beclin-1, and further treated as indicated for 10 to 14 days. The results represent three independent experiments.

(**b**) LATS1 inhibits formation of the ATG14L/Beclin-1/VPS34 autophagy complex. Huh7 cells were transfected with either non-targeting control siRNA or siRNA targeting LATS1. Cells were harvested 72 hours later for immunoprecipitation with anti-ATG14L antibody and then immunoblotted for ATG14L, VPS34 and Beclin-1. Input represents analysis of cell lysates before immunoprecipitation. Immunoblots represents three independent experiments.

Supplementary Figure 11, Tang et al

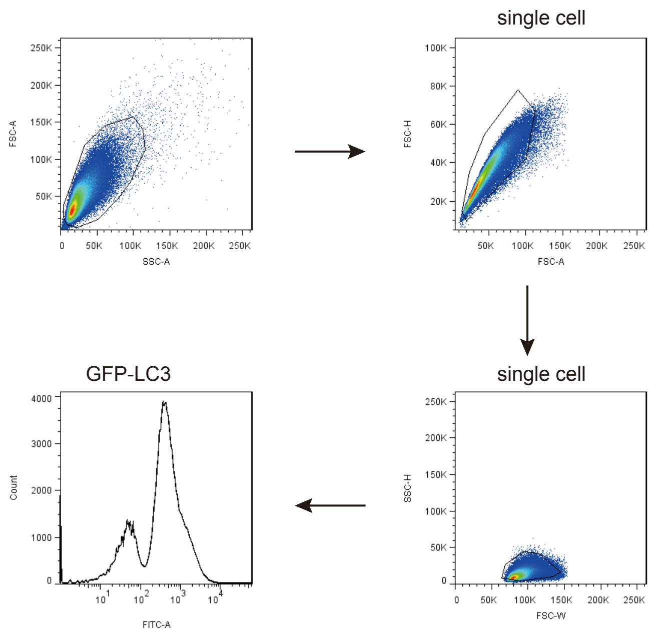

Suppl. Figure 11. Gating strategy.

Gating of the flow cytometry analysis of GFP-U2OS cells to determine LC3 intensity.

Supplemental Tables (attached as Excel Files)

Table SI: Description of siRNAs, qPCR primers, shRNAs and DNA plasmid constructs used in the study.

In this excel sheet, the sequences of the siRNAs, qPCR primers, shRNAs and descriptions of the DNA plasmid constructs used in the study are presented.

Table SII: Description of the antibodies used in the study.

In this excel sheet, the antibodies, the sources and suppliers and their dilutions used in the study are presented.
