# Supplementary material for: LATS1 but not LATS2 represses autophagy by a kinase-independent scaffold function
Source: Nat Commun. 2019 Dec 17;10:5755. doi: 10.1038/s41467-019-13591-7 (PMC6917744; doi:10.1038/s41467-019-13591-7)
Supplement: Supplementary file 2 — Description of Additonal Supplementary Files [file 41467_2019_13591_MOESM2_ESM.docx]

Description of Additional Supplementary Files

**File Name: Table SI: Description of siRNAs, qPCR primers, shRNAs and DNA plasmid constructs used in the study.**

In this excel sheet, the sequences of the siRNAs, qPCR primers, shRNAs and descriptions of the DNA plasmid constructs used in the study are presented.

**File Name: Table SII: Description of the antibodies used in the study.**

In this excel sheet, the antibodies, the sources and suppliers and their dilutions used in the study are presented
