# Supplementary material for: LATS1 but not LATS2 represses autophagy by a kinase-independent scaffold function
Source: Nat Commun. 2019 Dec 17;10:5755. doi: 10.1038/s41467-019-13591-7 (PMC6917744; doi:10.1038/s41467-019-13591-7)
Supplement: Supplementary file 5 — Reporting Summary [file 41467_2019_13591_MOESM5_ESM.pdf]

nature research

Corresponding author(s): Fengyuan Tang
Gerhard Christofori

Life Sciences Reporting Summary

Nature Research wishes to improve the reproducibility of the work that we publish. This form is intended for publication with all accepted life science papers and provides structure for consistency and transparency in reporting. Every life science submission will use this form; some list items might not apply to an individual manuscript, but all fields must be completed for clarity.

For further information on the points included in this form, see Reporting Life Sciences Research. For further information on Nature Research policies, including our data availability policy, see Authors & Referees and the Editorial Policy Checklist.

Please do not complete any field with "not applicable" or n/a. Refer to the help text for what text to use if an item is not relevant to your study. For final submission: please carefully check your responses for accuracy; you will not be able to make changes later.

Experimental design

1. Sample size

Describe how sample size was determined.

Page 13, in vivo tumor formation, Material and Methods. Sample sizes of mouse experiments, i.e. the number of mice per experimental cohort, were selected based on previous experience with the experimental systems to reach statistical significance.
All experiments have been repeated at least three times, as indicated in the Figure Legends.

2. Data exclusions

Describe any data exclusions.

No data has been excluded.

3. Replication

Describe the measures taken to verify the reproducibility of the experimental findings.

All molecular biology, biochemistry and cell biology experiments were repeated at least three times. Individual mouse experiments were not repeated, since the statistical evaluation has been built into the cohort sizes of the various treatment groups.

4. Randomization

Describe how samples/organisms/participants were allocated into experimental groups.

Mice were randomized into cohorts for treatment with placebo or with sorafenib.

5. Blinding

Describe whether the investigators were blinded to group allocation during data collection and/or analysis.

In all mouse experiments, blinding was performed at the level of analysis of tissue specimen. The investigators analyzing the material from were blinded in that they did not know the identity or the previous history of the specimen. The studies were not double-blinded, in that the investigators treating the mice were knowing which mouse is treated with which therapeutic regimen.

Note: all in vivo studies must report how sample size was determined and whether blinding and randomization were used.

6. Statistical parameters

For all figures and tables that use statistical methods, confirm that the following items are present in relevant figure legends (or in the Methods section if additional space is needed).

n/a	Confirmed	
☐	☒	The exact sample size (*n*) for each experimental group/condition, given as a discrete number and unit of measurement (animals, litters, cultures, etc.)
☐	☒	A description of how samples were collected, noting whether measurements were taken from distinct samples or whether the same sample was measured repeatedly
☐	☒	A statement indicating how many times each experiment was replicated
☐	☒	The statistical test(s) used and whether they are one- or two-sided<br>*Only common tests should be described solely by name; describe more complex techniques in the Methods section.*
☐	☒	A description of any assumptions or corrections, such as an adjustment for multiple comparisons
☐	☒	Test values indicating whether an effect is present<br>*Provide confidence intervals or give results of significance tests (e.g. P values) as exact values whenever appropriate and with effect sizes noted.*
☐	☒	A clear description of statistics including central tendency (e.g. median, mean) and variation (e.g. standard deviation, interquartile range)
☐	☒	Clearly defined error bars in all relevant figure captions (with explicit mention of central tendency and variation)

See the web collection on statistics for biologists for further resources and guidance.

Software

Policy information about availability of computer code

7. Software

Describe the software used to analyze the data in this study.	Image J, Vis software, IMARIS 8.2.1, Prizm statistic software, ComBat (PMID: 16632515), bioinformatical computations were performed with qCount function from QuasR package (version 3.12.1) and in R (R-3.3.1) version. For details see Material and Methods.

For manuscripts utilizing custom algorithms or software that are central to the paper but not yet described in the published literature, software must be made available to editors and reviewers upon request. We strongly encourage code deposition in a community repository (e.g. GitHub). *Nature Methods* guidance for providing algorithms and software for publication provides further information on this topic.

Materials and reagents

Policy information about availability of materials

8. Materials availability

Indicate whether there are restrictions on availability of unique materials or if these materials are only available for distribution by a third party.	All material used in the study is freely available upon request with the authors.

9. Antibodies

Describe the antibodies used and how they were validated for use in the system under study (i.e. assay and species).	Suppl. Table SII gives all information on the antibodies used in the study.

10. Eukaryotic cell lines

a. State the source of each eukaryotic cell line used.	Page 12, Material and Methods, Cell lines and cell culture.
b. Describe the method of cell line authentication used.	Purchased previously from AATC or shared by collaborators. Derivation of cell lines in house. Routine analysis of marker expression by RNA and protein analysis.
c. Report whether the cell lines were tested for mycoplasma contamination.	Yes, Page 12, Methods, cell lines and cell culture
d. If any of the cell lines used are listed in the database of commonly misidentified cell lines maintained by ICLAC, provide a scientific rationale for their use.	The cell lines used here are not specifically mentioned.

Animals and human research participants

Policy information about studies involving animals; when reporting animal research, follow the ARRIVE guidelines

11. Description of research animals

Provide all relevant details on animals and/or animal-derived materials used in the study.	Page 13, Material and Methods, tumor transplantation and sorafenib therapy study and autophagy study in vivo: All studies involving mice have been approved by the Swiss Federal Veterinary Office (SFVO) and the Cantonal Veterinary Office of Basel Stadt (license 2839).

Policy information about studies involving human research participants

12. Description of human research participants

Describe the covariate-relevant population characteristics of the human research participants.	Only established cell lines with human origin have been used. The studies have not directly involved patients or patient samples. RNA sequencing data has been acquired from publicly available databases.
