# Supplementary material for: LATS1 but not LATS2 represses autophagy by a kinase-independent scaffold function
Source: Nat Commun. 2019 Dec 17;10:5755. doi: 10.1038/s41467-019-13591-7 (PMC6917744; doi:10.1038/s41467-019-13591-7)
Supplement: Supplementary file 6 — Source Data [file 41467_2019_13591_MOESM6_ESM.pdf]

a

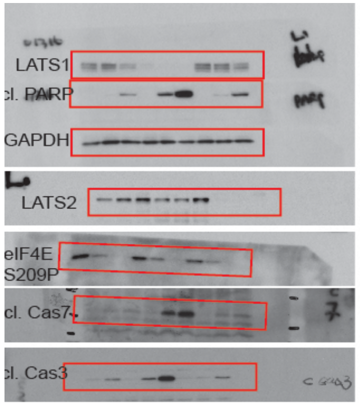

c

	171	390	397	398	399	422	423		
	shLacZ	shLacZ	shLacZ	shLacZ	shLacZ	shLacZ	shLacZ		
	34.4	32.8	30.4	27.4	30.6	27.5	29.8	average	SD
	4.5*4.6	4.7*8.6	5.3*3.3	2.9*4.5	2.4*3.7	3.3*4.4	5*6.5		
tumor size	46.575	94.987	28.8585	18.9225	11.232	23.958	81.25	43.6832857	32.4723851
	34.2	32.5	30.1	27.5	30.9	27.8	30.5		
	6.2*6.6	6.2*10.7	6.5*7.1	6.7*9.9	5.6*7.6	6.1*7.5	4.6*6.6		
tumor size	126.852	218.922	149.9875	222.2055	119.168	139.5375	69.828	149.500071	54.750316
	34.8	33	30	28	31	28.4	30		
	6.8*7.0	7.8*13.2	7.5*8.2	7.3*10.2	7.2*8.2	5.5*7.1	5.6*7.4		
tumor size	161.84	401.544	230.625	271.779	212.544	107.3875	116.032	214.535929	102.039441
	34.6	33	30.5	28	31.2	28	31.4		
	7.2*8.1	8.7*14	8.8*9.5	8.3*11.5	8.8*10.2	8.6*11.8	6.5*9.3		
tumor size	207.36	529.83	367.84	396.1175	394.944	436.364	196.4625	361.274	120.61843
	33.8	33.1	29	27.5	29.4	28.6	31.5		
	7.8*9.6	9.2*16.6	10*10.5	9.8*12.5	10.0*10.5	8.0*10.4	7.2*10		
tumor size	292.032	702.512	525	600.25	525	332.8	259.2	462.399143	169.11809
	35.1	34.1	31.2	28.9	31.5	28.4	30.7		
	10.9*11.9	10.8*17.1	12.2*12.2	12.0*14.4	12.6*14.1	12.8*12.6	10*10.8		
tumor size	706.9195	997.272	907.924	1036.8	1119.258	1016.064	540	903.4625	206.792953

	160	179	388	389	391	158	169	173	424		
	shLacZ	shLacZ	shLacZ	shLacZ	shLacZ	shLacZ	shLacZ	shLacZ	shLacZ		
	SRF	SRF	SRF	SRF	SRF	SRF	SRF	SRF	SRF		
	30	38.7	32.5	32.9	32	29.5	29.2	34.5	29.3		
	3.4*57	3.8*6.5	5*5.8	7*6.9	4*6.1	6.9*5.8	5.5*7.6	5.2*6.0	4.0*5.6	Average	SD
tumor size	32.946	46.93	72.5	166.635	48.8	116.058	114.95	81.12	44.8	80.5265556	44.1623397
	27.8	36.5	30.8	32.2	Dead 28-11	29	28.2	31	23.8		
	6.1*7.0	7.8*6.4	7.0*7.7	8.0*8.3	5.9*5.4	6.9*7.5	6.5*8.7	6.7*8.6	5.0*5.3		
tumor size	130.235	159.744	188.65	265.6	86.022	178.5375	183.7875	193.027	66.25	161.317	60.3165262
	27.4	37	32	32.2		28	28	29.5	23.2		
	8.2*8.9	7.0*9.8	7.5*6.8	8.3*9.0		7.0*8.0	7.5*9.5	5.2*7.5	5.5*7.1		
tumor size	299.218	240.1	173.4	310.005		196	267.1875	101.4	107.3875	211.83725	81.0371281
	27.8	35	32	32		26.5	26.8	30.8	22		
	8.0*10.0	7.5*104	9.3*9.5	8.6*9.5		6.8*7.9	7.5*9.7	6.0*8.8	6.0*7.2		
tumor size	320	292.5	410.8275	351.31		182.648	272.8125	158.4	129.6	264.76225	99.308668
	28	36	31.3	32.7		27.4	22.2	31.2	26		
	7.8*8.8	7.6*9.3	8.8*9.2	9.2*10.6		7.4*8.6	8.8*10.2	6.4*8.9	6.8*7.8		
tumor size	267.696	268.584	356.224	448.592		235.468	394.944	182.272	180.336	291.7645	98.6239202
	27.8	35.6	31.4	32.3		27.5	dead	31.1	26.4		
	8.4*10.5	9.2*8.8	8.3*9.4	9.8*10.6		7.6*7.8		7.6*7.9	7.2*9.3		
tumor size	370.44	356.224	323.783	509.012		225.264		231.04	241.056	322.402714	102.20629
	27.6	35.7	31.3	32.8		27.6		30.9	27.1		
	9.9*9.8	8.5*9.7	8.4*10.6	9.5*10.0		8.6*8.8		9.1*8.6	9.2*9.8		
tumor size	475.398	350.4125	373.968	451.25		325.424		336.518	414.736	389.672357	58.4666264
	27	34.5	30.9	32.4		28.3		31.1	27.5		
	9.0*10.2	9.8*9.8	9.8*10.8	10.5*10.5		9.8*10.8		10.2*10.2	9.5*9.8		
tumor size	413.1	470.596	518.616	578.8125		518.616		530.604	442.225	496.081357	56.9909224

	161	163	164	165	166	168	414	425		
	shLATS1	shLATS1	shLATS1	shLATS1	shLATS1	shLATS1	shLATS1	shLATS1		
	4.5*5.3	4.1*7.1	4.0*4.0	6.3*7.4	6.3*7.3	6.9*7.2	6.0*6.0	5.5*7.4	average	SD
tumor size	53.6625	59.6755	32	146.853	144.8685	171.396	108	111.925	103.547563	50.4388285
	41	33.4	37.2	33.4	30	36	30.4	31.9		
	5.7*4.5	7.4*8.0	4.0*4.1	7.4*8.0	7*7.4	6.8*7.5	6.2*6.8	6.9*8.5		
tumor size	57.7125	219.04	32.8	219.04	181.3	183.75	136.9305	202.3425	154.114438	72.3875865
	40.5	33.7	38.3	34	30	35.9	30.5	32.1		
	6.2*6.2	7.9*8	4.7*5	7.2*8.7	7.6*8.2	9.2*8.8	6.4*8.8	6.9*10.5		
tumor size	119.164	249.64	55.225	225.504	236.816	331.936	180.224	249.9525	206.057688	86.1594426
	40.1	33.6	38.3	34.6	30.4	35.5	30	31.8		
	6.4*6.6	8*9.4	5.3*5.6	7.8*8.6	9.5*10.5	8.8*9.2	8.8*7.2	8.6*9.9		
tumor size	135.168	300.8		261.612	473.8125	356.224	254.144	366.102	306.8375	106.583909
	38.8	32.4	38.2	34.4	30.2	33.8	30.4	32.6		
	7.5*8.8	8*10.7	4.6*5.5	9.3*10.3	8.5*10.1	10*9.7	8.7*11.5	9.3*11.0		
tumor size	247.5	342.4		445.4235	364.8625	470.45	435.2175	475.695	397.364071	83.4231375
	40.6	33.3	37.7	34.2	31	36.4	30.5	34		
	9.4*9.4	10.2*12.4	5.9*6	13.7*13.5	11*10.3	13.6*11.5	11.7*14.2	11.7*13.2		
tumor size	415.292	645.048		1248.4125	583.495	899.3	971.919	903.474	809.562929	279.714708

	162	167	400	407	408	412	413	416	426		
	shLATS1	shLATS1	shLATS1	shLATS1	shLATS1	shLATS1	shLATS1	shLATS1	shLATS1		
	SRF	SRF	SRF	SRF	SRF	SRF	SRF	SRF	SRF		
	34.9	34.6	31	31.5	32.2	30.8	31.2	31.7	29		
	6.2*5.1	5.0*7	7.3*8.1	5.0*6.7	5.5*5.6	5.2*6.6	5.2*6	5.7*6.7	6.7*7.5	average	SD
tumor size	80.631	87.5	215.8245	83.75	84.7	59.488	81.12	108.8415	168.3375	107.799167	50.7977547
	31.9	32	28.7	Dead 28-11	30.1	28.4	29.6	28.9	26.4		
	5.3*5.1	6.4*8.4	5.5*7.1	7.8*7.7	4.9*5.0	4.9*6	4.4*4.8	5.2*7.2	5.3*7.8		
tumor size	68.9265	172.032	107.3875	231.231	60.025	72.03	46.464	97.344	109.551	107.221222	59.4828844
	29	32.7	26.8		30.5	28.8	29.4	26.7	27		
	5.5*5.6	5.2*6.3	6.1*7.4		6.6*7.7	5.4*6	5.6*5.7	7.0*6.7	6.8*9.2		
tumor size	83.16	85.176	137.677		167.706	87.48	89.376	157.115	212.704	127.54925	48.7987713
	27.9	33	25.1		29.2	29.6	24	27	27.3		
	5.7*6.5	7.0*9.3	6.4*8		6.0*7.5	5.8*6.8	4.8*5.3	7.8*8.0	7.2*10.5		
tumor size	105.5925	227.85	163.84		135	114.376	61.056	243.36	272.16	165.404313	74.9953438
	28.9	29.2	26		29.4	28.7		26.6	28		
	5.6*6.0	6.2*6.9	6.2*7.8		6.7*7.2	6.2*6.0		5.9*8.7	8.0*8.8		
tumor size	94.08	132.618	149.916		161.604	111.6		151.4235	281.6	154.691643	60.8343461
	30.2	29.7	27.8		29.8	28.6		29.3	27.5		
	6.2*6.8	7*8.2	7.2*9.2		7.2*8.6	6.8*7.5		7*8.8	7.3*9.2		
tumor size	130.696	200.9	238.464		222.912	173.4		215.6	245.134	203.872286	40.1898189
	30.6	30.1	27.6		29.6	28.4		29	27.2		
	6.2*5.5	6.3*7.8	7.8*8		7.2*7.2	6.9*8.8		7*8.8	7.9*9.9		
tumor size	93.775	154.791	243.36		186.624	209.484		215.6	308.9295	201.794786	67.7778291
	29.4	31.8	27.7		28.7	28.2		28.6	27.3		
	6.8*6.6	7.8*8.4	8.6*10.6		8*8	6.6*7.4		8.3*9.3	8.2*9.3		
tumor size	148.104	255.528	391.988		256	161.172		320.3385	312.666	263.685214	87.6202943

d

1C	DMSO	100	100	100		
	Srf 4um	69.9609259	56.8	51.5		
1L	DMSO	100	100	100		
	Srf 4Um	57.4462998	41.3	38.3		
6C	DMSO	100	100	100		
	Srf 2um	78.3	82.8	64.9		
6L	DMSO	100	100	100		
	Srf 2um	56.5	66.8	51.8		
3C	DMSO	100	100	100	100	100
	Srf 2um	87.8	57.1	46.0	108.5	52.3
3L	DMSO	100	100	100	100	100
	Srf 2um	57.2	33.3	33.4	48.8	47.3

f

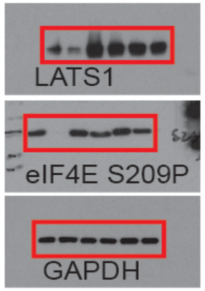

l

Pre-Sorafenib			On- srafenib
Responder	Non-responsder	Responder	Non-responsder
0.349	0.233	0.077	0.28
-0.028	0.024	-0.064	0.238
-0.019	1.249	0.107	1.108
	1.466	0.015	1.033
	-0.066		-0.043
	0.584		0.688

Figure 1

b

siContro DMSO	2.78	3.11	1.67	1.65	2.21	4.75	6.4	12.08	3.14	4.71	2.52	2.43	3.88	2.54	1.33	2.13	2.06	2.86	2.03	3.27	2.09	2.56	1.53	1.32	1.88
siLATS1 DMSO	9	4.88	8.24	7.38	8.2	2.81	3.59	5.74	8.08	10.44	1.47	7.77	2.1	2.03	4.9	7.52	9.26	2.19	2.69	3	2.53	5.91	2.9	18.29	7.77
siLATS2 DMSO	3	8.38	2.9	2.25	1.33	3.45	4.64	6.64	5.92	2.59	2.76	6.42	3.87	2.67	4.3	1.71	2.15	2.71	3.9	1.65	2.1	5.29	3.04	4.14	5.19
siContro Baf	2.31	3.52	3.86	5.63	3.14	3.22	2.72	2.68	6.09	2.14	3.5	2.52	3.09	2.32	2.2	2.61	4.53	2.21	2.27	3.17	4.52	4.75	2.11	5.09	3.06
siLATS1 Baf	4.58	9.27	7	13.73	4.74	4.84	17.29	11.05	4.36	13.04	2.69	3	5.01	5.2	9.6	4.54	2.82	3.21	3.92	4.84	9.67	8.92	9.6	12.56	9.86
siLATS2 Baf	9.65	6.08	4.16	4.72	5.43	6.5	7.3	7.63	6.33	4.74	3.83	3.67	4.17	3.8	7.11	4.7	6.48	3.78	6.45	2.77	6.5	3.96	4.63	6.43	5.14
siContro Srf	8.56	5.69	2.89	9.47	6.21	6.76	5.85	12.25	4.6	5.74	8.63	4.14	1.57	4.8	5.03	2.33	7.61	4.33	1.58	5.13					
siLATS1 Srf	10.6	29.27	14.88	15.53	15.13	17.65	8.63	7.19	12.43	30.9	8.48	9.86	4.81	8.42	6.41	8.67	13.43	8.53	8.05	6.85					
siLATS2 Srf	7	9.55	6.21	8.34	7.82	10.59	18.39	10.4	14.55	5	7.03	2.56	5.76	5.2	12.78	4.07	12.71	7.41	10.82	8.07					
siContro Baf+Srf	16.47	9.24	15.5	36.2	20.83	7.17	6.93	10.03	27.59	12.58	4.89	8.45	6.68	4.79	8.55	16.76	13.72	10.64	6.38	5.88	18.17	21.74	18.29	20.22	18.17
siLATS1 Baf+Srf	25	23.76	21.64	26.71	50.93	13.59	18.18	19.82	16.51	13.29	11.04	11.46	11.02	12.65	11	10.41	11.99	10.8	15.66	11.61	21.31	20.37	28	28.96	20
siLATS2 Baf+Srf	19.94	8.83	14.71	22.72	9.04	26.27	2.22	3.96	1.83	4.4	15.83	14.98	4.56	10.84	11.96	10.93	5.32	6.05	6.3	6.83	15.23	8.51	15.15	25.88	11.59

c

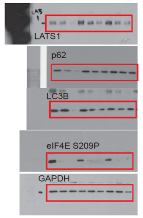

d

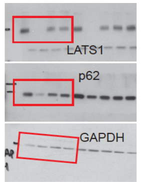

e

	exp 1	exp 2	exp 3	avergae	SD	t test	t test siLATS1
siCtrl	1	1	1	1	0		
siLATS1	0.241	0.22	0.12	0.1959	0.0637	0.0021	
siLATS1+WT	0.874	0.94	0.64	0.8204	0.1561	0.1844	0.008295466
siLATS1+KD	0.694	0.92	0.78	0.8	0.1142	0.0936	0.015604461

g

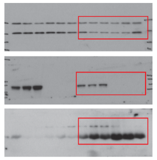

h

	WT	KO
	0.6788	1.9897
	1.2046	2.053
	1.1166	1.5774
average	1	1.8734
SD	0.2816	0.2583
t test		0.0167

i

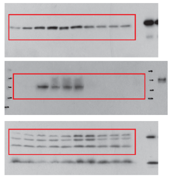

j

	WT	KO
	0.9588	0.5552
	0.8888	0.3957
	1.0248	0.4836
	1.1276	0.4675
		0.5213
		0.47
average	1	0.4822
SD	0.1016	0.0543

Figure 2

a

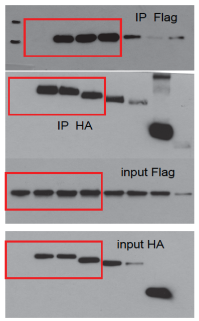

b

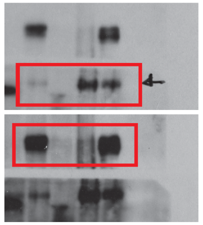

C

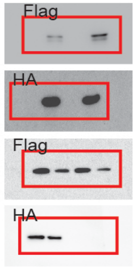

d

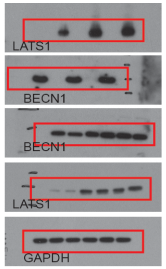

e

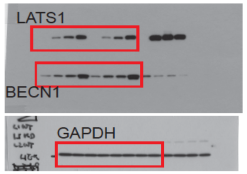

f

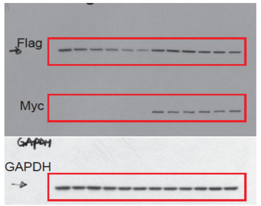

g

exp 1	exp 2	exp 3
100	100	100
93.5583255	85.3460185	77.7408093
70.2943072	80.7813775	62.3667525
67.514569	61.5843922	56.9184583
56.2353531	41.1640569	49.0515213
37.9092655	23.9657064	33.6579567
100	100	100
100.214368	97.3151117	96.771229
98.0297134	96.3419196	87.326775
85.4949083	84.0635809	79.9338573
82.2220877	73.0928574	71.657156
75.4715954	58.7207911	65.2682281

h

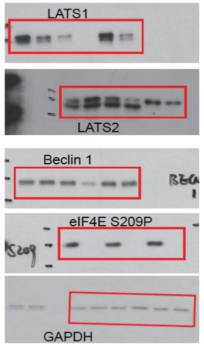

i

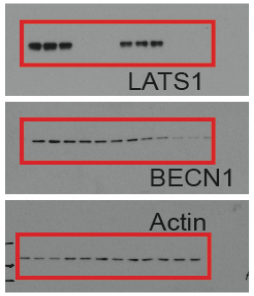

Figure 3

c

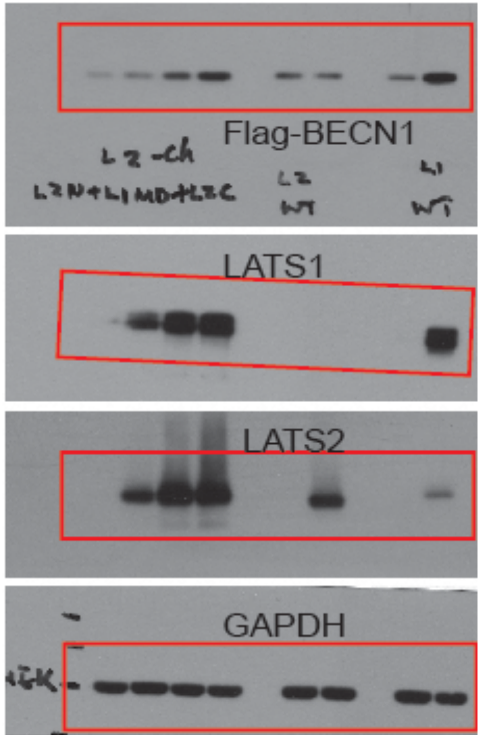

d

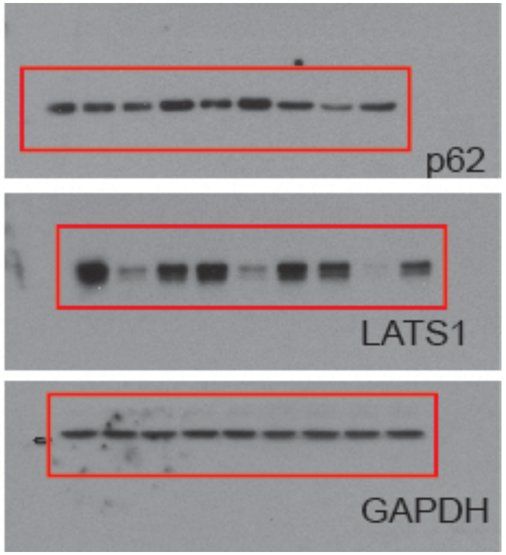

e

	siControl	siLATS1	siLATS1+CHL2
exp 1	1	0.3412831	0.92348745
exp 2	1	0.42323212	0.8624361
exp 3	1	0.33421742	0.76984885

Figure 4

a

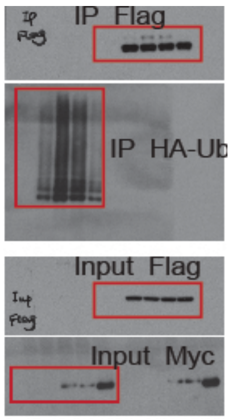

b

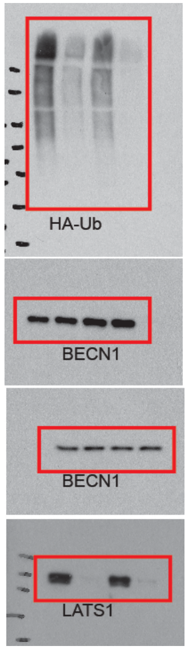

c

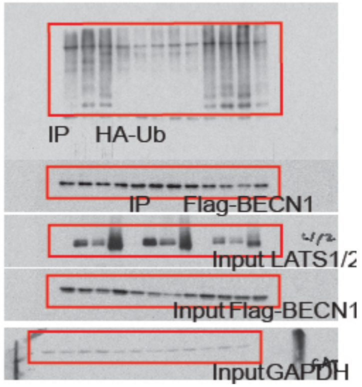

f

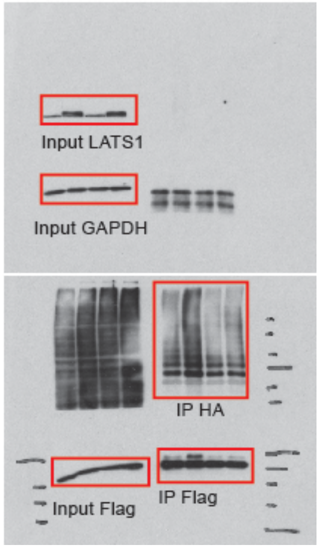

e

EV	2.865793	1.281593	1.601436	1.381172	1.569902	1.870545
LATS1	0.918948	1.370782	1.616632	1.651881	2.49501	1.70351

Figure 5

a

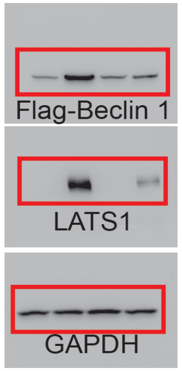

b

	exp 1	exp 2	exp 3
BECN1 WT+ EV	1	1	1
BECN1 WT+ LATS1	3.380406	3.910084	4.598283
BECN1 2KR+ EV	1.027343	1.250801	1.098519
BECN1 2KR+ LATS1	1.733343	1.98497	1.959859

c

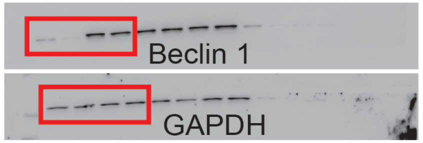

e

DMSO		Sorafenib		CQ		Sorafenib+CQ	
wt	kr	wt	kr	wt	kr	wt	kr
1.86	4.21	10.32	18.98	16.87	20.98	20.98	32.09
2.97	5.67	8.76	13.21	17.32	28.98	14.89	32.09
1.67	9.21	9.87	8.42	10.98	19.87	19.87	18.98
1.65	3.21	5.66	7.46	8.98	21.9	22.09	40.98
4.76	7.83	7.34	8.98	12.09	18.34	8.04	28.98
5.87	9.23	4.23	8.05	10.98	16.87	8.39	16.43
6.24	8.64	8.78	17.86	8.98	13.89	12.98	25.98
5.15	4.32	9.54	9.98	9.78	9.89	21.09	35.98
7.12	3.25	6.87	13.87	6.87	8.04	14.98	20.98
5.12	6.98	4.35	18.98	10.98	28.98	8.65	19.67
4.13	1.47	8.23	9.87	15.36	18.98	10.98	14.98
6.21	6.23	4.56	9.78	9.87	18.9	9.87	10.786
8.92	8.21	6.43	15.46	10.98	18.09	7.98	29.98
1.72	3.24	6.98	4.56	11.465	9.87	9.88	30.98
1.23	8.53	3.68	9.53	9.65	8.68	18.97	17.98
4.21	9.23	8.42	10.98	8.45	14.98	28.9	39.09
3.24	4.87	6.86	9.87	8.34	19.63	18.98	16.98
5.12	5.78	9.23	6.98	9.87	16.98	16.98	25.09
3.15	8.09	2.34	5.98			18.41	21.09
1.87	4.21	5.46	8.98			18.4	28.98
2.14	1.54	7.23	12.87			20.98	30.98
2.41	7.32	5.78	8.92			18.29	32.08
5.24	6.42	12.11	9.6				
6.23	8.98	9.65	12.56				
1.87	9.76	3.98	13.98				

g

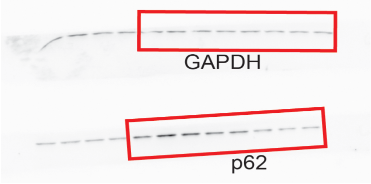

h

	exp 1	exp 2	exp 3
siBECN1+WT	96.4474	93.72872	109.8239
siBECN1+2KR	76.30545	68.52691	61.18706
siBECN1+WT SRF	98.61081	81.76478	85.65538
siBECN1+2KR SRF	28.73069	35.37548	46.13955

i

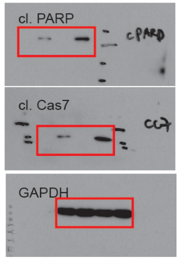

Figure 6

a

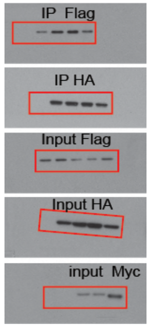

b

exp 1	exp 2	exp 3	exp 4
1	1	1	1
1.6731201	1.57103947	1.47485832	1.5559748
1.58249159	1.70605166	1.61412876	
0.85826447	0.93677068	1.00523724	1.02714806

c

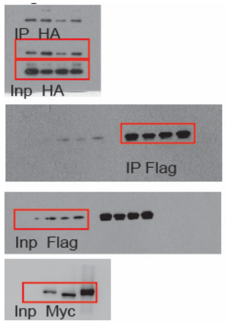

d

	exp 1	exp 2	exp 3
EV	1	1	1
L1 WT	1.86293038	1.31658757	1.65397971
L2WT	0.80645161	0.86979871	1.06451781
L2 CHR	1.44429083	1.35151086	1.88373551

e

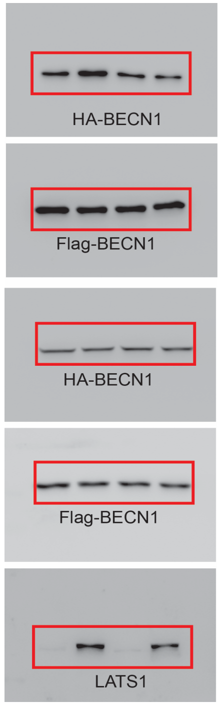

f

	exp 1	exp 2	exp 3
WT/EV	1	1	1
WT/LATS1	1.75072	1.63031948	1.37582921
KR/EV	1.06378667	1.13679536	0.99758775
KR/LATS1	0.94762667	0.8702356	0.98854179

Figure 7

a

	0	3	6	9
siControl	100	78.0319672	61.4881396	40.3796993
	100	82.5337799	66.7023379	43.0471539
	100	86.029569	60.9182504	39.0401836
Ave	100	82.1984387	63.0362426	40.8223456
SD	1.0049E-14	4.00933271	3.18769264	2.03982955
siLATS1	100	78.7963529	41.1276537	27.2161817
	100	61.6448947	38.5264741	19.6904631
	100	66.5733839	30.5693482	21.7277386
Ave	100	69.0048772	36.7411587	22.8781278
SD	0	8.8304728	5.50090605	3.89251287
siLATS2	100	78.5301786	56.6695932	38.2652724
	100	85.9149512	66.6964271	47.4134004
	100	78.3407922	67.3017348	43.9978274
Ave	100	80.9286406	63.5559183	43.2255
SD	0	4.31930969	5.97140728	4.62270794

b

SNU423		0	2	4	6
	siControl	100	92.9679282	76.1454631	40.1488842
		100	94.1660203	86.165398	42.6734887
		100	97.5205231	63.9620728	44.845432
	average	100	94.8848239	75.4243113	42.555935
	SD	0	2.35988117	11.1192157	2.35047961
	siLATS1	100	75.6539701	37.4264027	18.9308312
		100	58.5719439	50.2192904	38.1685509
		100	79.4804942	52.370289	30.572791
	average	100	71.2354694	46.671994	29.2240577
	SD	0	11.1325752	8.07882527	9.68951886
	siLATS2	100	89.6222658	64.4228772	36.8213148
		100	92.5023748	77.1088523	44.0730111
		100	97.953957	64.9389034	49.6470932
	Average	100	93.3595325	68.8235443	43.5138064
	SD	0	4.23146656	7.17992456	6.43114919

c

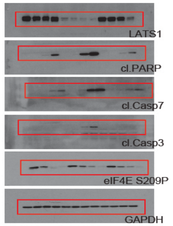

e

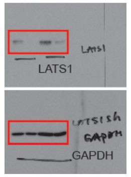

f

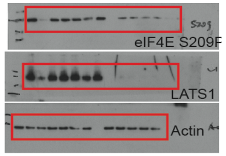

g

Huh7 DMSO

Srf concentration	exp 1	exp 2	exp 3
0	100	100	100
0.5	115.949203	34.4030309	95.1075
2	62.7867804	22.6082813	43.49511
5	21.40168	15.4071506	17.64536
10	14.527504	14.6977461	13.36747
20	11.2147629	12.0607715	11.60486

Huh7 I.R.

Srf concentration	exp 1	exp 2	exp 3	exp 4
0	100	100	100	100
0.5	166.937778	126.963757	97.5350775	97.48668
2	136.555152	109.039807	99.8070484	93.18535
5	85.8138059	75.8271561	74.4171587	81.31033
10	34.8620359	41.2555779	50.8962138	42.96831
20	29.8015964	43.797446	27.5378278	30.98063

Huh7 C.R.

Srf concentration	exp 1	exp 2	exp 3
0	100	100	100
0.5	122.546835	115.302614	98.8777463
2	100.51443	100.676858	91.9837202
5	86.827296	66.666262	62.6784958
10	75.5662794	22.4655111	38.2421719
20	43.6307734	11.9699637	23.0883645

k

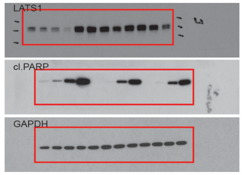

l

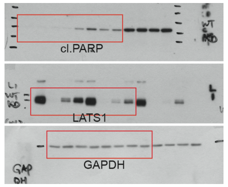

m

	si144/EV		siLATS1/EV		siLATS1 WT		siLATS1/KD	
	DMSO	Srf	DMSO	Srf	DMSO	Srf	DMSO	Srf
	100	75.9886759	100	56.5899916	100	69.0923396	100	66.9569667
	100	67.6280712	100	51.3623471	100	63.2352925	100	63.8265404
	100	70.098968	100	61.717589	100	69.9905082	100	66.785639
average	100	71.2385717	100	56.5566426	100	67.4393801	100	65.856382
SD	0	4.29522414	0	5.17770149	0	3.6684385	0	1.75998042

n

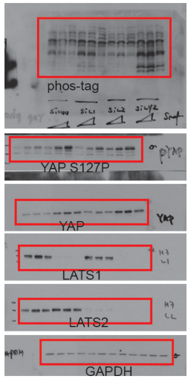

Suppl. Figure 1

b

SNU423

DMSO			Sorafenib			Baf			Sorafenib+Baf		
siContorl	siLATS1	siLATS2	siContorl	siLATS1	siLATS2	siContorl	siLATS1	siLATS2	siContorl	siLATS1	siLATS2
2.16	12.18	4.48	8.18	11.71	9.29	20.57	15.79	12.6	20.5	33.86	21.77
4.9	7.35	6	11.42	13.59	10.17	13.94	14.71	9.29	29.23	33.44	24.81
4.89	12.9	5.5	15.17	12.53	7.57	6.36	16.75	9.43	14.59	48.14	24.32
6.75	9.03	5.64	13.35	11.83	7.21	8.89	14.15	11.6	12.35	30.38	22.51
2.92	16.82	6.85	6.37	12.41	4.31	9.88	15.46	13.82	13.34	33.97	13.26
3.95	13.25	8.07	7.89	15.06	6.79	5	17.71	7.14	31.11	26.58	23.82
4.69	6.05	9.21	8.61	12.05	7.14	18.68	23.75	10.69	25.7	27.1	17
7.53	6.29	6.74	8.21	12.96	11.14	10.36	16.65	11.15	30.63	22.09	33.3
3.61	7.67	5.48	7.78	18.06	11.52	6.33	14.63	5.05	15.43	35.23	19.51
4.83	8.71	9.5	5.87	18.44	6.5	6	15.67	11.9			

c

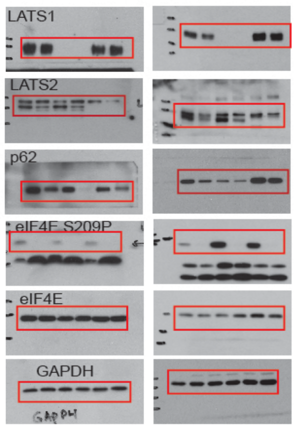

d

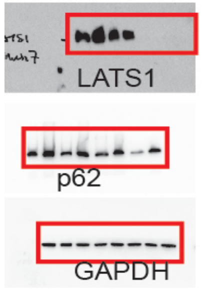

e

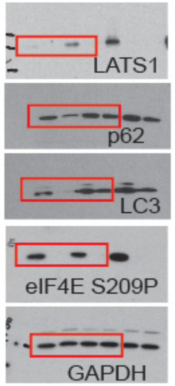

f

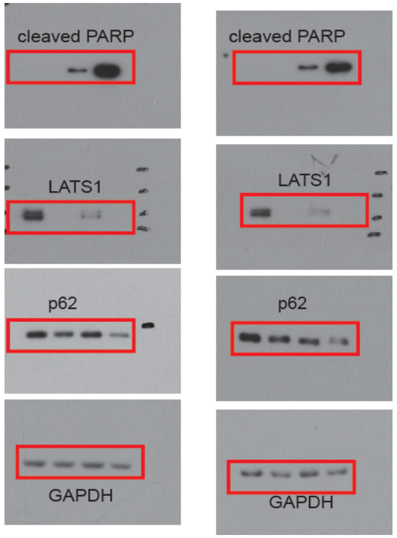

g

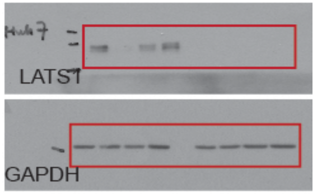

Suppl. Figure 3

a

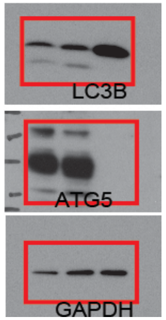

b

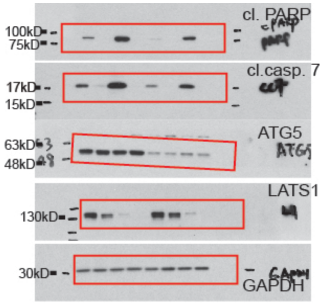

c

		DMSO	SRF			DMSO	SRF			DMSO	SRF			DMSO	SRF
siControl	exp 1	100	65.9230077	siLATS1	exp 1	100	41.89	siATG5	exp 1	100	79.01	siATG5/LATS	exp 1	100	56.11
	exp 2	100	60.92		exp 2	100	33.45		exp 2	100	79.5		exp 2	100	60.11
	exp 3	100	63.96		exp 3	100	35.39		exp 3	100	72.74		exp 3	100	51.04
	Ave	100	63.6010026		Ave	100	36.91		Ave	100	77.0833333		Ave	100	55.7533333
	SD	1.0049E-14	2.52075008		SD	0	4.42054295		SD	0	3.76940756		SD	1.0049E-14	4.54550694

d

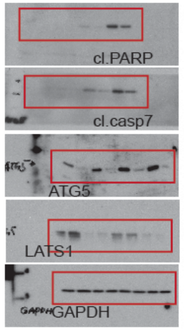

e

		Srf 0	Srf 1	Srf 2	Srf 0 CQ	Srf 1 CQ	Srf 2 CQ
shControl	exp 1	3.748	2.808	0.413	3.455	2.279	0.925
	exp 2	3.781	2.374	0.844	3.804	2.488	0.347
	exp 3	3.747	2.428	0.588	3.635	1.595	0.54

		Srf 0	Srf 1	Srf 2	Srf 0 CQ	Srf 1 CQ	Srf 2 CQ
shLATS1	exp 1	3.729	0.08	0.05	3.804	1.345	0.126
	exp 2	3.758	0.201	0.058	3.825	1.524	0.2
	exp 3	3.75	0.343	0.069	3.773	1.162	0.231

Suppl. Figure 4

g

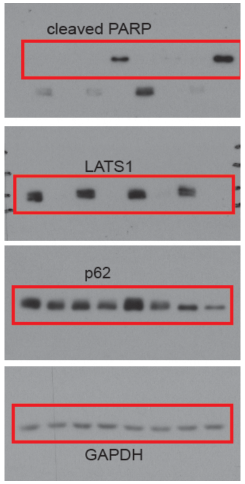

b

A2058

DMSO

siContorl	siLATS1	siLATS2
6.3	6.7	9.3
6.5	6.9	8
4.5	6.6	6.4
5.7	7.5	7.2
6	7.2	9.7
6	9.5	7.5
5.3	10.3	7.7
4.8	7.7	6.9
5	11.8	7.6

PLX

siContorl	siLATS1	siLATS2
9.8	15.9	
9.3	10.8	6.4
10	13.9	7
6.7	11.9	6.2
7.7	10.2	10.7
6.6	9.6	10.9
5.9	10.3	8.7
8.6	10.5	7.2
9.2	10.4	7.6
	8.7	8.9
		9.3
		8.2

Baf

siContorl	siLATS1	siLATS2
15.9	22.7	23.4
18.3	24	29.2
18.3	35.6	27.6
13.9	28.4	22.6
20.7	29.2	21.9
18.3	38.3	22.5
19.9	28.1	27.8
19.7	25.3	25.8
23.8	27.3	22.7
22.4	29.1	19.8
		19.5

PLX+Baf

siContorl	siLATS1	siLATS2
21.2	29.1	25.1
20.6	24.9	21.3
23.3	23.8	26.6
21.2	39.9	17.6
27.6	32.5	17.4
25.9	46.3	21.9
14.2	27.9	19.3
15	37.6	17.5
23	28.9	22.9
25.5	38.4	10.3
	23.8	

c

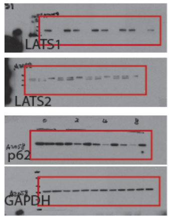

d

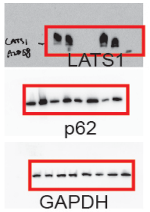

f

siControl

untreated	average	SD	CQ	average	SD	Rapmycin	average	SD	Rapamycin+CQ	average	SD
11.0434783	10.2347459	1.39175792	15.2380952	16.5037327	2.74927549	17.7179487	17.4713956	3.06694615	31.0714286	29.4795799	3.90314824
9.63157895			17.6666667			17.0384615			35.3		
10.5714286			22.7619048			14.3448276			31.2962963		
10.0333333			17.3809524			15.5555556			32.0909091		
9.75			15.2631579			11.4117647			25.5		
8.77777778			17.7647059			18.0465116			25		
10.1944444			12.137931			20.6206897			28.3666667		
9.41176471			15.5			18.2894737			32.7222222		
10.3142857			15.15			20.3103448			30.4482759		
9.38709677			16.173913			21.3783784			23		
14											
11.3809524											
8.55555556											

siLATS2

untreated	average	SD	CQ	average	SD	Rapmycin	average	SD	Rapamycin+CQ	average	SD
9.38709677	12.6445672	3.31056481	24.1	18.3115131	3.4031168	25.4444444	19.1811685	4.24076073	31.8125	31.3703521	6.93285231
11.7073171			17.1538462			18.3684211			27.1111111		
12.4594595			17.4705882			27.8			33.5625		
9			11.15			21.68			29		
12.5789474			20.3076923			17.2272727			33.8125		
12.5652174			18.625			17.8333333			48.6923077		
8.85714286			18.3125			15.5925926			32.2666667		
19.4375			19.3636364			19.7083333			25.2121212		
15.2307692			20.7857143			17.7619048			25		
15.2222222			15.8461538			14.68			23.9166667		
						14.8965517			34.6875		

siLATS1

untreated	average	SD	CQ	average	SD	Rapmycin	average	SD	Rapamycin+CQ	average	SD
20.2173913	15.4884761	2.4284264	21.2962963	25.5045175	4.41619914	28	26.117763	5.42486926			
16.0344828			32.25			22.7368421			47.4705882	43.84208	6.97687473
14.7777778			28.9230769			21.6111111			42.0833333		
13.8571429			21.8571429			23.6666667			34.1923077		
11.6060606			27.2083333			28.8			50.52		
15.2777778			22.3636364			23.7142857			39.2631579		
18.3214286			32.7			24.9285714			50.5384615		
14.8571429			24.48			31.625			37.6521739		
17.6190476			21.1333333			16			52.75		
13.7727273			21.2173913			37.6086957			43.9428571		
14.0322581			23			27.6875			33.5		
			29.625			27.0344828			50.35		

h

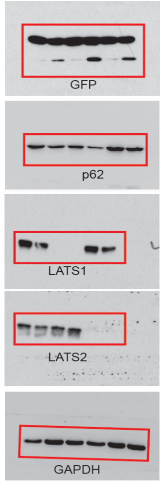

Suppl. Figure 5

c

siControl Vehnicle

	yellow	red
	4.75	10.5
	5.8	6.8
	6.33333333	7.66666667
	5.25	9.75
	4.16666667	10.3333333
	4.5	9.5
	5.8	9.2
	8.66666667	13.6666667
	7.33333333	12.6666667
	7.25	10.25
	6.16666667	8.5
	6.4	12.2
	5.5	10.6666667
	5.4	10.8
	5.75	8.25
average	5.93777778	10.05
SD	1.13080546	1.80398735

siControl CQ

	yellow	red
	9.66666667	1.66666667
	11.5	1.33333333
	12.4	1.2
	8	1.25
	10.6666667	2.66666667
	9.16666667	2.16666667
	10.4	1.2
	8.5	1.25
	6.4	1.8
	9.75	2.25
	13.6666667	2.33333333
	12.2	0.6
	9.75	1
	12.6666667	1.33333333
	11.2	1.2
	14.6666667	2.33333333
average	10.6625	1.59895833
SD	2.16557665	0.59040064

siControl rapa

	yellow	red
	2.25	16.75
	2.66666667	15.1666667
	4.33333333	21.6666667
	2.83333333	16.1666667
	2.4	15.6
	3.66666667	22.3333333
	1.6	16.2
	3.33333333	15.1666667
	2.33333333	15.6666667
	3.16666667	13.6666667
	3.75	17.25
	3.66666667	14.6666667
	3.83333333	13.5
	3.8	12.2
	3.25	17.25
	3.8	16.6
	2.75	20.25
average	3.14313725	16.4764706
SD	0.73250566	2.73989975

siControl rapa+CQ

	yellow	red
	24.6	2.2
	31.3333333	4.33333333
	28	2.25
	32.3333333	5.33333333
	20.1666667	2.83333333
	35.75	2.25
	36.5	3.5
	32.4	3.4
	24.5	4.5
	26	5.25
	18.1666667	1.83333333
	21.5	3.25
	30.3333333	3.33333333
	23.5	3.25
	21.8333333	3.16666667
	35.25	4.5
	30.6666667	3.66666667
average	27.8137255	3.46176471
SD	5.7688978	1.04379391

siLATS Vehnicle

	yellow	red
	10.6666667	17.6666667
	11.6666667	13.6666667
	8.6	14.2
	3.5	11.8333333
	7.75	17.25
	7.25	12.25
	11.3333333	17
	9.83333333	11.1666667
	12.2	14.2
	9.8	11.2
	10.3333333	17.3333333
	8.5	11.8333333
	6.33333333	17.3333333
	4.6	14.2
	11.8333333	8.16666667
	6.2	16.2
average	8.775	14.09375
SD	2.56782306	2.76706746

siLATS1 CQ

	yellow	red
	15.75	2.25
	26.3333333	2.33333333
	18.25	2.25
	15.3333333	2.5
	16.2	2.8
	20.5	3.5
	13	2.25
	18.2	2.6
	23.6666667	3.66666667
	20.4	3.2
	15.5	2.25
	15.25	2.75
	12.8	2.6
	15.6666667	3.16666667
	14	2.25
	14.6666667	2.33333333
	18.2	2.2
average	17.277451	2.64117647
SD	3.69674191	0.4735034

siLATS1 rapa

	yellow	red
	8.2	23.2
	9.5	22.25
	8.5	23.5
	11.3333333	31.6666667
	10.2	17.8
	10.5	25.1666667
	12.6666667	24.6666667
	6.4	22.6
	6.83333333	15.1666667
	7.33333333	20.6666667
	11.4	28.2
	13.25	22.75
	9	40.5
	4.75	20.25
	9.66666667	25.3333333
	7.25	30.25
	10.3333333	33.6666667
	10.25	32.25
average	9.29814815	25.5490741
SD	2.23716301	6.22491954

siLATS1 rapa+CQ

	yellow	red
	41.75	3.25
	46.2	2.2
	42.8	5.8
	38.5	4.66666667
	37.3333333	5.66666667
	33.6	3.8
	26.3333333	2.66666667
	30.25	3.25
	45.1666667	2.83333333
	34.8	6.2
	42.6	5.4
	30.3333333	4.66666667
	31.25	3.25
	34.8	5.2
	37.3333333	4.66666667
	31.6666667	2.66666667
	34.2	7.2
	29.8333333	4.83333333
average	36.0416667	4.34537037
SD	5.79335671	1.41966586

Suppl. Figure 6

a

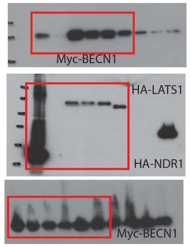

b

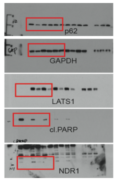

c

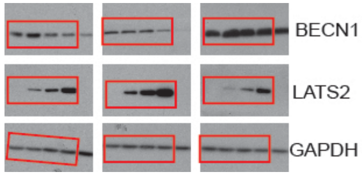

d

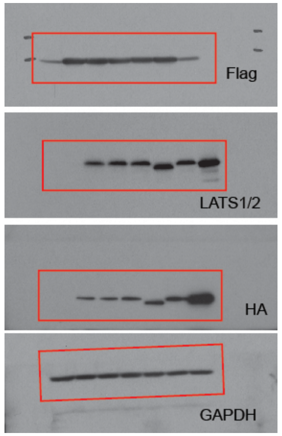

e

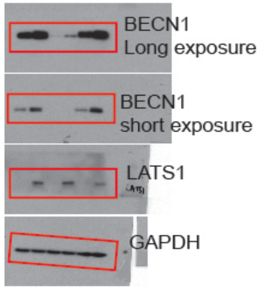

f

	EV	LATS1	siControl	siLATS1
exp 1	1	0.84498638	-0.089	0.9401742
exp2	1	1.12740041	-0.201	0.86994735
exp3	1	1.03741994	0.132	1.09581177
	average	1.00326891	average	0.96864444
	SD	0.14427106	SD	0.11559238

g

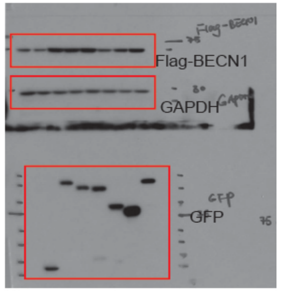

h

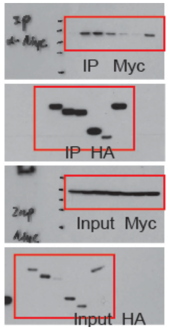

i

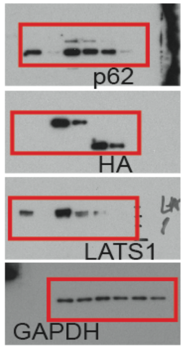

Suppl. Figure 7

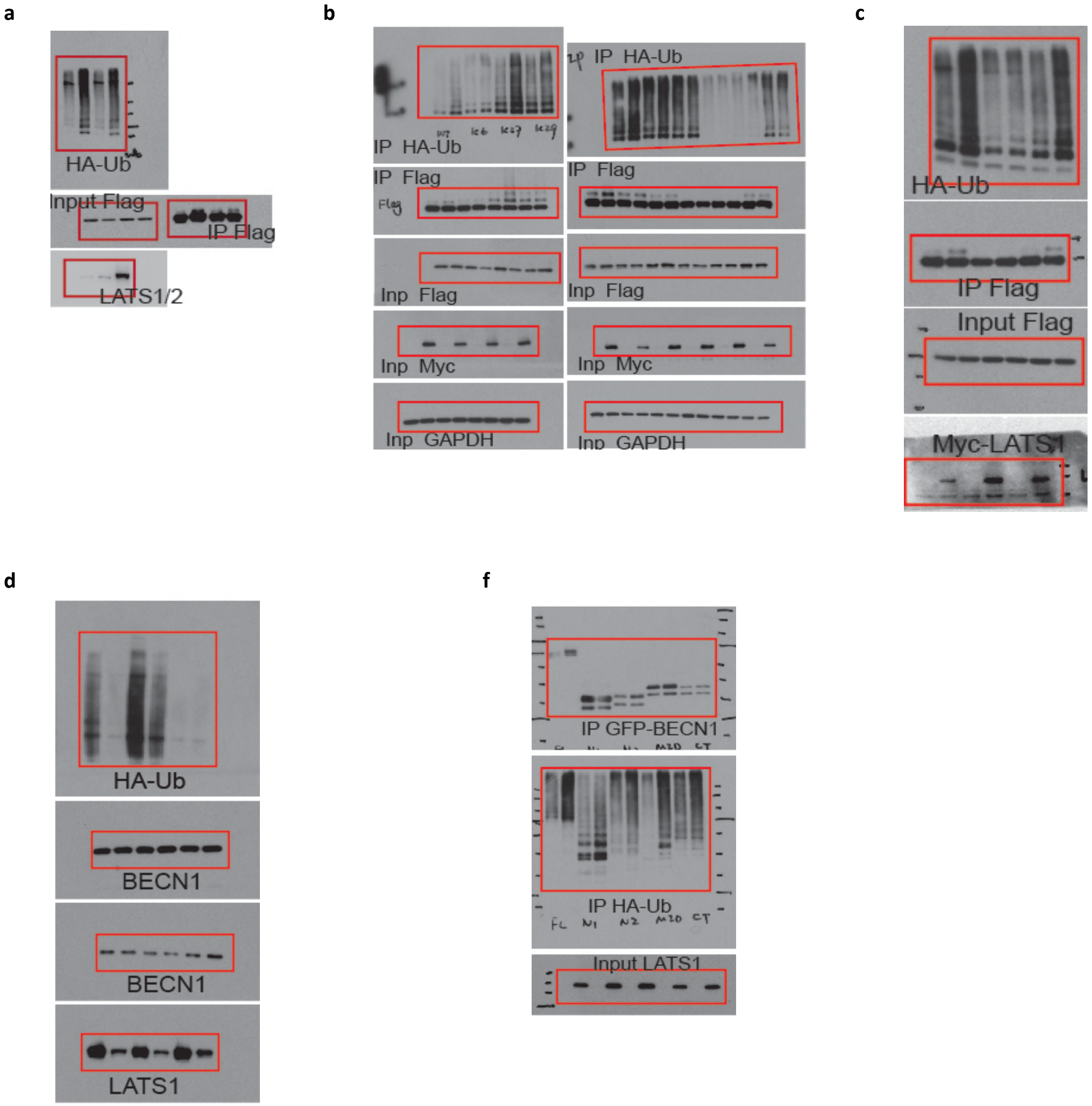

Suppl. Figure 8

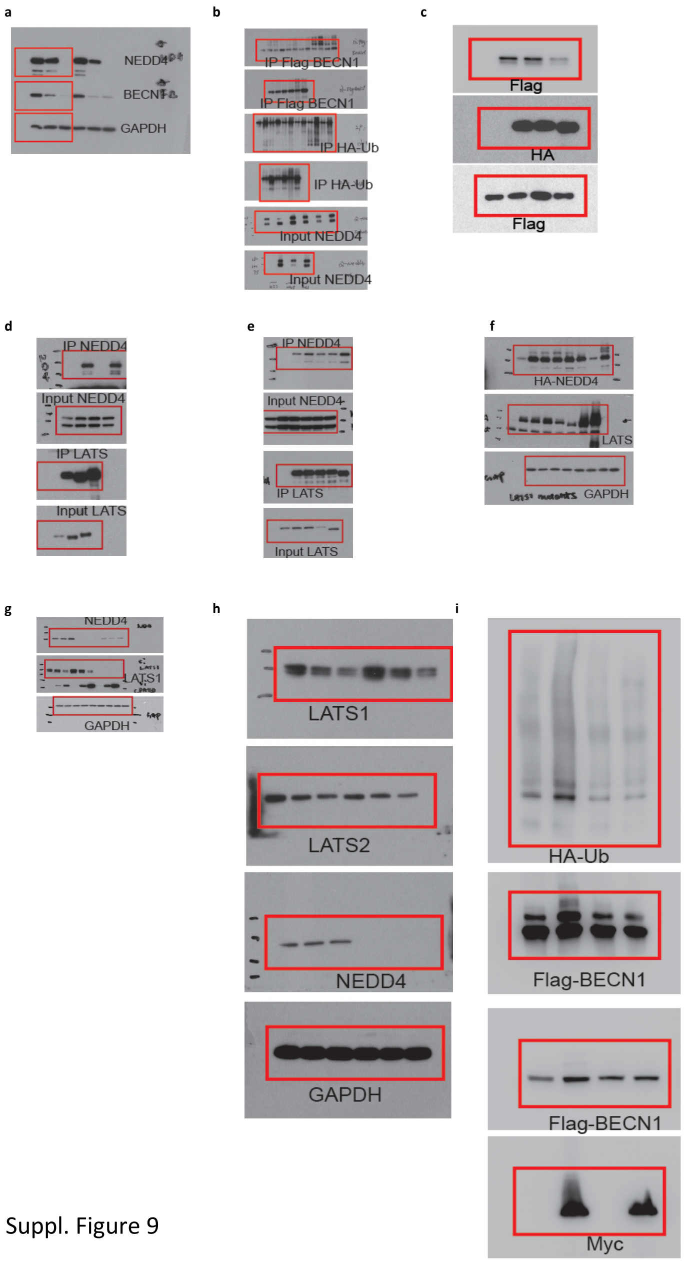

Suppl. Figure 9

c

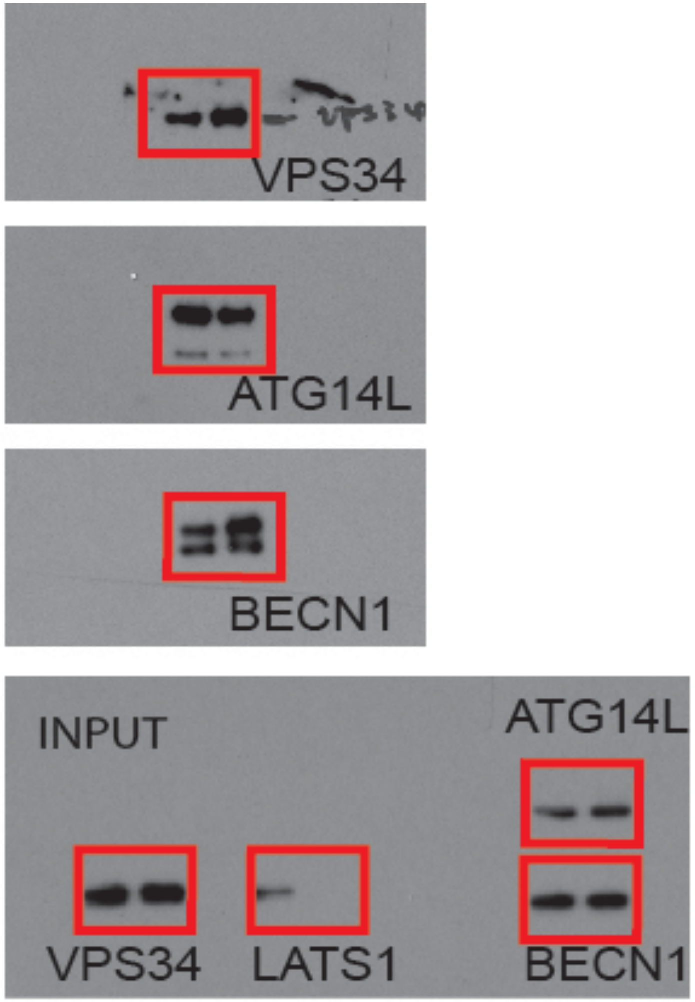

Suppl. Figure 10
